# Monitoring populations at increased risk for SARS-CoV-2 infection in the community using population-level demographic and behavioural surveillance

**DOI:** 10.1016/j.lanepe.2021.100282

**Published:** 2021-12-12

**Authors:** Emma Pritchard, Joel Jones, Karina-Doris Vihta, Nicole Stoesser, Prof Philippa C. Matthews, David W. Eyre, Thomas House, John I Bell, Prof John N Newton, Jeremy Farrar, Prof Derrick Crook, Susan Hopkins, Duncan Cook, Emma Rourke, Ruth Studley, Prof Ian Diamond, Prof Tim Peto, Koen B. Pouwels, Prof A. Sarah Walker, COVID-19 Infection Survey Team

**Affiliations:** a The National Institute for Health Research Health Protection Research Unit in Healthcare Associated Infections and Antimicrobial Resistance, University of Oxford, Oxford, UK; b Nuffield Department of Medicine, University of Oxford, Oxford, UK; c Office for National Statistics, Newport, UK; d Department of Engineering, University of Oxford, Oxford, UK; e The National Institute for Health Research Oxford Biomedical Research Centre, University of Oxford, Oxford, UK; f Department of Infectious Diseases and Microbiology, Oxford University Hospitals NHS Foundation Trust, John Radcliffe Hospital, Oxford, UK; g Big Data Institute, Nuffield Department of Population Health, University of Oxford, Oxford, UK; h Department of Mathematics, University of Manchester, Manchester, UK; i IBM Research, Hartree Centre, Sci-Tech Daresbury, UK; j Office of the Regius Professor of Medicine, University of Oxford, Oxford, UK; k Health Improvement Directorate, Public Health England, London, UK; l Wellcome Trust, London, UK; m Healthcare-Associated Infection and Antimicrobial Resistance Division, Public Health England, London, UK; n National Institute for Health Research, Health Protection Research Unit in Healthcare Associated Infections and Antimicrobial Resistance, Imperial College London, London, UK; o Health Economics Research Centre, Nuffield Department of Population Health, University of Oxford, Oxford, UK; p MRC Clinical Trials Unit at UCL, UCL, London, UK

**Keywords:** SARS-CoV-2, community, monitoring

## Abstract

**Background:**

The COVID-19 pandemic is rapidly evolving, with emerging variants and fluctuating control policies. Real-time population screening and identification of groups in whom positivity is highest could help monitor spread and inform public health messaging and strategy.

**Methods:**

To develop a real-time screening process, we included results from nose and throat swabs and questionnaires taken 19 July 2020-17 July 2021 in the UK's national COVID-19 Infection Survey. Fortnightly, associations between SARS-CoV-2 positivity and 60 demographic and behavioural characteristics were estimated using logistic regression models adjusted for potential confounders, considering multiple testing, collinearity, and reverse causality.

**Findings:**

Of 4,091,537 RT-PCR results from 482,677 individuals, 29,903 (0·73%) were positive. As positivity rose September-November 2020, rates were independently higher in younger ages, and those living in Northern England, major urban conurbations, more deprived areas, and larger households. Rates were also higher in those returning from abroad, and working in healthcare or outside of home. When positivity peaked December 2020-January 2021 (Alpha), high positivity shifted to southern geographical regions. With national vaccine roll-out from December 2020, positivity reduced in vaccinated individuals. Associations attenuated as rates decreased between February-May 2021. Rising positivity rates in June-July 2021 (Delta) were independently higher in younger, male, and unvaccinated groups. Few factors were consistently associated with positivity. 25/45 (56%) confirmed associations would have been detected later using 28-day rather than 14-day periods.

**Interpretation:**

Population-level demographic and behavioural surveillance can be a valuable tool in identifying the varying characteristics driving current SARS-CoV-2 positivity, allowing monitoring to inform public health policy.

**Funding:**

Department of Health and Social Care (UK), Welsh Government, Department of Health (on behalf of the Northern Ireland Government), Scottish Government, National Institute for Health Research.

**Research in Context**

**Evidence before this study**

Monitoring populations at increased risk for SARS-CoV-2 infection in the community is important in understanding infection spread and informing public health strategy. We searched PubMed and preprint archives MedRxiv and BioRxiv up to 31^st^ August 2021 for epidemiological journal articles using the terms (“coronavirus” OR “COVID*” OR “SARS-CoV-2”) AND (“positive” OR “positivity”) AND (“characteristic” OR “demographic”) AND (“screening” or “monitoring”) without data or language restrictions. Most studies we found assessed associations of specific demographics and/or behavioural characteristics with SARS-CoV-2 to answer targeted questions, rather than assessing a broad range of characteristics. Further, data used was often not representative of the community, such as hospital admissions data, or those self-reporting infection. A process to monitor a large and broad range of demographic and behavioural characteristics, such as in the current study, are required to understand current populations at increased risk of SARS-CoV-2 infection.

**Added value of this study**

This is a large community survey, monitoring populations at an increased risk of SARS-CoV-2 over a year. We describe key demographic and behavioural trends in a representative UK sample over a rapidly evolving pandemic, with emerging variants (including Alpha and Delta) and fluctuating control polices, including national lockdowns and the vaccine rollout programme. Importantly, the process presented in this paper allowed us to assess these trends weekly, meaning we could obtain regular estimates for key positivity associations. Using this process we found important independent associations between positivity and factors such as age, household size, geographical region, and working in health or outside the home during the rise of the Alpha variant (December 2020-February 2021), and sex and vaccination status during the Delta period (May 2021 onwards). Our methods allowed us to continuously monitor and summarise these associations over the study-period.

**Implications of all the available evidence**

This study demonstrates a process to monitor and identify key societal factors and specific behaviours associated with SARS-CoV-2 positivity in real-time. Rapidly identifying groups where positivity is rising can help monitor spread of infection, aiding policy development and targeted advice to control SARS-CoV-2 transmission. For example, this could be used to target public health messages to detected groups to determine if that increased uptake of symptomatic and asymptomatic testing. Further, the methods presented in this paper are not limited to the demographics and characteristics used here, and could be broadened to incorporate both different exposures and outcomes, and could be applicable to different diseases. Using methods described in this paper, we were able to identify populations at increased risk of SARS-CoV-2 infection in real-time, and monitor important trends across the UK.

Alt-text: Unlabelled box

## Introduction

To 31^st^ August 2021, there have been over 216·3 million SARS-CoV-2 cases worldwide.^1^ Disparities in COVID-19 risk and outcomes based on demographics and behaviours have been described in the UK^2^^,^^3^ and globally,^4^^,^^5^ but emerging variants^6^ coupled with varying control policies, including differential vaccine roll-out programmes, reinforce the need to monitor characteristics of individuals “at increased risk” for SARS-CoV-2 infection continuously. For example, identifying groups in whom newly identified variants of concern are spreading in the community may be vital in preventing widespread transmission. In England, since 26^th^ March 2020, there have been three national lockdowns, a tiered system^7^ with varying restrictions in smaller geographical areas, and various other restrictions between these,^8^ all affecting behaviour and risk of acquiring and spreading SARS-CoV-2. Finding societal factors or specific behaviours where these restrictions are less effective may aid policy development. With restrictions being relaxed in many countries, rapidly identifying groups where positivity is rising in real-time can help monitor spread and target advice.

High-quality surveillance is challenging, particularly given the large proportion of asymptomatic SARS-CoV-2-infected individuals,^9^ with a balance between missing important but potentially imprecisely estimated signals (false-negatives) and noise (false-positives). With large datasets containing many potential risk factors, multiple testing is inevitably problematic,^10^ but standard approaches to building regression models restricting to smaller numbers of hypothesised associated factors risks missing true signals with a rapidly evolving pathogen and societal responses. The cumulative effect of missing data across many risk factors can mean substantial proportions of the original sample are excluded from penalised regression or backwards elimination, losing power,^11^ and risking bias if missingness depends on outcome.^12^ A method allowing numerous variable parametrisations of many individual variables would therefore be useful, provided collinearity and confounding can be avoided.^13^

Using the Office for National Statistics (ONS) COVID-19 Infection Survey, a large community-based surveillance study, we therefore developed a process analogous to a repeated point-prevalence survey design with the potential to monitor groups with highest SARS-CoV-2 positivity week by week.

## Methods

### Study design

The ONS COVID-19 Infection Survey is a large household survey with longitudinal follow-up (ISRCTN21086382; https://www.ndm.ox.ac.uk/covid-19/covid-19-infection-survey/protocol-and-information-sheets). Private households are randomly selected on a continuous basis from address lists and previous surveys to provide a representative sample across the UK. Following verbal consent, a study worker visited each household to take written informed consent for individuals aged ≥2 years (from parents/carers for those 2–15 years; those 10–15 years also provided written assent). The study received ethical approval from the South Central Berkshire B Research Ethics Committee (20/SC/0195).

Participants were asked about demographics, behaviours, work, and vaccination uptake (https://www.ndm.ox.ac.uk/covid-19/covid-19-infection-survey/case-record-forms). At the first visit, participants were asked for consent for optional follow-up visits every week for the next month, then monthly thereafter. At each visit, participants provided a nose and throat self-swab. Most (>80%) included participants were visited each month from July 2020-March 2021, with this number decreasing to between 65-78% April-June 2021, as some completed one year's follow-up and chose not to extend their participation (**Supplementary Figure 1**). Details on survey response rate are available online (https://www.ons.gov.uk/peoplepopulationandcommunity/healthandsocialcare/conditionsanddiseases/datasets/covid19infectionsurveytechnicaldata; Table 2a-2f) as are comparisons of representativeness with the general population (https://www.ons.gov.uk/peoplepopulationandcommunity/healthandsocialcare/conditionsanddiseases/methodologies/coronaviruscovid19infectionsurveyqmi).

### Inclusion/exclusion criteria

This analysis included visits from 19^th^ July 2020-17^th^ July 2021 with a positive or negative swab result, including one visit per participant within each discrete fortnight in this period, namely the first test-positive visit, otherwise the last (negative) visit. This mimics repeated point-prevalence surveys, similar to the English Real-time Assessment of Community Transmission (REACT) study.^14^

### Outcome and exposures

The outcome was any SARS-CoV-2 PCR-positive swab in each fortnight. For exposures, we identified eight non-missing key potential confounders (“core” variables): sex, ethnicity (white vs non-white as relatively small numbers in the latter), age (years), geographical region (12 levels; 9 English regions and 3 devolved administrations: Wales, Scotland, Northern Ireland), rural/urban classification (major urban area, urban town/city, rural town, and rural village), deprivation percentile (derived separately for each country^15^, ^16^, ^17^, ^18^), household size, and whether the household was multigenerational (details in **Supplementary Methods**).

We next defined 60 non-core “screening” variables that could dynamically identify those at increased risk of testing positive (**Supplementary Table 1**), from questions detailing participant's current work/school status, including ability to social distance and patient-facing healthcare/social-care roles, current health status including COVID-19 vaccination and smoking, household and living environment, and contacts including with care homes, hospitals, and confirmed COVID-19 cases.

Although participants are tested predominantly monthly, most behavioural questions relate to the last 7 days. As some participants already know/think they have COVID-19 (from symptoms or testing outside the study) this could affect behaviours reported immediately before study tests, leading to reverse causality. The screening variables were therefore grouped into those most plausibly preceding any current infection (47 variables), or potentially modified through knowledge of recent prior infection (13 variables, including social/physical contacts, frequency of shopping and/or socialising, time spent in others homes/other people spent in participants’ homes; **Supplementary Table 1B**). For the latter, rather than the self-report at the included visit, we considered the maximum reported value across all visits in the preceding 35 days, excluding the included visit, and included only participants with at least one negative visit in the preceding 10-35 days.

### Statistical analysis

Within each fortnight, associations between SARS-CoV-2 positivity and the eight “core” characteristics were estimated using logistic regression (numbers included per fortnight in **Supplementary Table 2**). These characteristics were included in all subsequent models regardless of statistical significance. All analysis used complete-cases (all “core” variables were non-missing); models with household-level random effects would not converge due to low positivity rates. For geographic region, South West England was the reference as this had the lowest SARS-CoV-2 positivity across the study, facilitating identification of where infections were increasing. Given the large number of effect estimates over the 52-week study period (e.g. shown for urban/rural classification in **Supplementary Figure 2**), we summarised the importance of each characteristic over time using two properties simultaneously: 1) global (Wald) p-value and 2) overall effect size, the standard error-weighted mean effect estimate setting the reference to the level with lowest positivity in each fortnight:^19^

$$\begin{array}{c} \mathrm{Over}\;\mathrm{all}\;\mathrm{effe}\mathrm{ct}\;\mathrm{size}\;=\;\exp\left(\frac{\sum \frac{1}{\mathrm{se}\left(\beta_{i}\right)}\beta_{i}}{\sum \frac{1}{\mathrm{se}\left(\beta_{i}\right)}}\right),\\ \mathrm{where}\;\beta_{i}\;\mathrm{is}\;\mathrm{the}\;\log\;\mathrm{odds}\;\mathrm{ratio}\;\mathrm{for}\;\mathrm{each}\;\mathrm{level}. \end{array}$$

To incorporate non-linear effects, a restricted natural cubic spline was used for age (details in **Supplementary Methods**); the overall effect size combined estimates at ages 10, 25, 40, 55 vs 70 years (reference category) as above.

We tested interactions between the eight core variables individually in fortnights where positivity was >0·5% (arbitrary threshold to avoid small numbers), conducting backwards elimination on all with individual global heterogeneity p-value<0·001 (Bonferroni adjustment, 0·05/26 (number of interaction tests)), creating the “core model” (details in **Supplementary Methods**). An overall effect size was calculated for interactions as above, but taking the absolute coefficient values.

Given missing data (**Supplementary Table 1**), we used forward selection to retain as many participants as possible when screening each non-core characteristic based on complete-cases, first adding each of the 47 “screening” variables individually to the “core model”, thus estimating the total effects not explained by core characteristics. For all work-related variables, work status was included regardless of significance so that effects reflected additional effects of the characteristic for those currently employed and working. To monitor multiple testing, we plotted observed p-values (global per variable and individual level vs reference) against expected p-values assuming no difference (randomly distributed between 0 and 1 given the number of tests), creating a Q-Q plot, including 0·05, Bonferroni and Benjamini-Hochberg adjusted p-values (0·05/tests) as references. As the goal was to identify signals of “at-risk” populations, we included all characteristics with either global p<0·05 or any level with p<0·001 vs reference, and then used backward elimination (exit p=0·05) to identify a final “main model”. We used a similar process on the behavioural variables, also adjusting for variables identified from the main screen, regardless of significance. We categorised screening variables after backwards elimination into five broad groups dependent on persistence of effects:

• **Never:** The effect is never significant at a p<0·05 threshold in any fortnight
• **Inconsistent**: The variable is significant at a p<0·05 threshold in at least one fortnight, but never with an odds ratio in a consistent direction in any consecutive fortnights
• **Isolated**: The variable is significant at a p<0·05 threshold in two consecutive fortnights at most once, and “never consecutive” at all other times
• **Comes/goes**: The variable is significant at a p<0·05 threshold in three or more consecutive fortnights, or two consecutive fortnights at least twice, and is not significant with a gap of at least three fortnights, or two gaps of two fortnights, if the effect appears again
• **Persistent**: The variable is significant at a p<0·05 threshold for the entire period after the first significant fortnight, with no more than one gap of two fortnights separating consistency of the effect.

### Sensitivity Analyses

To assess the impact of small numbers of positives in some fortnights on power, we repeated the process using 28-day periods. Given logistic regression can have higher bias and variability with low rates, and hence lose accuracy and precision,^20^ we also compared the core variables effect estimates with those from ridge regression (see **Supplementary Results**).

### Role of the funding source

The funder had no role in study design, data collection, data analysis, data interpretation, or writing of the report. All authors had access to all data reported in the study and accept responsibility for the decision to submit for publication.

## Results

Analyses included 4,091,537 RT-PCR results from nose and throat swabs from 482,677 individuals (median (IQR) swabs per participant=9 (6-11)) in 240,490 households (median (IQR) swabs per household per fortnight=2 (1-2)) from 19^th^ July 2020-17^th^ July 2021. 29,903 (0·7%) swabs were positive. Overall, the median (IQR) age was 52 years (33-66), 300,208 (7%) visits occurred in those reporting non-white ethnicity, 2,165,833 (53%) in females, 1,463,624 (36%) in major urban areas and 1,746,530 (43%) in urban cities/towns, most (1,735,618, 42%) in two-person households, and with a median deprivation percentile of 60 (34-81) (1=most deprived, 100=least deprived) (Table 1; screened variables in **Supplementary Table 1A,1B**). The highest positivity was 1·9% (95% CI 1·9-2·0%) 20^th^ December-2^nd^ January 2020, and the lowest 0·05% (0·03-0·08%) 2^nd^-15^th^ August 2020 (**Supplementary Figure 3a**). Numbers within each fortnight increased as the study expanded from August-October 2020,^21^ from 32,184 participants 19^th^ July-1^st^ August 2020 to a median 173,054 (IQR 168,171-195,031) from 27^th^ September 2020 onwards (**Supplementary Figure 4**).

**Table 1:** Characteristics of the core variables for visits included in analysis.

Characteristic	Positive, n (%) or median (IQR)	Negative, n (%) or median (IQR)	Total, n (%) or median (IQR)
**Age (years)**	43 (23, 58)	52 (33, 66)	52 (33, 66)
**Sex**			
Male	14,405 (48)	1,911,299 (47)	1,925,704 (47)
Female	15,498 (52)	2,150,335 (53)	2,165,833 (53)
**Ethnicity**			
White	26,702 (89)	3,764,627 (93)	3,791,329 (93)
Non-White	3,201 (11)	297,007 (7)	300,208 (7)
**Deprivation percentile**	54 (29, 78)	60 (34, 81)	60 (34, 81)
**Household (HH) size**			
One	3,842 (13)	675,623 (17)	679,465 (17)
Two	10,124 (34)	1,725,494 (42)	1,735,618 (42)
Three	5,797 (19)	657,828 (16)	663,625 (16)
Four	6,639 (22)	686,036 (17)	692,675 (17)
Five or more	3,501 (12)	316,653 (8)	320,154 (8)
**Multigenerational HH**			
No	27,311 (91)	3,796,655 (93)	3,823,966 (93)
Yes	2,592 (9)	264,979 (7)	267,571 (7)
**Rural/urban classification**			
Major urban area	14,044 (47)	1,449,580 (36)	1,463,624 (36)
Urban city/town	11,425 (38)	1,735,105 (43)	1,746,530 (43)
Rural town	2,445 (8)	435,296 (11)	437,741 (11)
Rural village	1,989 (7)	441,653 (11)	443,642 (11)
**Region**			
London	6,498 (22)	698,608 (17)	705,106 (17)
North West England	5,077 (17)	477,380 (12)	482,457 (12)
North East England	1,390 (5)	156,119 (4)	157,509 (4)
Yorkshire	2,861 (10)	343,353 (8)	346,214 (8)
West Midlands	2,266 (8)	311,661 (8)	313,927 (8)
East Midlands	1,893 (6)	264,293 (7)	266,186 (7)
South East England	2,986 (10)	531,594 (13)	534,580 (13)
South West England	1,332 (4)	320,869 (8)	322,201 (8)
East England	2,425 (8)	405,304 (10)	407,729 (10)
Northern Ireland	665 (2)	106,660 (3)	107,325 (3)
Wales	969 (3)	179,900 (4)	180,869 (4)
Scotland	1,541 (5)	265,893 (7)	267,434 (7)

Note: for deprivation percentile, 1=most deprived, 100=least deprived. Multigenerational household defined as households including individuals aged school year 11 or younger AND school year 12 to age 49 AND aged 50+

### Core model

From 19^th^ July-1^st^ August 2020, we found no evidence that any core variable was associated with positivity, potentially related to power given both low positivity (0·08% [95% CI 0·06-0·12%]) and sample size (32,184 swabs, 27 positive). The first characteristic associated with positivity was ethnicity, the only characteristic associated with positivity in the fortnights between 2^nd^-29^th^ August 2020 (Figure 1A, Figure1B), with 3·3 (1·1-10·0; p-value=0·034) and 3·5 (1·5-7·9; p-value=0·003) higher odds of positivity in those of non-white ethnicity, respectively.

**Figure 1A:**
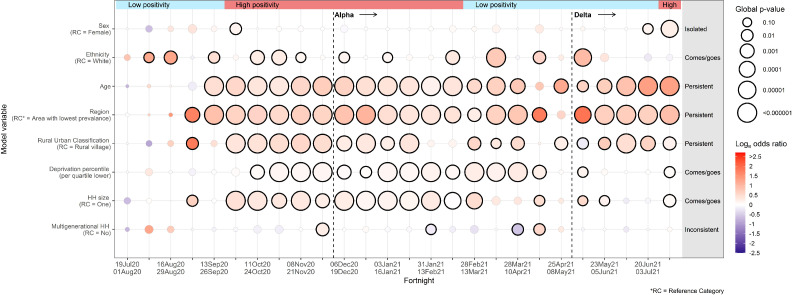
Overall effects of the 8 core variables across the 52 week study period. Note: RC=reference category. HH=household size. The size of the circles are proportional to -log_10_ of the global p-value for each variable in each fortnight. Circles with black outlines indicate p<0·05. The colour of the circles represents the size of the odds ratio (vs the reference category shown). For categorical variables with >2 levels (region, rural/urban classification, and household size), the reference category was set as the level with the lowest positivity in each fortnight, and the overall “odds ratio” calculated as: $\text{exp}(\frac{\sum \frac{1}{se(\beta_{i})}\beta_{i}}{\sum \frac{1}{se(\beta_{i})}})$. As age was included in the model as a restricted natural cubic spline, odds ratios were predicted at ages 10, 25, 40, and 55 vs 70 (reference) years and then combined in the same way. Numbers testing positive in each fortnight are provided in **Supplementary Table 2**. See **Methods** for details of classification as isolated, persistent etc.

**Figure 1B:**
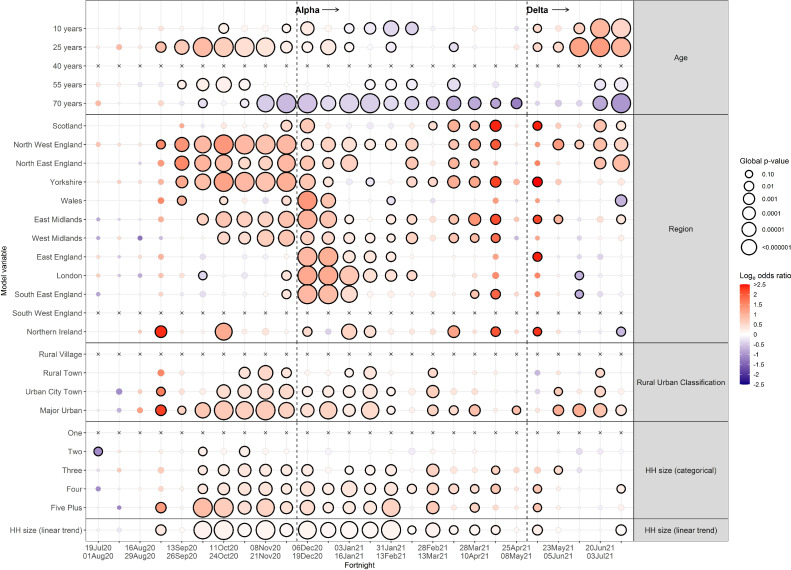
Effects of the 5 core variables with more than two categories across the 52 week study period.

As positivity began to increase early September 2020, geographical region, rural/urban classification, and household size became independently associated with positivity, with odds of positivity highest in Wales, Northern Ireland, and northern English regions, in more urban areas, and those living in larger households (Figure 1B). For most subsequent fortnights, evidence of higher positivity persisted in participants living in more urban areas, and larger households.

**Figure 2:**
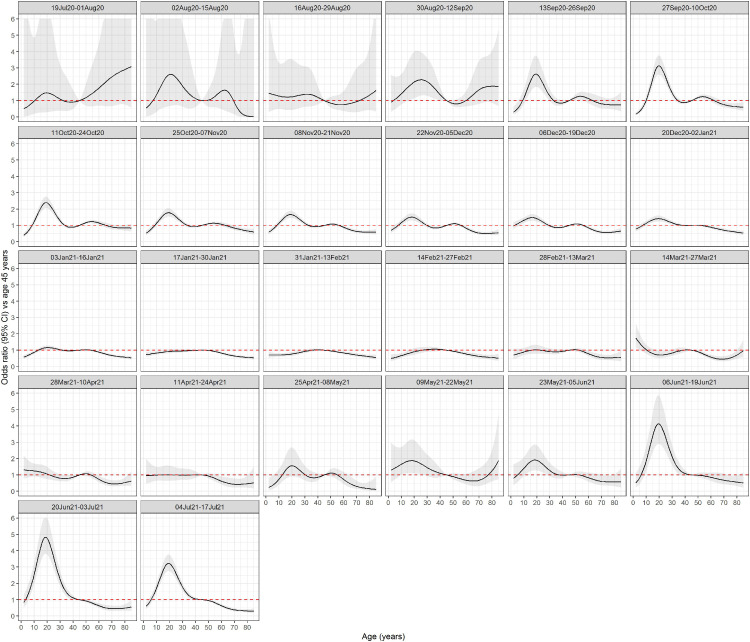
Adjusted effect of age (years) on positivity over the 52 week study period. Note: Odds ratios are predicted for each age vs a reference age of 45 years.

As positivity rates rose further through October 2020, age and deprivation became associated with positivity, with rates highest in those 16-30y, and living in more deprived areas. Positivity was also heavily concentrated in northern and then midland English regions until 21^st^ November 2020. From 22^nd^ November, positivity increased overall, particularly in southern England, with higher odds of positivity in London, East, and South East England, reflecting the rise of the Alpha variant.^22^ Age remained strongly associated with positivity, but with less excess risk at younger ages, and instead decreased odds of positivity in those over 60y (Figure 1B, Figure 2). This lower risk in older individuals persisted for most subsequent fortnights. During February-May 2021, as positivity decreased, associations between positivity and age, region, and deprivation persisted, but their strength attenuated. As positivity rose during 17^th^ May-17^th^ July 2021, reflecting the rise of the Delta variant^23^ and major sporting events, sex was associated with positivity in two consecutive fortnights for the first time in the study, with higher odds in males compared with females. Age again became strongly associated, with a large peak in those aged 16-30y (Figure 2).

Few interactions between core variables were significant at the p=0·001 threshold, with no evidence of the same significant interactions in any consecutive fortnight (**Supplementary Figure 5**). For model comparability, none were therefore included in any fortnight for screening other variables.

### Screening process

**Figure 3:**
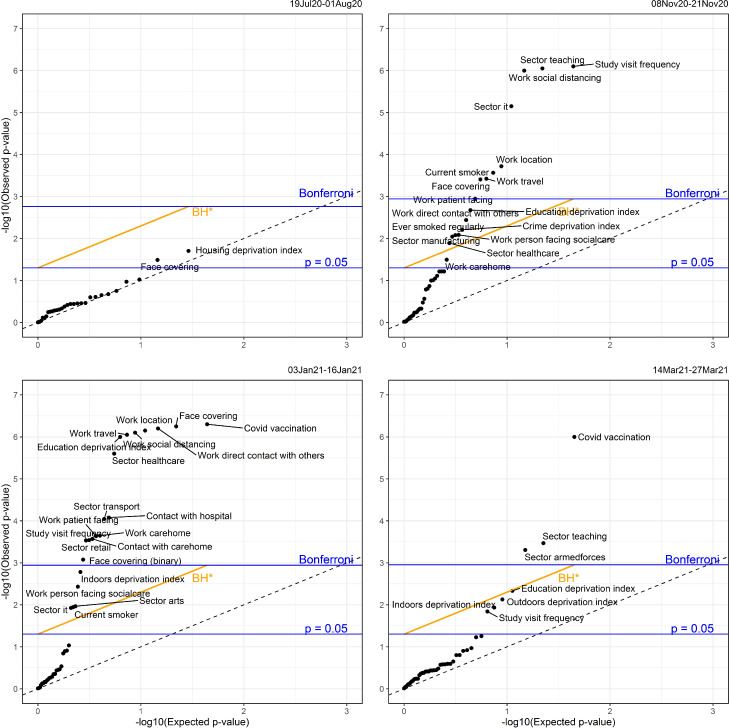
Global hetrogeneity p-values per factor from the screening process over 4 specific fortnights. *Benjamini-Hochberg threshold; calculated by ordering p-values from smallest to largest (k = 1,…n), and using the formula: *B-H threshold = k(0·05/N)*, where N is the total number of tests. Note: Black dashed line shows y = x. See Supplementary Table 1 for variable names and distributions. See **Supplementary Figure 17** for plots for all fortnights.

**Figure 4:**
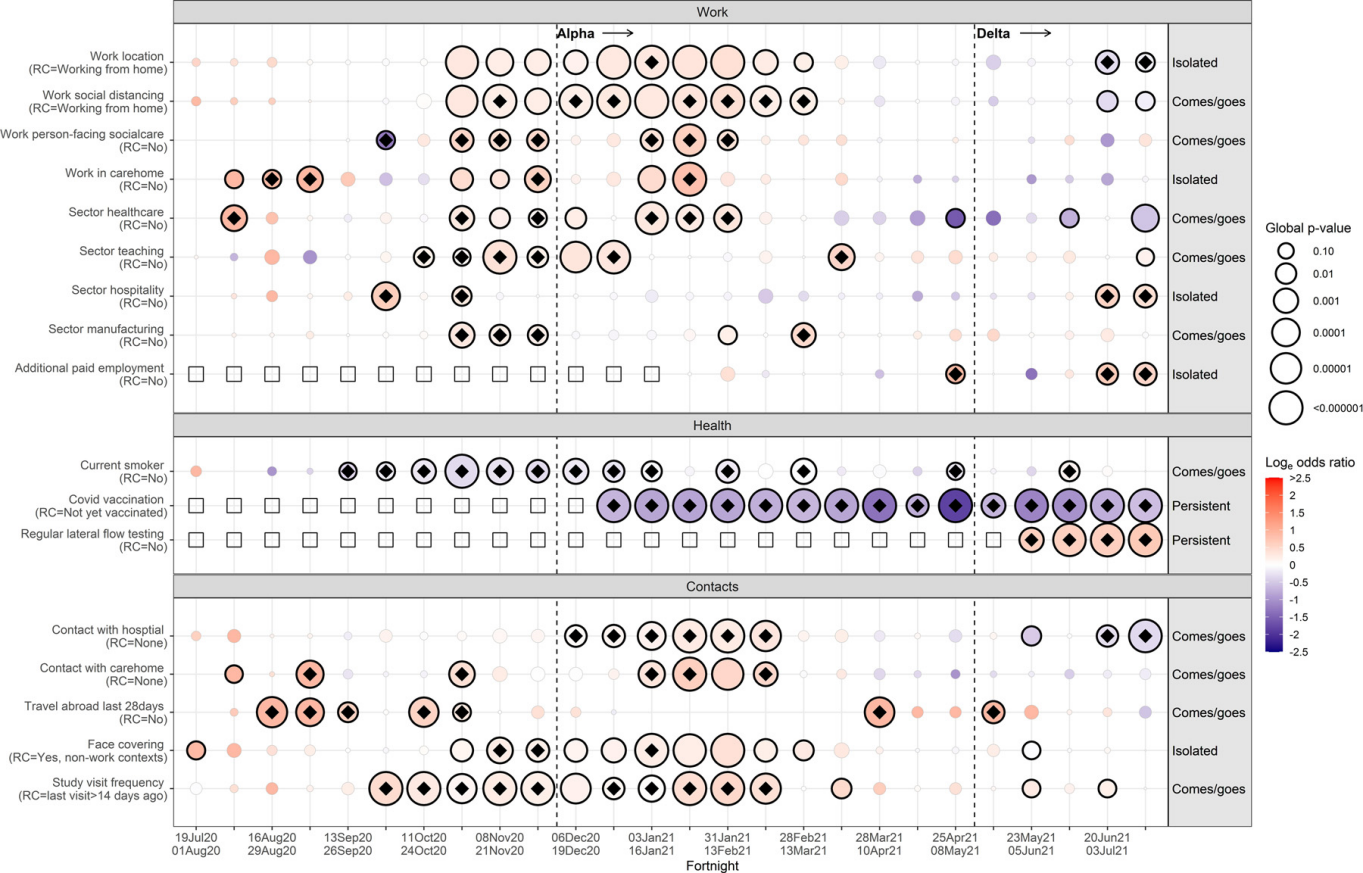
Overall effects of additional factors from the screening process, adjusted for the core variables, over the 52 week study period. Note: each factor included in addition to the core variables in each fortnight. Black diamonds indicate factors which remain after backswards elimination of all factors with p<0·05 in each fortnight. White squares indicate fortnights where characteristic was not collected by the survey. Categorisation of effect persistence (persistent, comes/goes, isolated) was done after backwards elimination. See **Supplementary Table 1** for variable names and distributions.

As positivity increased, the screening process identified more variables and at a greater significance than expected by chance (Figure 3; Figure 4; **Supplementary Figure 6**). Contact with anyone who had recently had COVID-19, currently self-isolating and thinking one had had COVID-19 recently, strongly and consistently predicted higher positivity. As these characteristics are potential mediators of effects of other factors, they were not considered further.

Work and employment were significantly associated with positivity throughout the study. Initially from 2^nd^ August-12^th^ September 2020, there was independently higher positivity for those working in care/nursing homes or patient-facing healthcare roles (Figure 4). This effect returned from 25^th^ October onwards, along with increased odds in those reporting working in healthcare sectors and specifically in person-facing social-care roles. From 25^th^ October 2020-27^th^ March 2021, we consistently observed higher positivity in those working outside compared with from home, with risk increasing as social distancing in the workplace became more difficult. Increased risk was also associated with all modes of travel to work (foot/bike, car/taxi, train/bus), compared with those not travelling to work (**Supplementary Figure 7**), with highest odds for car/taxi, then train/bus then foot/bike. Higher positivity was also observed in the teaching work sector during October/November 2020, while those working in IT had consistently lower odds (Figure 4**; Supplementary Figure 6**). As the Delta variant became prominent during June/July 2021, we observed lower (rather than higher) positivity in those reporting working outside the home.

From 16^th^ August-7^th^ November 2020, positivity was consistently higher in those who had travelled abroad in the last 28 days. This effect returned during 28^th^ March-12^th^ April 2021 and 9^th^-22^nd^ May 2021. Contact with hospital and care homes increased odds of positivity, particularly from 3^rd^ January-27^th^ February 2021, when positivity rates were very high due to Alpha. From 27^th^ September 2020-27^th^ February 2021 (when positivity was consistently >0·3%), participants were more likely to test positive on enrolment visits (**Supplementary Figure 7)**, most likely reflecting identification of longer-term PCR-positives at these visits.

Health-related variables varied in importance. Notably, there was no evidence of association between long-term health conditions and positivity. From 13^th^ September 2020-13^th^ March 2021, we consistently saw lower positivity in those who smoked tobacco products, compared with non-smokers. From 20^th^ December 2020, we observed a very strong effect of COVID-19 vaccination, with lower positivity in those vaccinated, compared with unvaccinated (**Supplementary Figure 7**). In a post-hoc analysis, since this would not generally be known in the community, prior infection (defined as a positive swab >120 days previously) also reduced the odds of positivity in the latter two fortnights (**Supplementary Table 3**). Deprivation components and living environment characteristics (available only for England) had little impact on positivity after adjusting for overall deprivation index and household size from the core model, likely due to high correlations between individual components with overall deprivation (**Supplementary Table 4; Supplementary Figure 8; Supplementary Results**).

**Figure 5:**
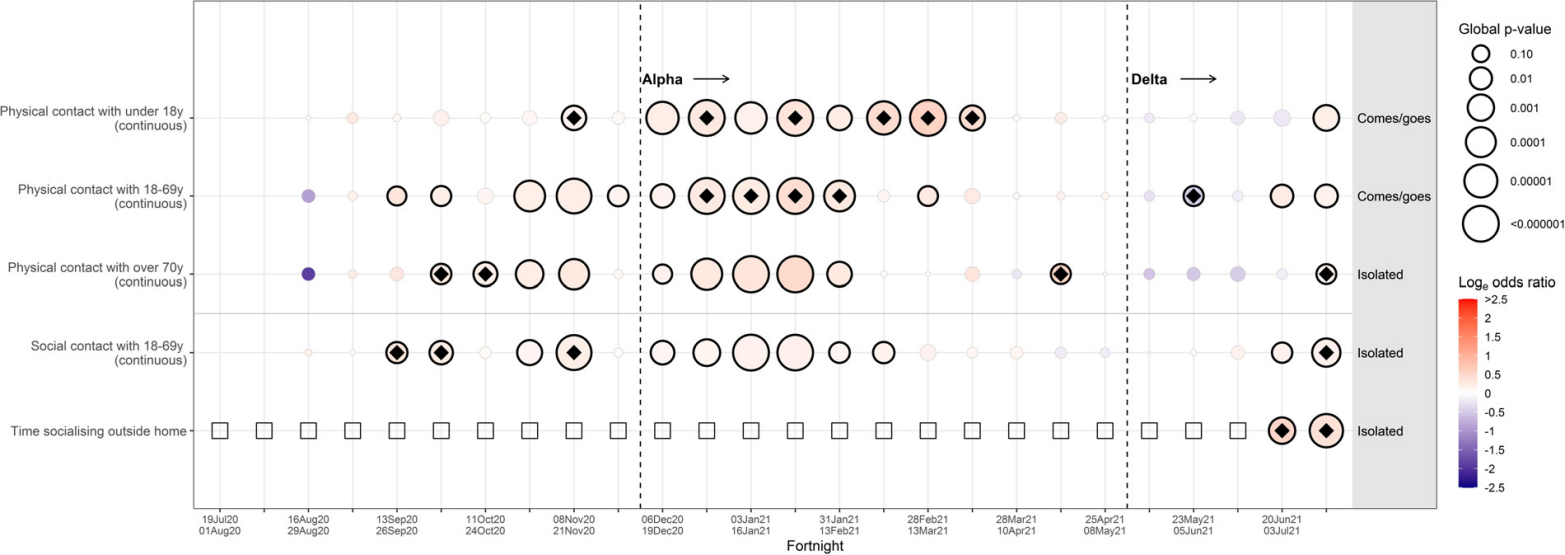
Adjusted effects of behavioural variables from the screening process. Note: each factor included in addition to the core variables in each fortnight. Black diamonds indicate factors which remain after adjustment for all variables identified in the main screen and backswards elimination of all factors with p<0·05 in each fortnight. White squares indicate fortnights where characteristic was not collected. Categorisation of effect persistence (persistent, comes/goes, isolated) was done after backwards elimination. See **Supplementary Table 1** for variable names and distributions.

Independently to the core model, we observed higher odds of positivity with increased social and physical contacts during periods when rates were high (Figure 5**; Supplementary Figure 9, 10**). After also adjusting for variables identified from the main screening process and after backwards elimination, we observed higher odds of positivity with higher numbers of physical contacts with 18-69 year olds between 20^th^ December 2020-13^th^ February 2021, and with higher numbers of physical contacts with those <18y between 14^th^ February 2021-27^th^ March 2021. As lockdown restrictions eased and Delta became prominent during 20^th^ June 2021-17^th^ July 2021, odds of positivity were higher in those with increasing time socialising outside home.

After backwards elimination, of the 71 variables screened (47 in the main screen, 13 variables in the behavioural screen with 24 parameterisations across the latter), two (3%) effects were persistent, 13 (18%) had effects which came and went, nine (13%) had effects isolated to only two consecutive fortnights, 30 (42%) were associated inconsistently in fortnights, and 17 (24%) were never associated.

### Sensitivity analysis

Similar key predictors of positivity were obtained using 28-day periods in the core model (**Supplementary Figures 11A, 11B, 12**). Notably, we saw a more consistent signal of higher positivity in non-white ethnicities from 11^th^ October 2020-27^th^ March 2021 (**Supplementary Figure 11A**), while this signal was more intermittent using fortnights (Figure 1A). We again did not see the same significant interactions in any consecutive 28-day periods (**Supplementary Figure 13A**). After backwards elimination, six interactions remained significant over five isolated 28-day periods (**Supplementary Figure 13B-G**). Three of these included household size, with a general pattern of stronger effects as household size increased in groups with higher positivity e.g. in younger ages (13^th^ September-10^th^ October 2020), non-white ethnicities (11^th^ October-7^th^ November 2020), and higher prevalence regions (6^th^ December 2020-2^nd^ January 2021). From 31^st^ January-27^th^ February 2021, compared with those living in non-multigenerational households, those of non-white ethnicities living in multigenerational households had increased odds of positivity, while those of white ethnicities had decreased odds.

Similar key associations were also identified from the screening process (**Supplementary Figure 14A, 14B**). Of the 45 consecutive occurrences of effects with p<0·05 in fortnights, 25 (56%) would have been detected later in 28-day periods, 14 (31%) at the same time, five (11%) earlier, and one (2%) never detected (**Supplementary Table 5**).

## Discussion

Over one year from 19^th^ July 2020-17^th^ July 2021, we estimated and summarised the key predictors of SARS-CoV-2 positivity in the UK, using a method designed to be run weekly in real-time to provide up-to-date information on changes in populations at increased risk. In the first fortnight from 19^th^ July-1^st^ August 2020, we had no evidence that any characteristic impacted positivity. As positivity rose through September-November 2020, positivity was independently higher in those of younger ages, living in Northern areas of England, in major urban conurbations, in more deprived areas, and in larger households. Additionally, rates were higher in those who had recently travelled abroad, worked in healthcare roles, or worked outside of home. As positivity peaked December 2020-January 2021, while we still observed strong effects of living in urban areas and large households, there was a major shift in high positivity to more southern geographical regions (reflecting the emergence of Alpha), with risk no longer concentrated in younger ages. Those working outside of home and in healthcare roles still had higher risk. As the national vaccine programme rolled out from December 2020, we saw large reductions in positivity in vaccinated individuals. From February-May 2021 as rates decreased, the impact of work on positivity decreased, while the effect of vaccination remained. As the Delta variant became prominent and positivity rates rose mid-May through July 2021, we observed higher odds of positivity in younger ages, in men, and in those not yet vaccinated.

Whilst our observed associations were consistent with other community infection surveys in the UK, particularly the English REACT study,^14^ no other studies have assessed as many characteristics in a community population over time as we were able to, with many focussed on outcomes of mortality and hospital admissions. Variation in positivity by region was well documented,^24^^,^^25^ as was increased positivity in non-white ethnic groups during September-November 2020,^24^ and those working in hospitals and care homes during November 2020-January 2021,^26^ but not as the Delta variant rose.^27^ As well as demonstrating the increased risk of infection in those not vaccinated as Delta came to dominate,^28^^,^^29^ the screening process will facilitate continuous monitoring of waning vaccine-associated protection going forward. We were also able to monitor characteristics including behaviours and work, many of which affected positivity inconsistently over time. For example, between October 2020-March 2021, working from home was associated with lower positivity, whereas during June/July 2021 working from home was associated with higher positivity. As working from home was recommended during the former period, working from home likely preceded infection. In contrast, as returning to the workplace became encouraged from May 2021, working from home may have sometimes been a consequence of exposure and hence self-isolation, leading to a degree of reverse causality, and a higher risk of positivity in those working from home. Supporting this, imputing work characteristics with working outside home if reported in the 35 days before current visit removed the higher positivity risk in those working from home (**Supplementary Figure 15**). Interpreting associations contextually with current restrictions is therefore critical (see **Supplementary Discussion** for other significant effects).

The screening process demonstrated here has several limitations. First, low event numbers and smaller sample sizes reduce statistical power, reducing the chance of detecting true associations (false-negatives) and increasing the likelihood that the magnitude of “true” effects are inflated (false-positives).^30^ Increased statistical power using 28-day periods rather than fortnights more consistently detected associations with ethnicity in the core model and found more evidence of interactions. The screening process, however, detected the same characteristics using both time-periods, with earlier detection in most cases using fortnights. As there were no major differences and we aimed to identify associations most relevant to current positivity, the benefit of more regular estimates may outweigh the power gained from evaluating longer time-frames, although this will depend on event numbers. When events numbers are low, logistic regression can be biased and/or imprecise.^31^^,^^32^ Sensitivity analyses using penalised regression techniques showed most coefficients were within the logistic regression confidence intervals, suggesting that, while there was some attenuation of estimates, for example for geographical regions in a few fortnights, the logistic regression models were not substantially overfitting.

Multiple testing is an unavoidable limitation of our screening process. Doing many multiple independent tests increases the risk of false-positives;^33^ however, a priori the questionnaire was based on potential risk factors so the “correct” degree of adjustment is unclear. We therefore used Q-Q plots with Bonferroni and Benjamini-Hochberg adjustments to monitor the potential for false-positives, rather than as strict thresholds.^34^^,^^35^ Even using stricter Bonferroni criteria, many screening variables were associated with positivity. Considering sex as a “negative control” (no effect expected), we only found an association in one of 24 fortnights before 20^th^ June 2021. The consistent association between sex and positivity from 20^th^ June-17^th^ July 2021 coincided with the European Football Championship, thus plausibly reflecting changes in social behaviour by sex, as observed elsewhere.^36^ Our results suggest more emphasis should be placed on effects that appear at least twice, interpreting effects that are inconsistent or appear sporadically with caution. Conversely, to avoid missing effects of specific work sectors due to the large number of levels (16), we included each work sector as an individual binary effect vs all other work sectors.

The underpinning design, namely a large community-based survey including randomly selected private households, is a major study strength. Participants being regularly asked about behaviours, work, and health status provided a rich opportunity to identify associations between positivity and many important demographic and behavioural characteristics. As participants were tested regardless of symptoms, characteristics could be assessed in an unbiased population, thus avoiding selection bias through only observing those choosing to take a COVID-19 test, for example, in the England national testing programme^37^ or through presenting to hospital with severe disease. Data quality and availability of key socio-demographic characteristics are important considerations if extending the screening process to different datasets. The study design also had limitations, particularly with individuals tested initially at weekly and then monthly visits. Some cases of SARS-CoV-2 occurring between visits were undoubtedly missed; however, we chose to only use study PCR-tests as our outcome to avoid introducing bias from those seeking additional tests outside the study. As fragments of virus can be detectable in the respiratory tract long after onset of infection, positives included in our outcome include both new infections and lingering PCR-positivity. Associations from the screening process may therefore not necessarily be related to new infections. Whilst we could have grouped positive tests into “episodes”, for example, considering only the first positive in 90-day periods,^38^ we chose to mirror other point-prevalence studies, such as REACT,^14^ also expecting that many characteristics would be reasonably stable over time and therefore even associations with ongoing PCR-positivity could still be relevant to the original infection. This may however dilute effects if participants with long carriage have different characteristics to those testing positive with new infections. Ongoing PCR-positivity may also reduce sensitivity to detect specific “at-risk” populations as new variants emerge.

In conclusion, the screening process presented could potentially be a valuable tool in understanding the characteristics driving current SARS-CoV-2 positivity, allowing us to provide enhanced up-to-date understanding of the pandemic across the UK. Looking forward, this could be used to target public health messages to detected groups to increased uptake of symptomatic and asymptomatic testing. We are using this method weekly to monitor the third wave of COVID-19 in the UK.

## Contributions

ASW, JF, JIB, JNN, IB, ID, and KBP designed and planned the study. EP, JJ, K-DV, NS, PCM, DE, TP, TH, DC, KBP, and ASW contributed to the statistical analysis. All authors contributed to interpretation of data and revised the report. All authors approved the final version of the report and agree to be accountable for all aspects of the work.

## Declaration of interests

DWE declares lecture fees from Gilead outside the submitted work. DC is a committee member for the International Development Section of the Royal Statistical Society, and a trustee for the Carers’ Hub Lambeth charity. No other author has a conflict of interest to declare.

## Appendix Supplementary materials

Image, application 1

Image, application 2

Image, application 3
