# Supplementary material for: Monitoring populations at increased risk for SARS-CoV-2 infection in the community using population-level demographic and behavioural surveillance
Source: Lancet Reg Health Eur. 2021 Dec 12;13:100282. doi: 10.1016/j.lanepe.2021.100282 (PMC8665900; doi:10.1016/j.lanepe.2021.100282)
Supplement: Supplementary file 1 [file mmc1.docx]

**Additional acknowledgements**

Office for National Statistics: Sir Ian Diamond, Emma Rourke, Ruth Studley, Tina Thomas, Duncan Cook.

Office for National Statistics COVID Infection Survey Analysis and Operations teams, in particular Daniel Ayoubkhani, Russell Black, Antonio Felton, Megan Crees, Joel Jones, Lina Lloyd, Esther Sutherland.

University of Oxford, Nuffield Department of Medicine: Ann Sarah Walker, Derrick Crook, Philippa C Matthews, Tim Peto, Emma Pritchard, Nicole Stoesser, Karina-Doris Vihta, Jia Wei, Alison Howarth, George Doherty, James Kavanagh, Kevin K Chau, Stephanie B Hatch, Daniel Ebner, Lucas Martins Ferreira, Thomas Christott, Brian D Marsden, Wanwisa Dejnirattisai, Juthathip Mongkolsapaya, Sarah Cameron, Phoebe Tamblin-Hopper, Magda Wolna, Rachael Brown, Sarah Hoosdally, Richard Cornall, David I Stuart, Gavin Screaton.

University of Oxford, Nuffield Department of Population Health: Koen Pouwels.

University of Oxford, Big Data Institute: David W Eyre, Katrina Lythgoe, David Bonsall, Tanya Golubchik, Helen Fryer.

University of Oxford, Radcliffe Department of Medicine: John Bell.

Oxford University Hospitals NHS Foundation Trust: Stuart Cox, Kevin Paddon, Tim James.

University of Manchester: Thomas House.

Public Health England: John Newton, Julie Robotham, Paul Birrell.

IQVIA: Helena Jordan, Tim Sheppard, Graham Athey, Dan Moody, Leigh Curry, Pamela Brereton.

National Biocentre: Ian Jarvis, Anna Godsmark, George Morris, Bobby Mallick, Phil Eeles.

Glasgow Lighthouse Laboratory: Jodie Hay, Harper VanSteenhouse.

Department of Health and Social Care: Jessica Lee.

Welsh Government: Sean White, Tim Evans, Lisa Bloemberg.

Scottish Government: Katie Allison, Anouska Pandya, Sophie Davis.

Public Health Scotland: David I Conway, Margaret MacLeod, Chris Cunningham.
