# Supplementary material for: Monitoring populations at increased risk for SARS-CoV-2 infection in the community using population-level demographic and behavioural surveillance
Source: Lancet Reg Health Eur. 2021 Dec 12;13:100282. doi: 10.1016/j.lanepe.2021.100282 (PMC8665900; doi:10.1016/j.lanepe.2021.100282)
Supplement: Supplementary file 2 [file mmc2.docx]

Supplementary Material

Supplementary file containing supplementary methods, results, and discussion, and all supplementary tables and figures.
