# Supplementary material for: Monitoring populations at increased risk for SARS-CoV-2 infection in the community using population-level demographic and behavioural surveillance
Source: Lancet Reg Health Eur. 2021 Dec 12;13:100282. doi: 10.1016/j.lanepe.2021.100282 (PMC8665900; doi:10.1016/j.lanepe.2021.100282)
Supplement: Supplementary file 3 [file mmc3.pdf]

Supplementary Material

SUPPLEMENTARY METHODS 3

Laboratory testing *3*

Variable and model specifications *3*

Deprivation 3

Age 3

Vaccination status 3

Interactions 3

Face covering variables 4

Parametrisation of categorical variables 4

SUPPLEMENTARY RESULTS 5

Ridge regression *5*

SUPPLEMENTARY DISCUSSION 6

SUPPLEMENTARY TABLES 8

Supplementary Table 1A: Characteristics of screening variables for visits included in the main screening process 8

Supplementary Table 1B Characteristics of screening variables for visits included in the behaviour screening process (B) 11

Supplementary Table 2: Count in each fortnight, including number not included in core model 13

Supplementary Table 3: Odds ratios and p-values for the effect of testing positive in the survey >120 days before current visit 14

Supplementary Table 4: Summary of individuals IMD components with combined index 15

Supplementary Table 5: Summary of p-values in 28-day periods for effects which occur in 2 or more consecutive fortnights 16

SUPPLEMENTARY FIGURES 17

Supplementary Figure 1: Number of participants followed-up per month of study period 17

Supplementary Figure 2: Log odds ratios with 95% confidence intervals for the effect of rural urban classification across the 52 week study period 18

Supplementary Figure 3: Unadjusted percentage (95% CI) of positive swabs per fortnight (A), and positive swabs split by gene positivity pattern (B) 19

Supplementary Figure 4: Total number of participants per fortnight 20

Supplementary Figure 5: Summary of odds ratio and p-values for interactions between all of the core variables using fortnights 21

Supplementary Figure 6 Overall effects of additional factors from the screening process which were either inconsistently or never associated with positivity, adjusted for the core variables, over the 52 week study period 22

Supplementary Figure 7: Effects of individual levels of factors from the screening process, adjusted for the core variables, over the 52 week study period 23

Supplementary Figure 8: Global hetergeneity p-values per factor from the screening process for household and living enviroment characteristics 26

Supplementary Figure 9: Overall effects of additional factors from the behavioural screening process which were either inconsistently or never associated with positivity, adjusted for the core variables, over the 52 week study period 27

Supplementary Figure 10: Individual p-values per factor from the screening process for behavioural characteristics 28

Supplementary Figure 11A: Summary of odds ratios and p-values for the 8 core variables over 28 day periods 29

Supplementary Figure 11B: Summary of odds ratios and p-values for the individual levels of the 8 core variables over 28 day periods 30

Supplementary Figure 12: Adjusted effect of age (years) on positivity using 28-day periods 31

Supplementary Figure 13A: Summary of odds ratio and p-values for interactions between all of the core variables for 28 day periods 32

Figure 13B: Effect of interaction of age by household size in the 28-day period 13 September to 10th October . 33

Figure 13C: Effect of interaction of ethnicity by household size in the 28-day period 11th October 2020 to 7th November 2020 34

Figure 13D: Effect of interaction of region by deprivation score in the 28-day period 8th November 2020 to 5th December 2020 34

Figure 13E: Effect of interaction of rural urban classification by age in the 28-day period 6th December 2020 to 2nd January 2021 35

Figure 13F: Effect of interaction of region by household size in the 28-day period 6th December 2020 to 2nd January 2021 36

Figure 13G: Effect of interaction of ethnicity by multigenerational households in the 28-day period 31st January 2021 to 27th February 2021 ... 37
Supplementary Figure 14A: Global heterogeneity p-values per factor from the screening process for 28-day periods for characetrics based on work, health status and contacts ... 38
Supplementary Figure 14B: Global heterogeneity p-values per factor from the screening process for 28-day periods for characteristics based on household and living environment ... 39
Supplementary Figure 15: Comparison of odds ratios and p-values for work location and work social distancing using reported answer from current visit (original) and using imputed values (see note below) ... 40
Supplementary Figure 16: Results from ridge regression and logistic regression ... 41
Supplementary Figure 17: Global hetergeneity p-values per factor from the screening process over all 26 fortnights ... 42

Supplementary Methods

Laboratory testing

Swabs were couriered directly to the United Kingdom's national Lighthouse laboratories (Glasgow (from 16 August 2020 onward) and the National Biocentre in Milton Keynes (from 26 April 2020 to 8 February 2021)) where samples were tested within the national testing program using identical methodology. The presence of three SARS-CoV-2 genes (ORF1ab and the genes transcribing nucleocapsid protein (N) and spike protein (S)) was identified using RT-PCR with the TaqPath RT-PCR COVID-19 kit (Thermo Fisher Scientific), analyzed using UgenTec FastFinder 3.300.5 (TaqMan 2019-nCoV Assay Kit V2 UK NHS ABI 7500 v2.1; UgenTec). The assay plugin contained an assay-specific algorithm and decision mechanism allowing conversion of the qualitative amplification assay raw data into test results with little manual intervention. Samples were called positive if either N or ORF1ab, or both, were detected. The S gene alone was not considered a reliable positive but could accompany other genes (that is, one, two or three gene positives).

Variable and model specifications

Deprivation

Deprivation was assessed using the index of multiple deprivation (IMD) in England, a score based on lower layer super output areas with average population of 1500 people and incorporating seven domains to produce an overall relative measure of deprivation (income, employment, education, skills and training, health and disability, crime, barriers to housing services and living environment) (https://www.gov.uk/government/statistics/english-indices-of-deprivation-2019). These sub-components were also assessed in the variable screening process, restricted to England. Equivalent scores were used in the other three countries comprising the UK[1-4]. Each country's scores were converted to a within country percentile. Therefore the effect of higher or lower deprivation is calibrated within country and will not necessarily be consistent across countries (i.e. the difference between the 10th to 20th percentile may be slightly different in different countries), and absolute percentiles may also not be exactly equal. All models including deprivation also included country and region as main effects and tested for interactions between country/region and deprivation to assess the impact of this (see **Supplementary Figure 5**). All other variables were consistent across the UK nations.

Age

Age was included in the model as a natural cubic spline with 4 internal knots at 20, 40, 60, 80th percentiles of unique ages, and boundary knots at 5th and 95th percentiles.

Vaccination status

Participants were asked about their vaccination status at visits, including the type, number of doses and date(s). Participants from England were also linked to administrative records from the National Immunisation Management Service (NIMS). We used records from NIMS where available. Otherwise, we used records from the survey, since linkage was periodic and NIMS does not contain information about vaccinations received abroad or in Northern Ireland, Scotland and Wales. Where records were available from both NIMS and the survey, agreement on type was 98% and agreement on dates was 95% within ±7 days.

Interactions

Interactions between household size and multigenerational households, and region and rural/urban classification were not considered as, by definition, all those living in multigenerational households had a household size of 3 or more, and not all regions included major urban conurbations.

Face covering variables

Prior to 18th February, participants in the study were asked the following question regarding face coverings: "*Do you mainly wear any kind of face covering or mask when you are outside your home, because of COVID-19?*" with the options:

- "No",
- "Yes, at work/school only",
- "Yes in other situations only (including public transport, shops)",
- "Yes, usually both at work/school and in other situations"
- "My face is already covered for other reasons (e.g. religious or cultural reasons)"

As of 18th February this question was retired, and participants were instead asked the two following questions about face coverings: "*Do you wear any kind of face covering or mask when you are at work/your place of education, because of COVID19?"*, and "*Do you wear any kind of face covering or mask when you are in other enclosed public spaces, such as shops, or using public transport, because of COVID-19?*", with the first options being either "Not going to place of work or education", or "Not going to place of work or education". This question caused similar issues with reverse causality as other behavioural questions, and hence these new questions were including in our behavioural screen, while the former question was included in the main screen.

Parametrisation of categorical variables

We incorporated work sector into the screening process as 16 separate binary variables (each work sector vs all other work sectors) rather than a 16 level categorical variable because the expectation was that only one or two sectors might have higher or lower positivity, with little difference between most sectors. Inclusion as one 16-level variable could risk missing effects of important individual work sectors versus an arbitrary reference category given the 16 degree of freedom global test, and, dependent on the reference category, individual effects may not be significant enough to capture at the individual p-value level. Instead the chosen parameterisation compares participants in each work sector to those currently working in all other work sectors. We treated all other categorical variables (with maximum of 5 levels) as mutually exclusive categories with a fixed reference category.

Supplementary Results

While the deprivation score component reflecting education was consistently associated with positivity, as this effect was in the same direction as the main deprivation score in the core model, and was only available in England, it was not considered further (**Supplementary Figure 8)**.

Ridge regression

We found 43 of the 692 (6%) coefficients from the core models produced from ridge regression did not fall within the 95% of the equivalent coefficients obtained from logistic regression (**Supplementary Figure 16**). Of these, the majority (38 coefficients; 88%) were effects of geographical region. These were mostly in the first fortnights of the study period when event rates and sample size was smallest, and also during December 2020, where we observed strong regional effects due to the rise of the Alpha variant in the Southern regions of England. Many of the inconsistencies within geographical region occurred within the same fortnight i.e. either none or all of the effect estimates for geographic regions were within the confidence intervals.

The differences observed between coefficients in December 2020 while the Alpha variant was rising suggest that the ridge regression penalised early signal for the regional effect, while logistic regression models picked this up. While often challenging to distinguish between signal and noise, through triangulation with other data sources, the regional effects observed in logistic regression model were accurate and representative of rises in Alpha variant in London and the South East, while ridge regression missed this effect, hence justifying our choice of method.

Supplementary Discussion

Work sectors with evidence of higher positivity over time generally involved roles with more contact with others, namely teaching, hospitality, and manufacturing. This is consistent other studies showing increases in SARS-CoV-2 cases with close contacts,[5] and the behavioral factors that we observed were associated with positivity in the behavioral screen, with higher positivity in those with physical contact with under 18s, particularly when schools were began to re-opened in March 2021,[6] higher positivity for physical contact with those aged 18-69 and over 70s, social contact with those aged 18-69, and those spending more time socialising outside their home. Increased positivity associated with those reporting additional paid employment may also reflect increased propensity for such work to involve close contact. Increased positivity in those having had recent contact with hospitals and care-homes during December 2020-February 2021 is likely due to the high number of SARS-CoV-2 cases in these environments as the Alpha variant emerged and came to dominate.[7] The persistent increased risk of positivity in those self-reporting taking regular lateral flow tests is likely due to their use, as recommended, for those whose activities reflect increased risk such as working outside the home or in teaching, healthcare or social care.

The impact of close contact may also explain the increased risk observed in those not self-reporting wearing face-coverings, with evidence suggesting face-coverings are effective at reducing transmission.[8] The higher risk observed in those wearing face-coverings in work and other situations, compared with those only wearing face-coverings in non-work situations may reflect the increased risk of working away from home which we observed in other work-related characteristics. Higher positivity in those who had recently travelled abroad in August-November 2020 may also be due to the increased number of close contacts involved with travel (e.g. being at an airport), or higher risks of infection in the destination country at the time.

We observed a lower risk of positivity in those who smoke tobacco products consistently from September 2020 to January 2021 and intermittently from then onwards. We did not observe this protective effect in those only reporting vaping. While some have outlined biologically plausible mechanisms which may explain this reduced risk, this also could be attributed to residual confounding based on the demographic of people who smoke offering a protective effect.[9] Vaccination has been shown to reduce community infections elsewhere;[10] prior infection is well-recognised to give at least as good protection, and the fact that these known associations were easily identified within our model provides confidence that important confounders are adjusted for in estimating other associations.

Our study was also conducted over periods when both Alpha and Delta were prominent in the UK. Whilst theoretically this might have allowed us to estimate changes associated with each variant, the differing control polices over these periods, coupled with a large proportion of the population being vaccinated once Delta become prominent, make it challenging to disentangle whether different associations are due to control strategy versus variant.

REFERENCE LIST

1. Ministry of Housing CLG. English indices of deprivation 2019, 2019.
2. Northern Ireland Statistics and Research Agency. Northern Ireland Multiple Deprivation Measure 2017 (NIMDM2017), 2017.
3. Scottish Government. Scottish Index of Multiple Deprivation 2020, 2020.
4. Statistics for Wales. Welsh Index of Multiple Deprivation (full Index update with ranks): 2019, 2019.
5. Thompson HA, Mousa A, Dighe A, et al. Severe Acute Respiratory Syndrome Coronavirus 2 (SARS-CoV-2) setting-specific transmission rates: a systematic review and meta-analysis. *Clinical Infectious Diseases* 2021
6. Timmins N. Schools and coronavirus The government's handling of education during the pandemic: Institute for Government 2021.
7. Office for National Statistics. Coronavirus (COVID-19) latest insights: Hospitals 2021 [Available from: https://www.ons.gov.uk/peoplepopulationandcommunity/healthandsocialcare/conditionsanddiseases/articles/coronaviruscovid19latestinsights/hospitals accessed 07 Oct 2021.
8. Ueki H, Furusawa Y, Iwatsuki-Horimoto K, et al. Effectiveness of Face Masks in Preventing Airborne Transmission of SARS-CoV-2. *mSphere* 2020;5(5) doi: 10.1128/mSphere.00637-20 [published Online First: 2020/10/23]
9. Usman MS, Siddiqi TJ, Khan MS, et al. Is there a smoker's paradox in COVID-19? *BMJ evidence-based medicine* 2020
10. Elliott P, Haw D, Wang H, et al. REACT-1 round 13 final report: exponential growth, high prevalence of SARS-CoV-2 and vaccine effectiveness associated with Delta variant in England during May to July 2021. *medRxiv* 2021

Supplementary Tables

Supplementary Table 1A: Characteristics of screening variables for visits included in the main screening process

Characteristic	Positive, n (%) or median (IQR)	Negative, n (%) or median (IQR)	Total, n (%) or median (IQR)
**Contact with other people**			
**Contact with known Covid-19 (last 28 days)**			
No	13,999 (47)	3,640,835 (90)	3,654,834 (89)
Yes	15,904 (53)	420,799 (10)	436,703 (11)
Missing	0 (0)	0 (0)	0 (0)
**Contact hospital (last 28 days)**			
No	22,699 (76)	3,124,538 (77)	3,147,237 (77)
Yes, I have	3,677 (12)	500,711 (12)	504,388 (12)
No, but someone in my household has	2,967 (10)	359,387 (9)	362,354 (9)
Missing	560 (2)	76,998 (2)	77,558 (2)
**Contact carehome (last 28 days)**			
No	28,007 (94)	3,825,176 (94)	3,853,183 (94)
Yes, I have	623 (2)	77,503 (2)	78,126 (2)
No, but someone in my household has	592 (2)	67,317 (2)	67,909 (2)
Missing	681 (2)	91,638 (2)	92,319 (2)
**Travel abroad in the last 28 days**			
No	29,662 (99)	4,034,194 (99)	4,063,856 (99)
Yes	241 (1)	27,440 (1)	27,681 (1)
Missing	0 (0)	0 (0)	0 (0)
**Face covering**			
Yes, other situations only	15,479 (52)	2,394,819 (59)	2,410,298 (59)
Yes, work and other situations	10,254 (34)	1,224,461 (30)	1,234,715 (30)
Yes, work only	471 (2)	40,593 (1)	41,064 (1)
Yes, face already covered	632 (2)	52,980 (1)	53,612 (1)
No	1,746 (6)	188,210 (5)	189,956 (5)
Missing	1,321 (4)	160,571 (4)	161,892 (4)
**Face covering (binary)**			
Yes (any)	26,836 (90)	3,712,853 (91)	3,739,689 (91)
No	1,746 (6)	188,210 (5)	189,956 (5)
Missing	1,321 (4)	160,571 (4)	161,892 (4)
**Visit frequency**[‡]			
Last visit >14 days ago	19,043 (64)	2,863,978 (71)	2,883,021 (70)
Last visit <= 14 days ago	7,852 (26)	916,167 (23)	924,019 (23)
Enrollment	3,008 (10)	281,489 (7)	284,497 (7)
Missing	0 (0)	0 (0)	0 (0)
**Household and living environment**			
**IMD indoors***	50 (27, 73)	51 (27, 75)	51 (27, 75)
Missing	3,175 (11)	552,453 (14)	555,628 (14)
**IMD outdoors***	44 (20, 71)	51 (26, 76)	51 (26, 76)
Missing	3,175 (11)	552,453 (14)	555,628 (14)
**IMD education***	59 (34, 82)	64 (39, 84)	64 (39, 84)
Missing	3,175 (11)	552,453 (14)	555,628 (14)
**IMD health***	55 (29, 78)	62 (37, 82)	62 (37, 82)
Missing	3,175 (11)	552,453 (14)	555,628 (14)
**IMD crime***	49 (26, 72)	57 (32, 79)	57 (32, 79)
Missing	3,175 (11)	552,453 (14)	555,628 (14)
**IMD housing***	49 (26, 75)	49 (25, 75)	49 (25, 75)
Missing	3,175 (11)	552,453 (14)	555,628 (14)
**Number of people per room***	1 (0, 1)	1 (0, 1)	1 (0, 1)
Missing	3,633 (12)	562,589 (14)	566,222 (14)
**Number of people per bedroom***	1 (1, 1)	1 (1, 1)	1 (1, 1)
Missing	3,640 (12)	562,795 (14)	566,435 (14)
**Number of people per 100m2***	3 (2, 4)	2 (2, 3)	2 (2, 3)
Missing	3,669 (12)	566,273 (14)	569,942 (14)
**Energy efficiency decile***	6 (3, 10)	6 (3, 10)	6 (3, 10)
Missing	3,526 (12)	551,740 (14)	555,266 (14)
**Age of house (decades) ***	3 (3, 4)	3 (3, 4)	3 (3, 4)
Missing	14,910 (50)	2,174,664 (54)	2,189,574 (54)
**Work , school, and nursery**[§]			
**Work status**			
Employed, working	14,713 (49)	1,832,299 (45)	1,847,012 (45)
Employed, not working	1,858 (6)	134,876 (3)	136,734 (3)
Not working	1,631 (5)	213,550 (5)	215,181 (5)
Retired	5,455 (18)	1,281,213 (32)	1,286,668 (31)
Child/student	6,239 (21)	599,352 (15)	605,591 (15)
Missing	7 (0)	344 (0)	351 (0)
**Work location**			
Working from home	7,868 (26)	1,005,480 (25)	1,013,348 (25)

Characteristic	Positive, n (%) or median (IQR)	Negative, n (%) or median (IQR)	Total, n (%) or median (IQR)
Elsewhere	12,528 (42)	1,433,415 (35)	1,445,943 (35)
NA	8,511 (28)	1,537,192 (38)	1,545,703 (38)
Missing	996 (3)	85,547 (2)	86,543 (2)
**Work social distancing**			
Working from home	7,868 (26)	1,005,480 (25)	1,013,348 (25)
Elsewhere, easy to maintain 2m	3,239 (11)	437,667 (11)	440,906 (11)
Elsewhere, relatively easy to maintain 2m	1,826 (6)	214,528 (5)	216,354 (5)
Elsewhere, difficult to maintain 2m	2,004 (7)	214,690 (5)	216,694 (5)
Elsewhere, very difficult to maintain 1m	4,247 (14)	449,980 (11)	454,227 (11)
NA	8,511 (28)	1,537,192 (38)	1,545,703 (38)
Missing	2,208 (7)	202,097 (5)	204,305 (5)
**Work travel†**			
Working from home	7,868 (26)	1,005,480 (25)	1,013,348 (25)
On foot/bike or other	2,616 (9)	295,024 (7)	297,640 (7)
Car/taxi	7,986 (27)	937,529 (23)	945,515 (23)
Train/bus	1,413 (5)	137,124 (3)	138,537 (3)
NA	8,511 (28)	1,537,192 (38)	1,545,703 (38)
Missing	1,509 (5)	149,285 (4)	150,794 (4)
**Work direct contact patients, service users, clients, customers**			
No	25,962 (87)	3,630,423 (89)	3,656,385 (89)
Yes	3,685 (12)	404,714 (10)	408,399 (10)
Missing	256 (1)	26,497 (1)	26,753 (1)
**Ever reported working in person facing social care**			
No	29,464 (99)	4,020,303 (99)	4,049,767 (99)
Yes	439 (1)	41,331 (1)	41,770 (1)
Missing	0 (0)	0 (0)	0 (0)
**Ever reported working in care home**			
No	29,426 (98)	4,019,274 (99)	4,048,700 (99)
Yes	477 (2)	42,360 (1)	42,837 (1)
Missing	0 (0)	0 (0)	0 (0)
**Ever reported working in patient facing healthcare**			
No	29,031 (97)	3,970,666 (98)	3,999,697 (98)
Yes	872 (3)	90,968 (2)	91,840 (2)
Missing	0 (0)	0 (0)	0 (0)
**Work sector**			
Teaching and education	2,832 (9)	295,102 (7)	297,934 (7)
Health care	2,034 (7)	225,167 (6)	227,201 (6)
Social care	534 (2)	60,746 (1)	61,280 (1)
Transport (incl. storage, logistic)	752 (3)	77,628 (2)	78,380 (2)
Retail sector (incl. wholesale)	1,384 (5)	150,473 (4)	151,857 (4)
Hospitality (e.g. hotel, restaurant)	705 (2)	67,521 (2)	68,226 (2)
Food production, agriculture, farming	268 (1)	35,235 (1)	35,503 (1)
Personal services (e.g. hairdressers)	235 (1)	27,437 (1)	27,672 (1)
Information technology and communication	1,014 (3)	148,805 (4)	149,819 (4)
Financial services incl. insurance	1,303 (4)	168,590 (4)	169,893 (4)
Manufacturing or construction	1,737 (6)	195,676 (5)	197,413 (5)
Civil service or Local Government	1,087 (4)	143,774 (4)	144,861 (4)
Armed forces	50 (0)	6,847 (0)	6,897 (0)
Arts, Entertainment or Recreation	399 (1)	55,956 (1)	56,355 (1)
Other occupation sector	2,341 (8)	324,118 (8)	326,459 (8)
NA (not currently working)	9,863 (33)	1,534,348 (38)	1,544,211 (38)
Missing	3,365 (11)	544,211 (13)	547,576 (13)
**Additional paid employment**			
No	10,342 (35)	2,241,224 (55)	2,251,566 (55)
Yes	127 (0)	21,981 (1)	22,108 (1)
Missing	19,434 (65)	1,798,429 (44)	1,817,863 (44)
**Current health status**			
**Think have had covid (last 90 days)**			
No	10,288 (34)	3,970,284 (98)	3,980,572 (97)
Yes	19,615 (66)	91,350 (2)	110,965 (3)
Missing	0 (0)	0 (0)	0 (0)
**Self-isolating**			
No	20,121 (67)	3,804,735 (94)	3,824,856 (93)
Yes I or some in my HH is	8,003 (27)	24,497 (1)	32,500 (1)
Yes, other reasons	845 (3)	74,019 (2)	74,864 (2)
Missing	934 (3)	158,383 (4)	159,317 (4)
**Smoke now**			
Non-smoker	27,520 (92)	3,695,283 (91)	3,722,803 (91)
Tobacco smoker	1,583 (5)	268,245 (7)	269,828 (7)

Characteristic	Positive, n (%) or median (IQR)	Negative, n (%) or median (IQR)	Total, n (%) or median (IQR)
Only vape	693 (2)	82,037 (2)	82,730 (2)
Missing	107 (0)	16,069 (0)	16,176 (0)
**Smoke ever regularly**			
No	22,120 (74)	2,843,859 (70)	2,865,979 (70)
Yes	7,283 (24)	1,139,616 (28)	1,146,899 (28)
Missing	500 (2)	78,159 (2)	78,659 (2)
**Any disability**			
No	26,607 (89)	3,513,264 (86)	3,539,871 (87)
Yes	3,296 (11)	548,370 (14)	551,666 (13)
Missing	0 (0)	0 (0)	0 (0)
**Long-term health conditions**			
No	24,755 (83)	3,243,863 (80)	3,268,618 (80)
Yes	4,765 (16)	751,236 (18)	756,001 (18)
Missing	383 (1)	66,535 (2)	66,918 (2)
**Impact of health conditions**			
No health conditions	24,755 (83)	3,243,863 (80)	3,268,618 (80)
No impact at all	2,164 (7)	332,664 (8)	334,828 (8)
A little impact	1,526 (5)	239,834 (6)	241,360 (6)
A lot of impact	1,017 (3)	172,191 (4)	173,208 (4)
Missing	441 (1)	73,082 (2)	73,523 (2)
**Covid vaccination status**			
Not vaccinated, no prior positive, >21 days before vaccination	25,254 (84)	2,431,522 (60)	2,456,776 (60)
1-21 days before vaccination or 0-7 days post vaccination	1,422 (5)	313,585 (8)	315,007 (8)
Vaccinated 8-20 days ago	665 (2)	141,629 (3)	142,294 (3)
Vaccinated >= 21 days ago, no second dose	1,162 (4)	495,471 (12)	496,633 (12)
Post second dose or not vaccinated prior positive	1,400 (5)	679,427 (17)	680,827 (17)
Missing	0 (0)	0 (0)	0 (0)
**Regular LFT testing**			
No	719 (2)	59,169 (1)	59,888 (1)
Yes	1,055 (4)	116,773 (3)	117,828 (3)
Missing	28,129 (94)	3,885,692 (96)	3,913,821 (96)

*Characteristic available for England only

** Question introduced or expanded part way through the study so missing data also reflects time periods when the question was not included.

† 6,744/945,515 visits in the car/taxi group were taxi; numbers were too few to assess whether another grouping might be preferable.

‡Visit frequency was calculated based on completed survey visits

§ Questions on work, such as work location and work social distancing were asked phrased as “if currently working”.

Note: For more details on the questions from which these characteristics were derived, the current questionnaire used in the survey, as well as all previous versions can we found at: https://www.ndm.ox.ac.uk/covid-19/covid-19-infection-survey/case-record-forms.

Supplementary Table 1B Characteristics of screening variables for visits included in the behaviour screening process (B)

Characteristic†	Positive, n (%) or median (IQR)	Negative, n (%) or median (IQR)	Total, n (%) or median (IQR)
**Number of physical contacts aged <18**			
0	11,898 (40)	2,160,467 (53)	2,172,365 (53)
1-5	4,146 (14)	608,127 (15)	612,273 (15)
6-10	675 (2)	71,849 (2)	725,24 (2)
11-20	2,294 (8)	206,076 (5)	208,370 (5)
21 or more	10,890 (36)	1,015,115 (25)	1,026,005 (25)
Missing	11,898 (40)	2,160,467 (53)	2,172,365 (53)
**Number of physical contacts aged 18-69**			
0	10,031 (34)	1,848,906 (46)	1,858,937 (45)
1-5	6,487 (22)	950,800 (23)	957,287 (23)
6-10	1,233 (4)	128,817 (3)	130,050 (3)
11-20	1,269 (4)	119,866 (3)	121,135 (3)
21 or more	10,883 (36)	1,013,245 (25)	1,024,128 (25)
Missing	10,031 (34)	1,848,906 (46)	1,858,937 (45)
**Number of physical contacts aged >=70**			
0	15,293 (51)	2,530,655 (62)	2,545,948 (62)
1-5	3,034 (10)	449,008 (11)	452,042 (11)
6-10	205 (1)	22,434 (1)	22,639 (1)
11-20	423 (1)	41,165 (1)	41,588 (1)
21 or more	10,948 (37)	1,018,372 (25)	1,029,320 (25)
Missing	15,293 (51)	2,530,655 (62)	2,545,948 (62)
**Number of social contacts aged <18**			
0	12,138 (41)	1,935,681 (48)	1,947,819 (48)
1-5	4,797 (16)	835,491 (21)	840,288 (21)
6-10	696 (2)	106,921 (3)	107,617 (3)
11-20	1,294 (4)	163,396 (4)	164,690 (4)
21 or more	10,978 (37)	1,020,145 (25)	1,031,123 (25)
Missing	12,138 (41)	1,935,681 (48)	1,947,819 (48)
**Number of social contacts aged 18-69**			
0	4,243 (14)	803,071 (20)	807,314 (20)
1-5	6,351 (21)	1,191,642 (29)	1,197,993 (29)
6-10	3,033 (10)	425,739 (10)	428,772 (10)
11-20	5,385 (18)	628,365 (15)	633,750 (15)
21 or more	10,891 (36)	1,012,817 (25)	1,023,708 (25)
Missing	4,243 (14)	803,071 (20)	807,314 (20)
**Number of social contacts aged >=70**			
0	12,138 (41)	1,935,681 (48)	1,947,819 (48)
1-5	4,797 (16)	835,491 (21)	840,288 (21)
6-10	696 (2)	106,921 (3)	107,617 (3)
11-20	1,294 (4)	163,396 (4)	164,690 (4)
21 or more	10,978 (37)	1,020,145 (25)	1,031,123 (25)
Missing	12,138 (41)	1,935,681 (48)	1,947,819 (48)
**Outside socialising times**			
None	409 (1)	80,290 (2)	80,699 (2)
Once	345 (1)	52,733 (1)	53,078 (1)
Twice	208 (1)	28,400 (1)	28,608 (1)
Three times	128 (0)	14,056 (0)	14,184 (0)
Four times	54 (0)	6,834 (0)	6,888 (0)
Five times	52 (0)	4,194 (0)	4,246 (0)
Six times	19 (0)	1,468 (0)	1,487 (0)
Seven times or more	43 (0)	4,718 (0)	4,761 (0)
Missing	28,645 (96)	3,868,941 (95)	3,897,586 (95)
**Outside shopping only times**			
None	260 (1)	32,514 (1)	32,774 (1)
Once	297 (1)	47,098 (1)	47,395 (1)
Twice	291 (1)	48,764 (1)	49,055 (1)
Three times	180 (1)	30,207 (1)	30,387 (1)
Four times	84 (0)	13,948 (0)	14,032 (0)
Five times	56 (0)	7,835 (0)	7,891 (0)
Six times	14 (0)	2,663 (0)	2,677 (0)
Seven times or more	76 (0)	9,669 (0)	9,745 (0)
Missing	28,645 (96)	3,868,936 (95)	3,897,581 (95)
**Time spent shopping or socializing outside**			
None	3,180 (11)	513,784 (13)	516,964 (13)
Once	3,687 (12)	634,651 (16)	638,338 (16)
Twice	3,719 (12)	602,006 (15)	605,725 (15)
Three times	2,236 (7)	356,644 (9)	358,880 (9)
Four times	1,133 (4)	180,386 (4)	181,519 (4)

Characteristic†	Positive, n (%) or median (IQR)	Negative, n (%) or median (IQR)	Total, n (%) or median (IQR)
Five times	737 (2)	111,370 (3)	112,107 (3)
Six times	293 (1)	44,966 (1)	45,259 (1)
Seven times or more	1,196 (4)	177,612 (4)	178,808 (4)
Missing	13,722 (46)	1,440,215 (35)	1,453,937 (36)
**Hours spent in other's homes**			
None	11,597 (39)	1,954,027 (48)	1,965,624 (48)
Once	2,619 (9)	397,279 (10)	399,898 (10)
Twice	830 (3)	125,436 (3)	126,266 (3)
Three	356 (1)	49,845 (1)	50,201 (1)
Four	175 (1)	23,312 (1)	23,487 (1)
Five	129 (0)	19,331 (0)	19,460 (0)
Six	49 (0)	6,566 (0)	6,615 (0)
Seven or more	261 (1)	34,453 (1)	34,714 (1)
Missing	13,887 (46)	1,451,385 (36)	1,465,272 (36)
**Hours others spent in own home**			
None	10,753 (36)	1,780,000 (44)	1,790,753 (44)
Once	2,906 (10)	490,148 (12)	493,054 (12)
Twice	1,139 (4)	171,857 (4)	172,996 (4)
Three times	502 (2)	69,515 (2)	70,017 (2)
Four times	201 (1)	32,278 (1)	32,479 (1)
Five times	180 (1)	23,641 (1)	23,821 (1)
Six times	60 (0)	7,818 (0)	7,878 (0)
Seven times or more	254 (1)	32,741 (1)	32,995 (1)
Missing	13,908 (47)	1,453,636 (36)	1,467,544 (36)
**Face coverings (work/school)**			
Not going to work/school	1,491 (5)	854,693 (21)	856,184 (21)
Never	1,190 (4)	413,563 (10)	414,753 (10)
Yes, sometimes	433 (1)	111,707 (3)	112,140 (3)
Yes, always	434 (1)	124,250 (3)	124,684 (3)
Face already covered	26,355 (88)	2,557,421 (63)	2,583,776 (63)
Missing	1,491 (5)	854,693 (21)	856,184 (21)
**Face coverings (other situations)**			
Yes, always	113 (0)	49,684 (1)	49,797 (1)
Yes, sometimes	3,156 (11)	1,376,289 (34)	1,379,445 (34)
Face already covered	120 (0)	39,674 (1)	39,794 (1)
Not going to enclosed public spaces	181 (1)	49,240 (1)	49,421 (1)
Never	26,333 (88)	2,546,747 (63)	2,573,080 (63)
Missing	113 (0)	49,684 (1)	49,797 (1)

† All characteristics except hours spent with someone else in one's own home per day relate to the past 7 days.

Note: To distinguish between physical and social contacts participants were asked either "how many adults not living in your home have you had physical contact with (e.g. handshake, personal care), including with PPE if you wear it?" or "how many adults not living in your home have you had direct, but not physical contact with in person, e.g. with social distancing only?"

Supplementary Table 2: Count in each fortnight, including number not included in core model

Fortnight	Positive visits, n (%)	Negative visits, n (%)	Total, n (%)	Negative visits excluded from core models*, n (% of negatives)
19Jul20-01Aug20	27 (0·1)	32,157 (99·9)	32,184 (100)	4,074 (12.7)
02Aug20-15Aug20	22 (0·1)	43,073 (99·9)	43,095 (100)	86,72 (20·1)
16Aug20-29Aug20	41 (0·1)	57,895 (99·9)	57,936 (100)	0 (0·0)
30Aug20-12Sep20	111 (0·1)	76,276 (99·9)	76,387 (100)	0 (0·0)
13Sep20-26Sep20	320 (0·3)	116,467 (99·7)	116,787 (100)	0 (0·0)
27Sep20-10Oct20	1,090 (0·6)	171,298 (99·4)	172,388 (100)	0 (0·0)
11Oct20-24Oct20	1,995 (1·0)	194,123 (99·0)	196,118 (100)	0 (0·0)
25Oct20-07Nov20	2,109 (1·2)	169,735 (98.8)	171,844 (100)	0 (0·0)
08Nov20-21Nov20	2,316 (1·2)	192,715 (98.8)	195,031 (100)	0 (0·0)
22Nov20-05Dec20	1,874 (1·0)	192,534 (99·0)	194,408 (100)	0 (0·0)
06Dec20-19Dec20	2,286 (1·2)	190,313 (98.8)	192,599 (100)	0 (0·0)
20Dec20-02Jan21	2,710 (1·9)	136,703 (98.1)	139,413 (100)	0 (0·0)
03Jan21-16Jan21	3,891 (1·9)	198,116 (98.1)	202,007 (100)	0 (0·0)
17Jan21-30Jan21	3,275 (1·7)	194,157 (98.3)	197,432 (100)	0 (0·0)
31Jan21-13Feb21	2,171 (1·0)	205,148 (99·0)	207,319 (100)	0 (0·0)
14Feb21-27Feb21	1,058 (0·5)	196,410 (99·5)	197,468 (100)	0 (0·0)
28Feb21-13Mar21	621 (0·3)	193,549 (99·7)	194,170 (100)	0 (0·0)
14Mar21-27Mar21	475 (0·3)	173,734 (99·7)	174,209 (100)	0 (0·0)
28Mar21-10Apr21	364 (0·2)	169,692 (99·8)	170,056 (100)	0 (0·0)
11Apr21-24Apr21	189 (0·1)	164,958 (99·9)	165,147 (100)	0 (0·0)
25Apr21-08May21	123 (0·1)	172,931 (99·9)	173,054 (100)	0 (0·0)
09May21-22May21	137 (0·1)	164,249 (99·9)	164,386 (100)	0 (0·0)
23May21-05Jun21	240 (0·1)	160,888 (99·9)	161,128 (100)	0 (0·0)
06Jun21-19Jun21	309 (0·2)	167,862 (99·8)	168,171 (100)	0 (0·0)
20Jun21-03Jul21	675 (0·4)	159,246 (99·6)	159,921 (100)	0 (0·0)
04Jul21-17Jul21	1,474 (0·9)	167,405 (99·1)	168,879 (100)	0 (0·0)

* Negative visits were excluded in the two earliest fortnights due to perfect prediction

Supplementary Table 3: Odds ratios and p-values for the effect of testing positive in the survey >120 days before current visit

Fortnight	Odds ratio (95% p-value)	P-values
31Jan21-13Feb21	0.26 (0.09, 1.46)	0.155
14Feb21-27Feb21	0.64 (0.24, 1.71)	0.371
28Feb21-13Mar21	1.10 (0.52, 2.32)	0.807
14Mar21-27Mar21	1.26 (0.67, 2.38)	0.470
28Mar21-10Apr21	0.66 (0.27, 1.60)	0.353
11Apr21-24Apr21	1.07 (0.44, 2.61)	0.886
25Apr21-08May21	1.36 (0.55, 3.35)	0.505
09May21-22May21	1.27 (0.59, 2.72)	0.546
23May21-05Jun21	1.12 (0.62, 2.01)	0.703
06Jun21-19Jun21	1.07 (0.66, 1.76)	0.774
20Jun21-03Jul21	0.51 (0.31,0.83)	0.006
04Jul21-17Jul21	0.33 (0.23, 0.49)	<0.001

Note: Model adjusted for core variables

Supplementary Table 4: Summary of individuals IMD components with combined index

	Correlation with combined index	Proportion of score*
**Combined**	1	
**Income**	0·93	22·5
**Employment**	0·90	22·5
**Education**	0·78	13·5
**Health**	0·81	13·5
**Crime**	0·68	9·3
**Housing**	0·18	9·3
**Indoors**	0·35	6·2
**Outdoors**	0·25	3·1
**Living environment (combination of "indoors" and "outdoors")**	0·41	9·3

*Taken from https://www.gov.uk/government/statistics/english-indices-of-deprivation-2019

Supplementary Table 5: Summary of p-values in 28-day periods for effects which occur in 2 or more consecutive fortnights

	Number of occurrences, n (%) [N=45]
**Not detected in 28-day periods**	1 (2)
**Same detection date**	14 (31)
**Detected later in 28-day periods**	25 (56)
**Detected earlier in 28-day periods**	5 (11)

Note: The effect not detected in 28-day periods was work sector IT in the fortnight 20th June-3rd July. Variables which would have been detected earlier in 28-day periods (number of days earlier in brackets) are as follows: contact with hospital (14 days), work in a patient facing healthcare role (14 days), education deprivation index (14 days), sector health care (42 days), study visit frequency (56 days).

Additional to these earlier detections, for eight variables in ten 28-day periods, the effect had $p<0{\cdot}05$ in a 28-day period but $p\geq0{\cdot}05$ in both the nested fortnights. Of these ten instances, two were significant in related variables within the nested fortnights[a], four were identified in one of the two fortnights directly prior[b], one was picked up in the fortnight directly after[c], and three were not found in any fortnight directly before or after[d].

[a]Ever smoked regularly in the monthly period 11Oct20-07Nov20 (p=0·041). During the fortnights spanning 11Oct20-07Nov20, current smoking status was consistently identified. Impact of long-term health conditions was identified in the 28-days 31Jan21-27Feb21 (p=0·035), where it was marginally significant in the nested fortnight 31Jan21-13Feb21 (p=0·059). Both any long-term health conditions, and disability were flagged as significant in this fortnight.

[b]Any long-term health conditions in 8Nov20-5Dec20; Indoors deprivation index (16Aug20-12Sep20); Sector food production in 31Jan21-27Feb21; Travel abroad (08Nov20-05Dec20; p = 0·047)

[c]Sector finance in 11Oct20-07Nov20

[d]Housing deprivation index in 31Jan21-27Feb21 and 28Mar21-24Apr21 (but this effect did not have an effect after adjusting for overall deprivation index); sector finance in 31Jan21-27Feb21

Supplementary Figures

Supplementary Figure 1: Number of participants followed-up per month of study period

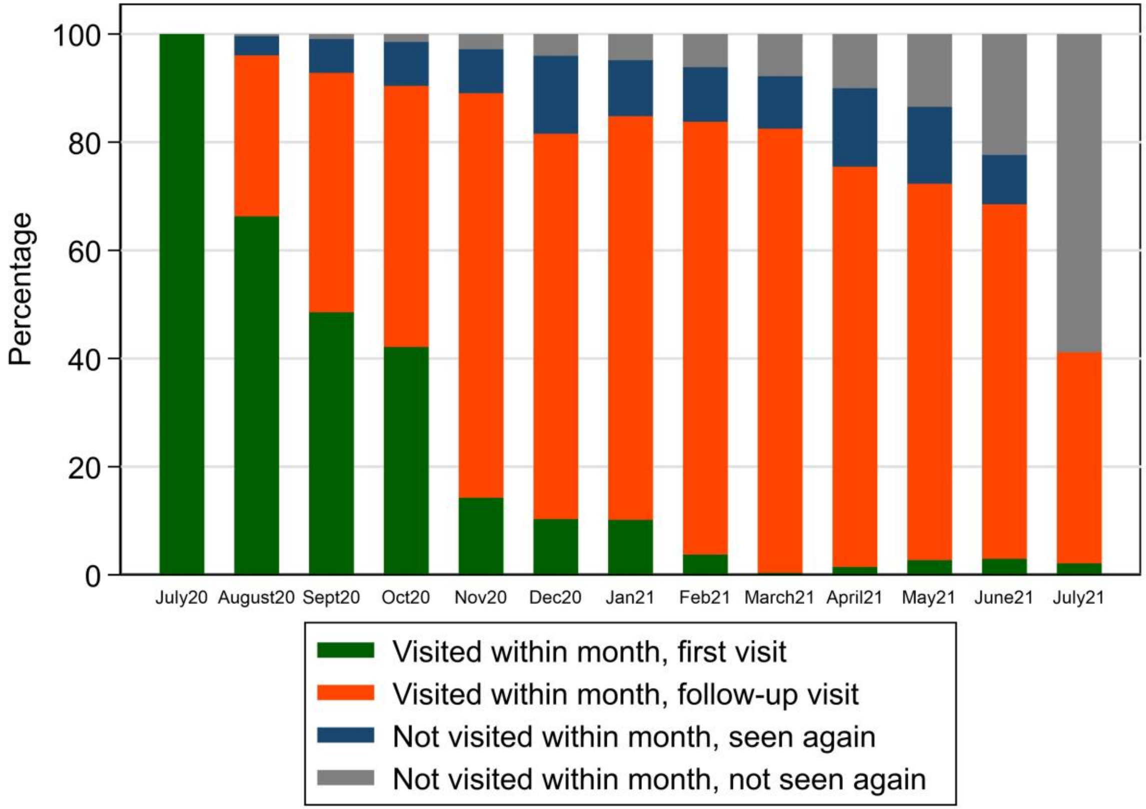

Note: The denominator for each month is the number of visits within that month, plus any participants with a visits in a previous month. By this definition, July 2020 includes only those participants visited in July 2020 and, all visits in July 2021 were not seen again. As we only included visits up until 17th July 2021, there are 14 days in the month we did not have chance to observe a follow-up visits. Participants were continuously recruited. Participants initially consented to only an enrolment visit, or to weekly visits for a month or to monthly visits for a year. After completing one year, participants were offered the option to continue in the study.

Supplementary Figure 2: Log odds ratios with 95% confidence intervals for the effect of rural urban classification across the 52 week study period

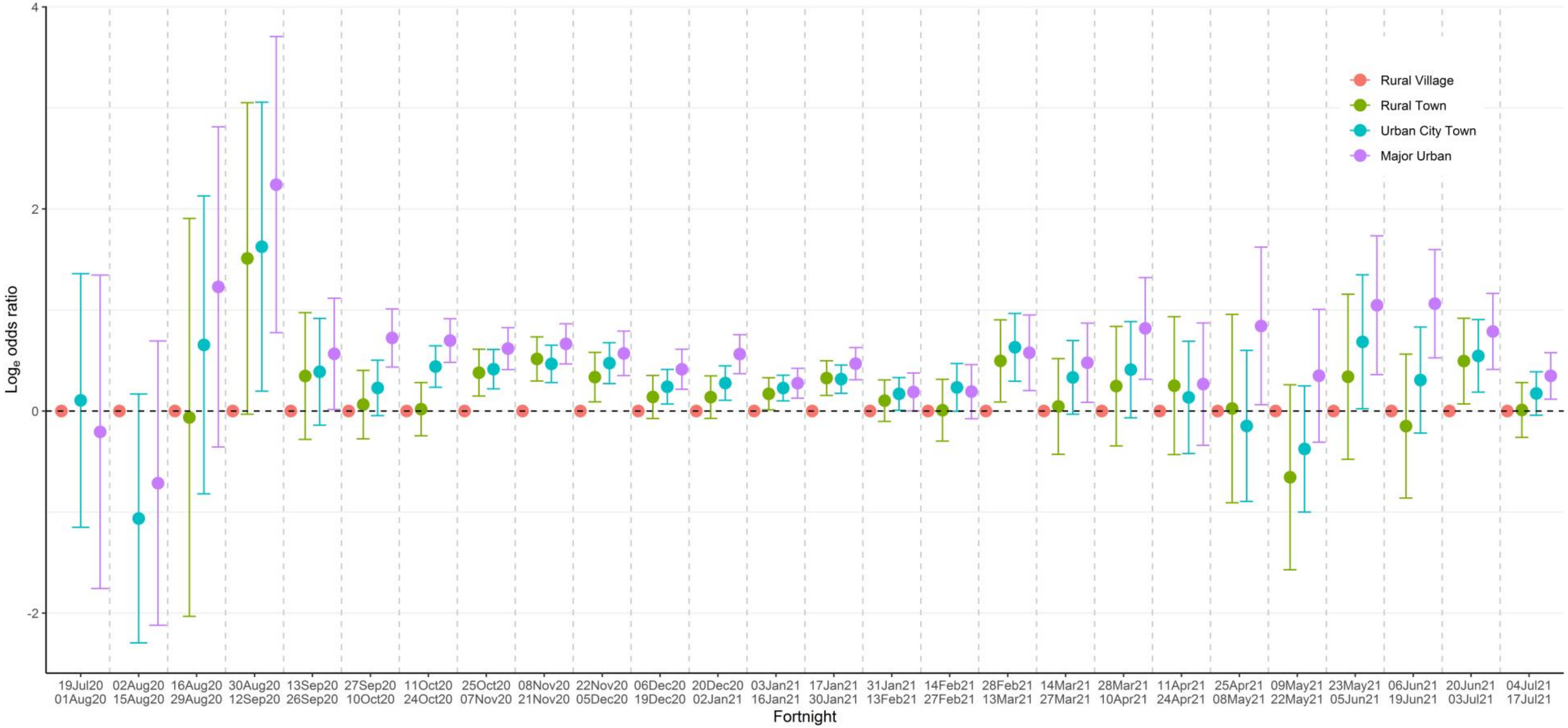

Note: All odds ratios are vs rural village

Supplementary Figure 3: Unadjusted percentage (95% CI) of positive swabs per fortnight (a), and positive swabs split by gene positivity pattern (b) and symptoms (c)

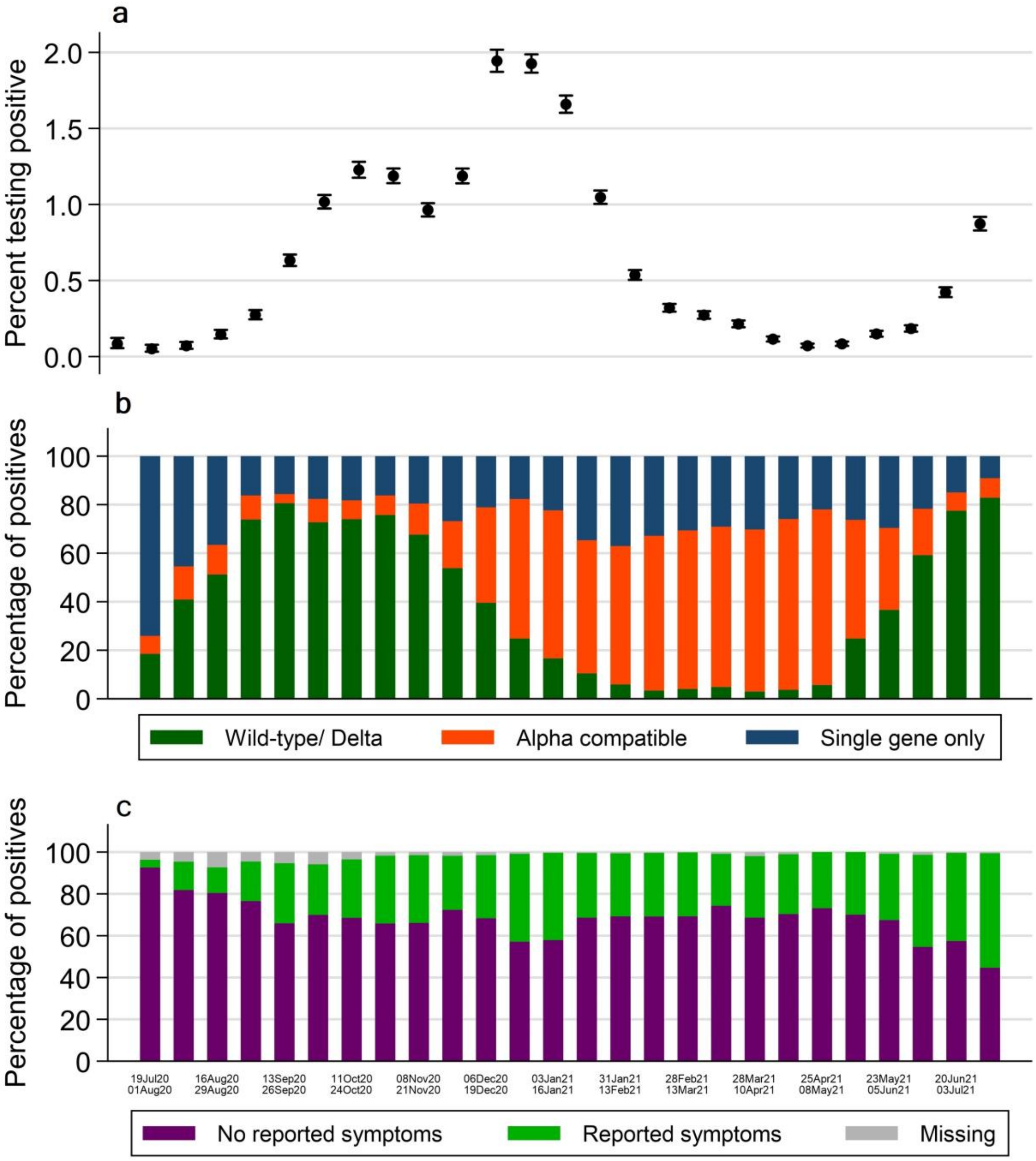

Note: Wild-type/Delta=positive on all three genes (N, S, ORF1ab) or S plus one other gene. Alpha-compatible=positive on N+ORF1ab. Single gene=positive on N or ORF1ab only (S only not considered positive). A separate manuscript investigating associations with symptoms in positives and negatives in detail is available: https://www.medrxiv.org/content/10.1101/2021.08.19.21262231v1.

Supplementary Figure 4: Total number of participants per fortnight

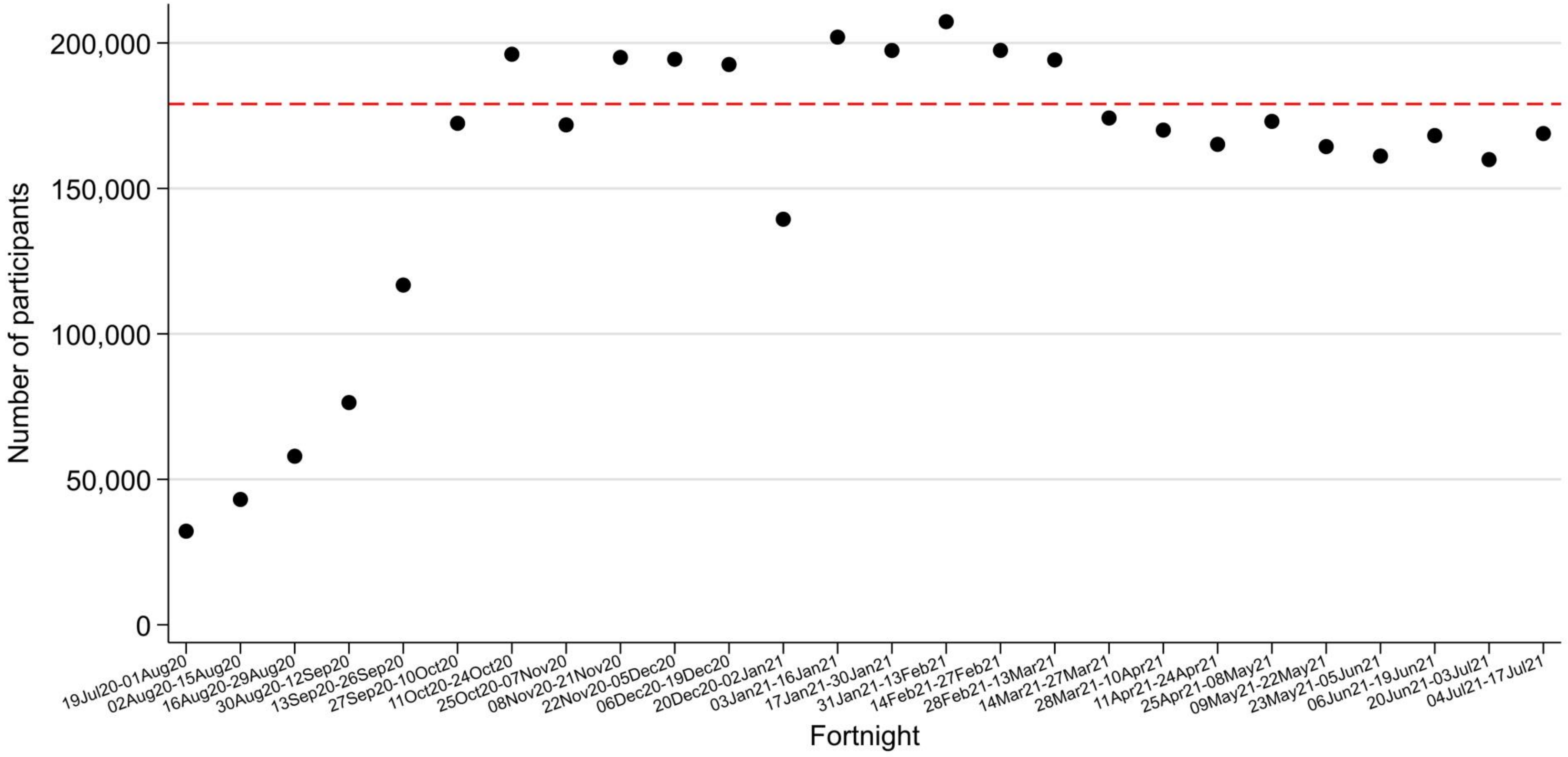

Note: The red dashed line shows the recruitment target of 179,000 swabs from unique participants across the UK from 1st October onwards

Supplementary Figure 5: Summary of odds ratio and p-values for interactions between all of the core variables using fortnights.

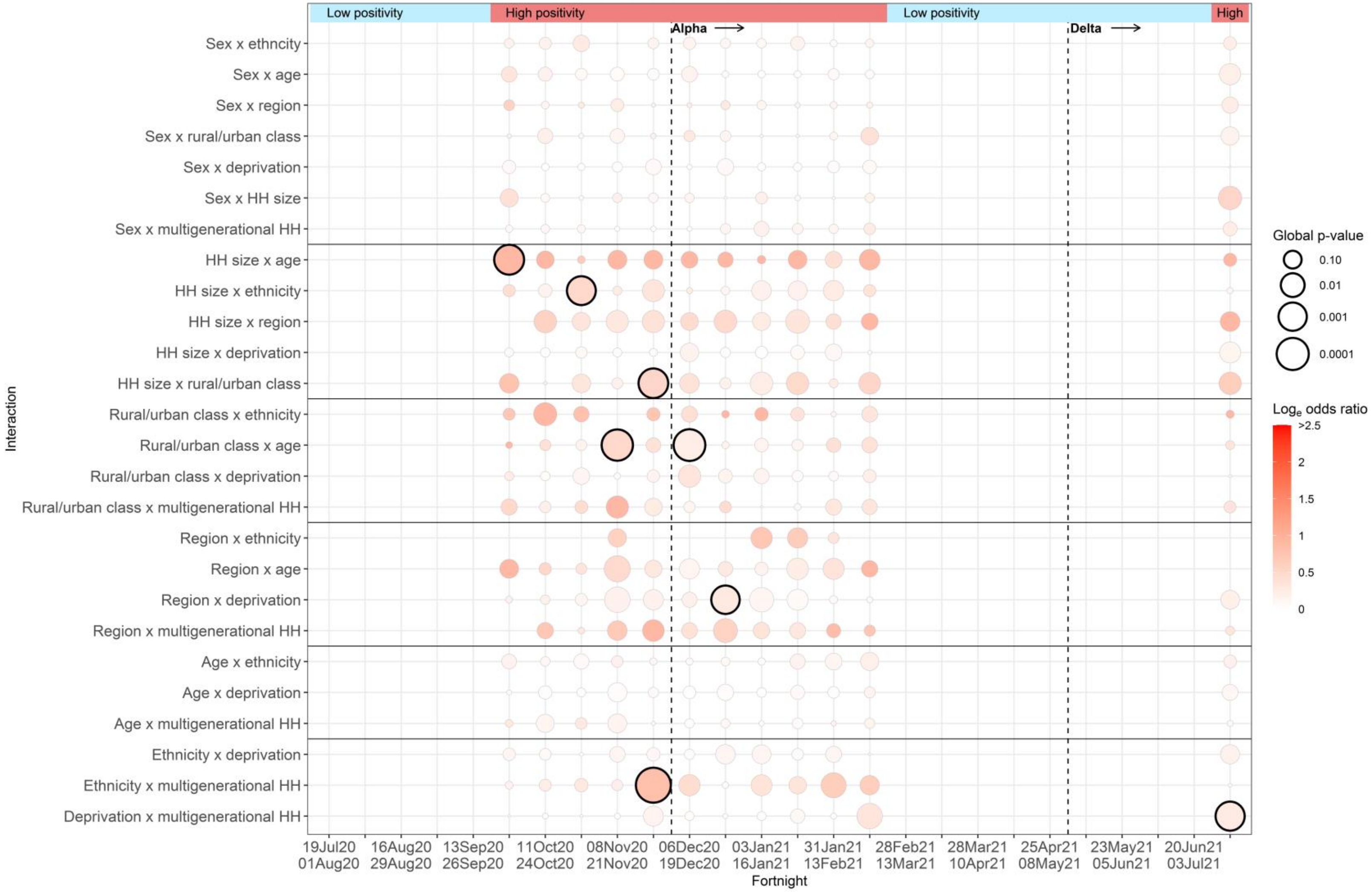

Note: The size of the circles are proportional to -log$_{10}$ of the global heterogeneity p-value for each interaction in each fortnight. The colour of the circles represent the average size of the interaction terms, converted to the odds ratio scale. Black outlined circles had a global heterogeneity p-value<0.001.

Supplementary Figure 6 Overall effects of additional factors from the screening process which were either inconsistently or never associated with positivity, adjusted for the core variables, over the 52 week study period

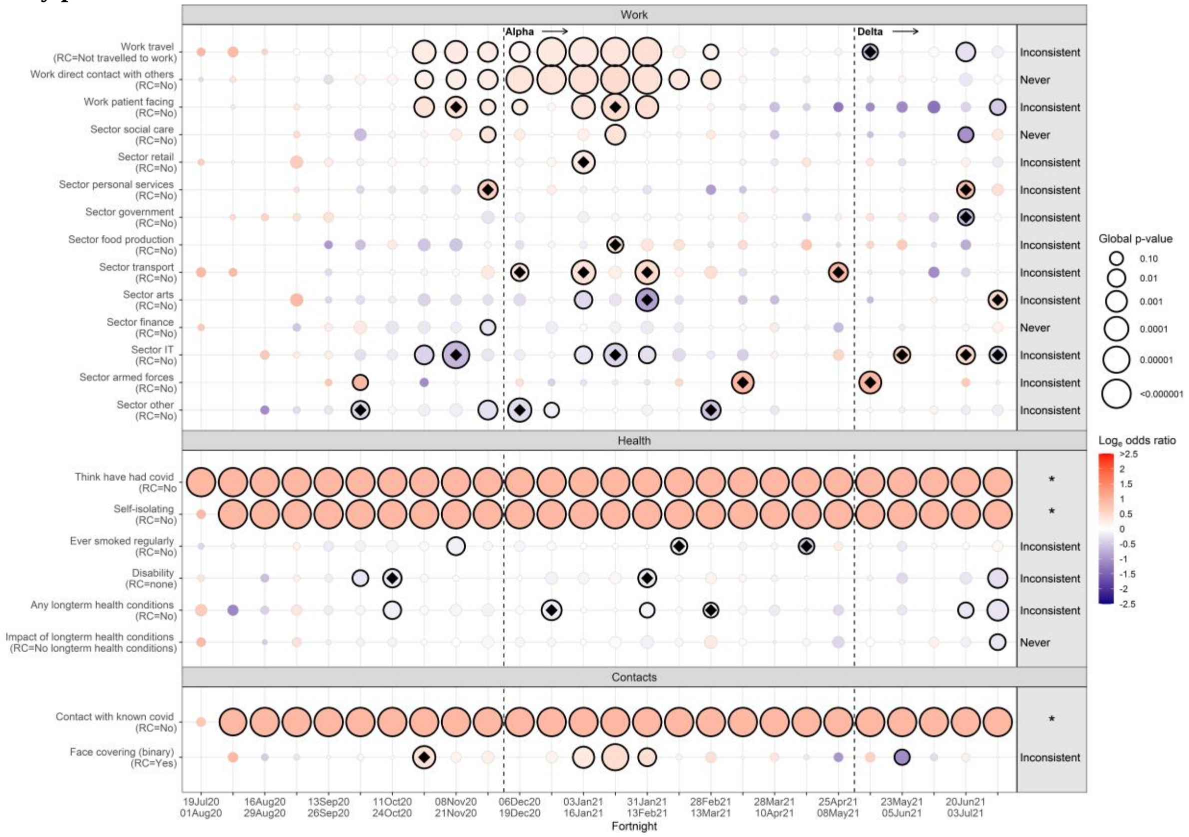

*potential mediators of effects of other factors so not considered in main effects model further

Note: each factor included in addition to the core variables in each fortnight. Black diamonds indicate factors which remain after backwards elimination of all factors with p<0·05 in each fortnight. White squares indicate fortnights where characteristic was not collected by the survey. Categorisation of effect persistence (inconsistent, never) was done after backwards elimination. See **Supplementary Table 1** for variable names and distributions.

Supplementary Figure 7: Effects of individual levels of factors from the screening process, adjusted for the core variables, over the 52 week study period

Health status

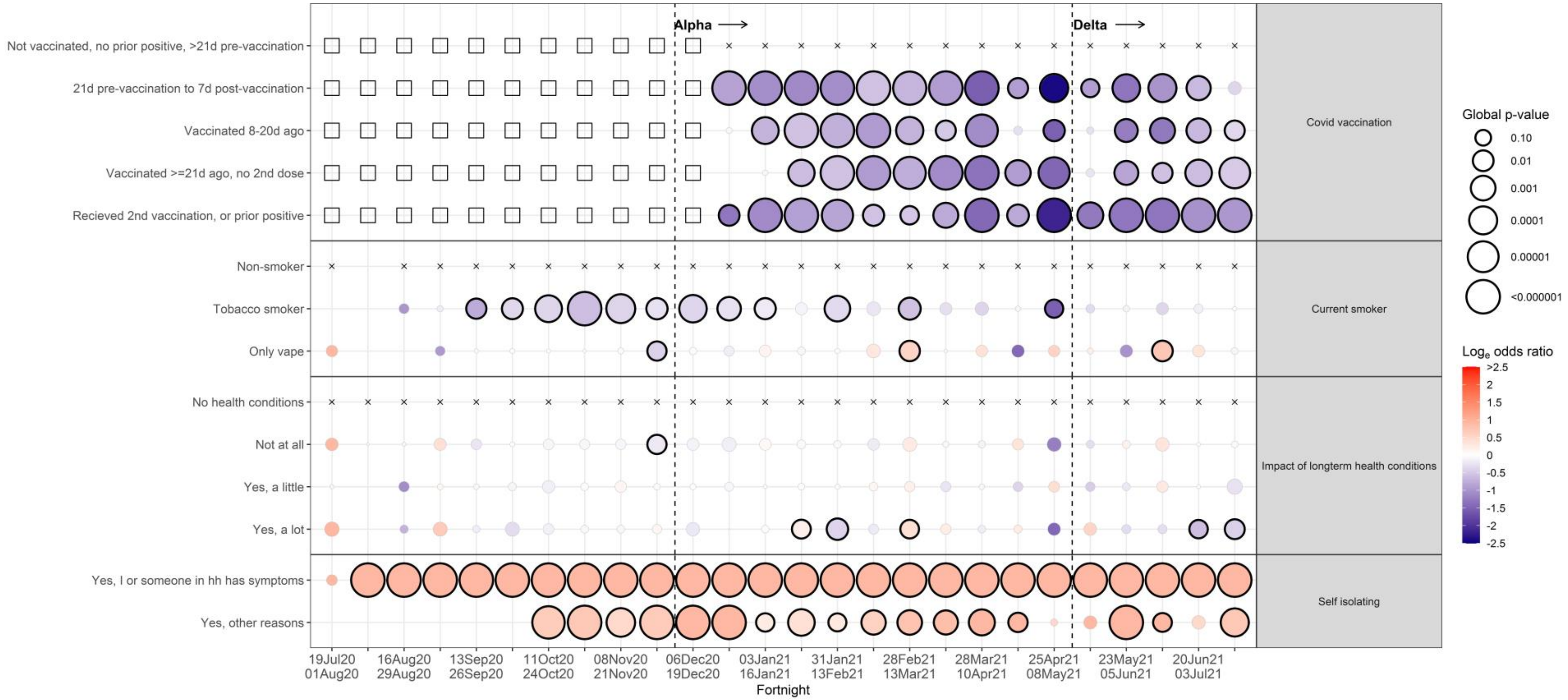

Work and employment

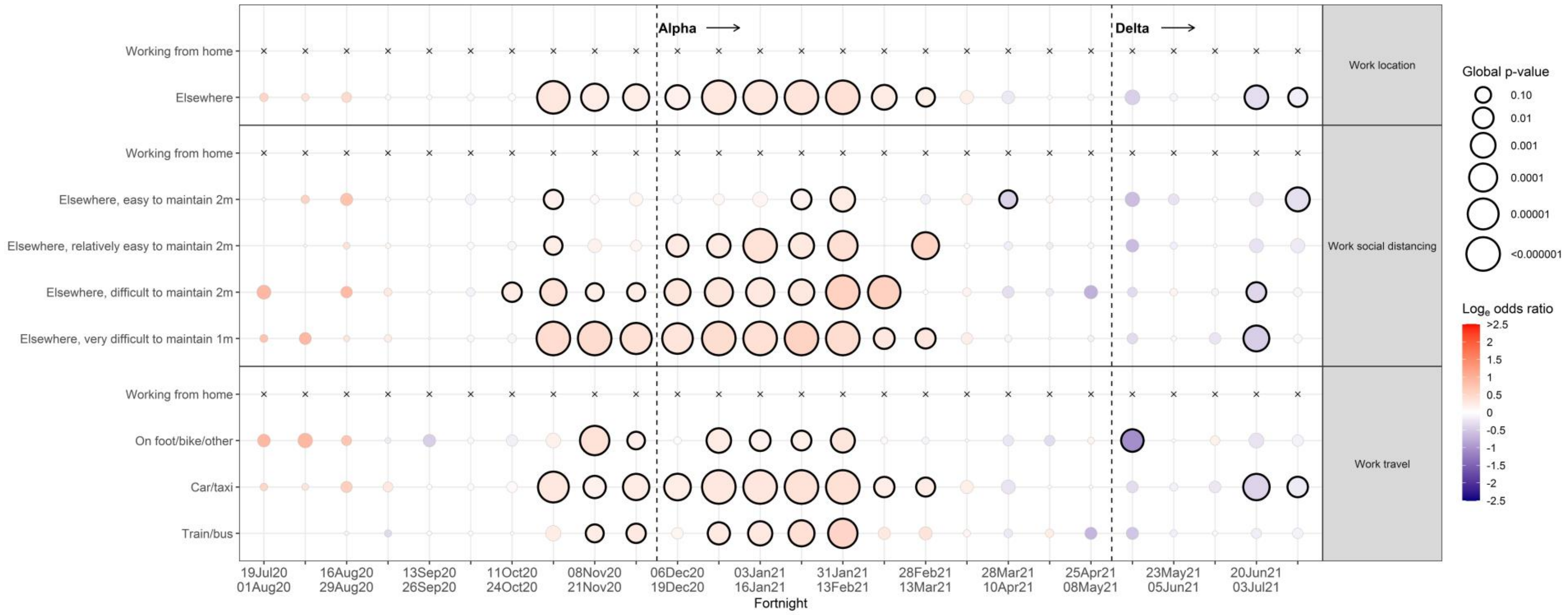

Contacts

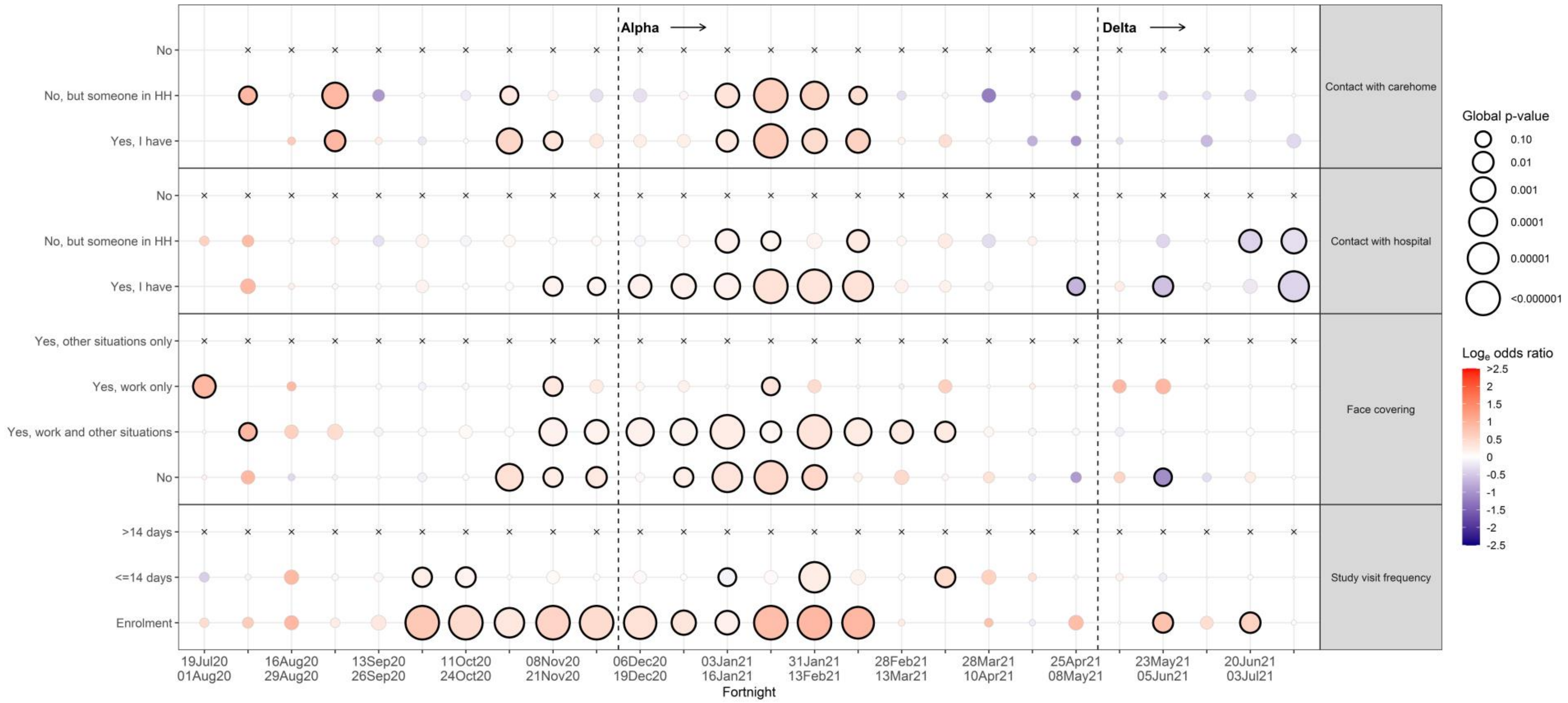

Alpha
Delta
No
No, but someone in HH
Yes, I have
No
No, but someone in HH
Yes, I have
Yes, other situations only
Yes, work only
Yes, work and other situations
No
>14 days
<=14 days
Enrolment
Contact with carehome
Contact with hospital
Face covering
Study visit frequency
19Jul20 01Aug20
16Aug20 29Aug20
13Sep20 26Sep20
11Oct20 24Oct20
08Nov20 21Nov20
06Dec20 19Dec20
03Jan21 16Jan21
31Jan21 13Feb21
28Feb21 13Mar21
28Mar21 10Apr21
25Apr21 08May21
23May21 05Jun21
20Jun21 03Jul21
Fortnight
Global p-value
0.10
0.01
0.001
0.0001
0.00001
<0.000001
Log$_e$ odds ratio
>2.5
2
1.5
1
0.5
0
-0.5
-1
-1.5
-2
-2.5

Supplementary Figure 8: Global hetergeneity p-values per factor from the screening process for household and living enviroment characteristics

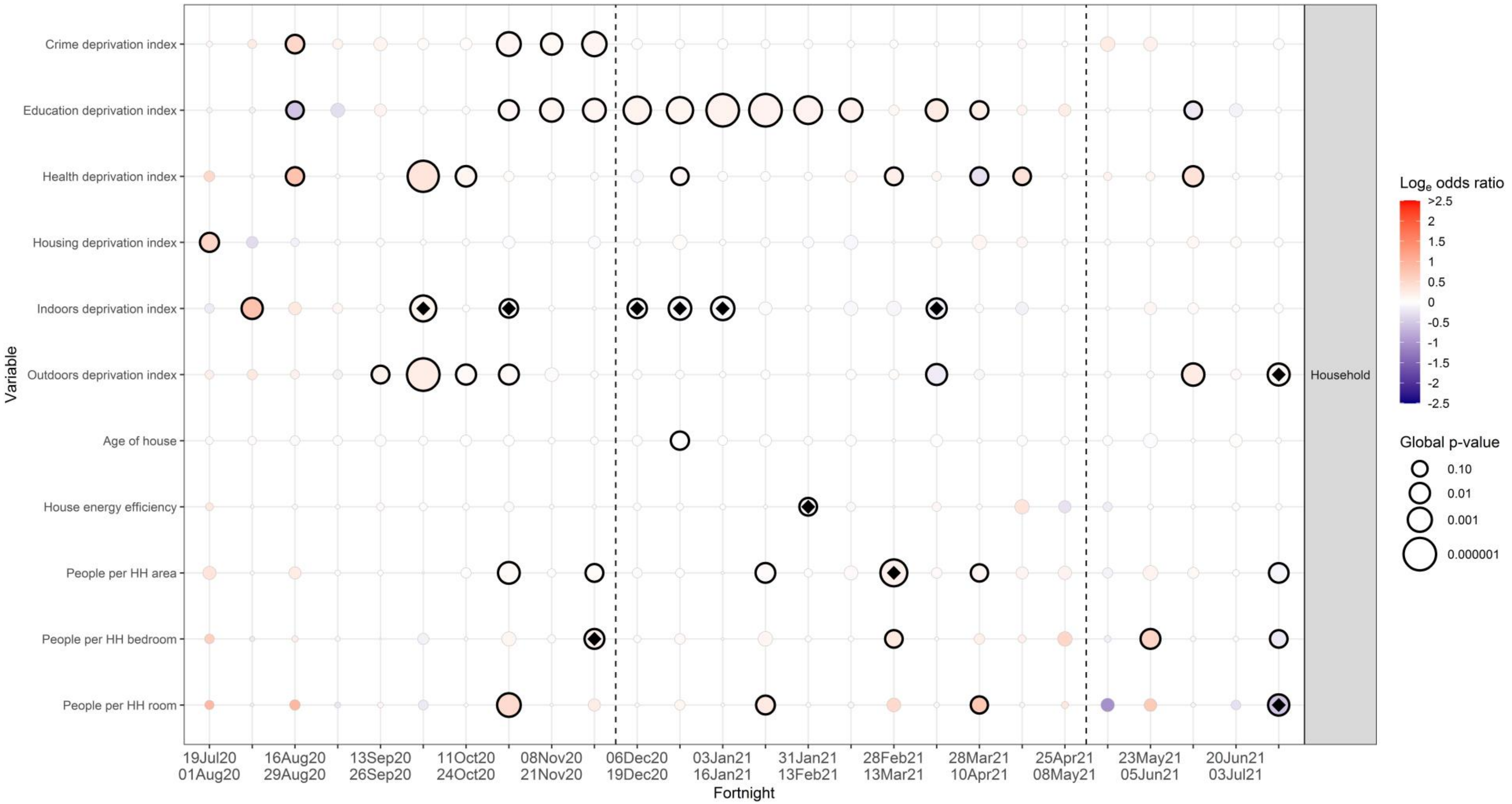

Note: each factor included in addition to the core variables in each fortnight. Black diamonds indicate factors which remain after backswards elimination of all factors with p<0·05 in each fortnight. Categorisation of effect persistence (inconsistent, never) was done after backwards elimination. See **Supplementary Table 1** for variable names and distributions.

Supplementary Figure 9: Overall effects of additional factors from the behavioural screening process which were either inconsistently or never associated with positivity, adjusted for the core variables, over the 52 week study period

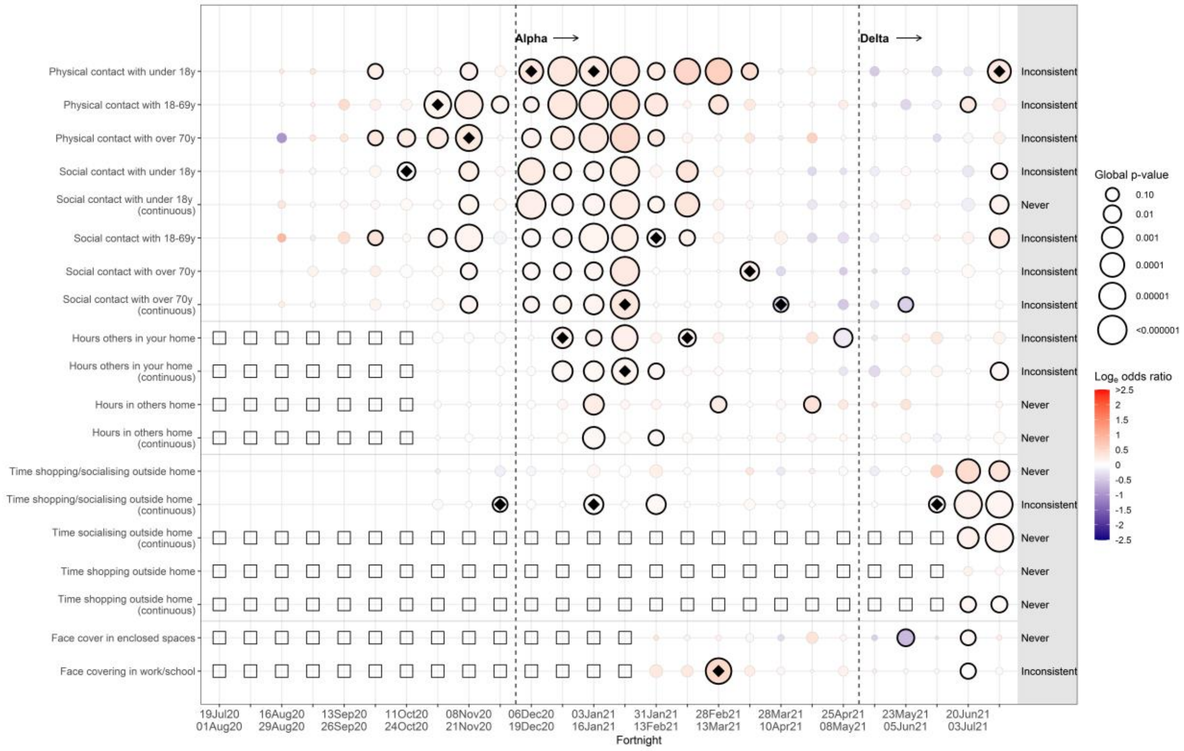

Note: each factor included in addition to the core variables in each fortnight. Black diamonds indicate factors which remain after backswards elimination of all factors with p<0·05 in each fortnight. White squares indicate fortnights where characteristic was not collected by the survey. Categorisation of effect persistence (inconsistent, never) was done after backwards elimination. See **Supplementary Table 1** for variable names and distributions.

Supplementary Figure 10: Individual p-values per factor from the screening process for behavioural characteristics

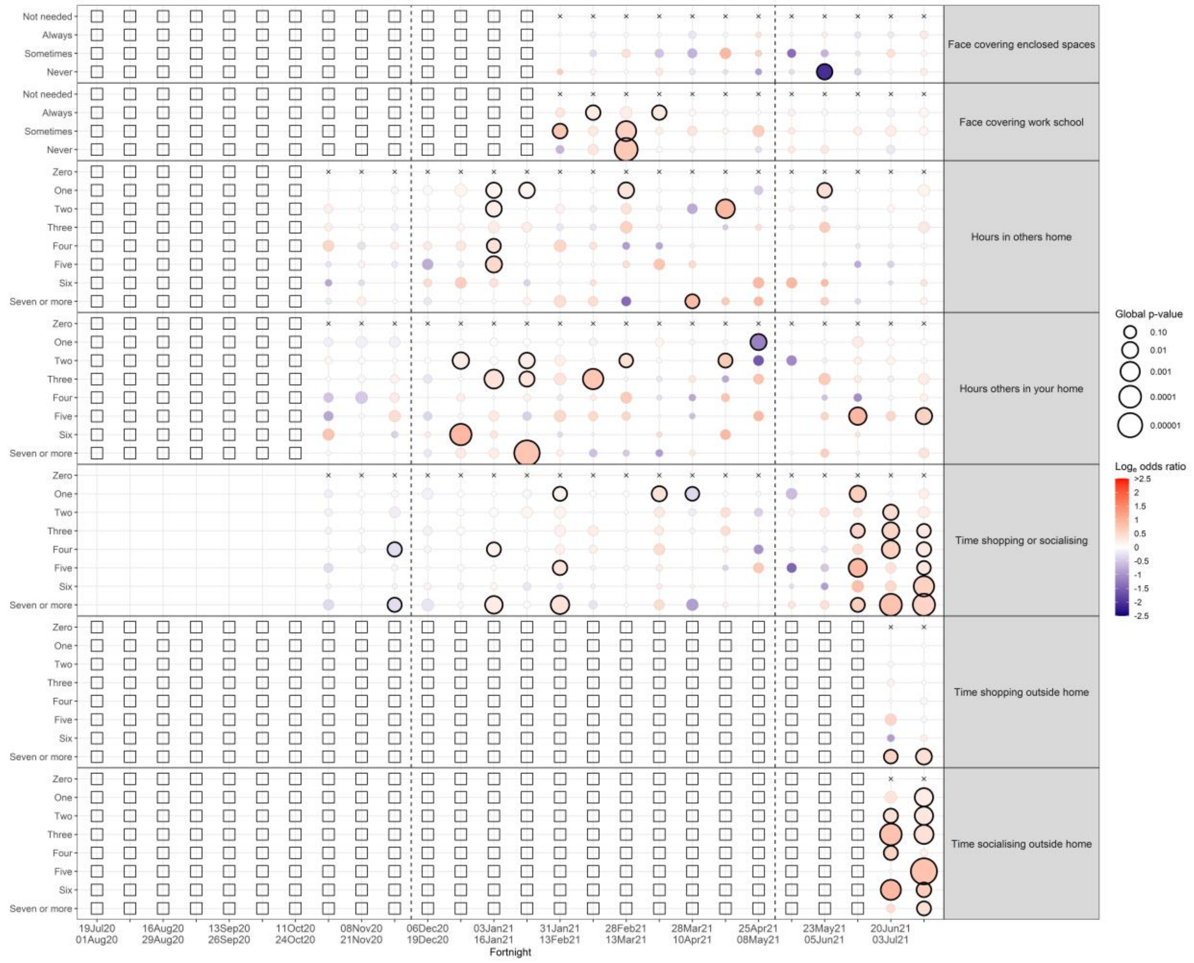

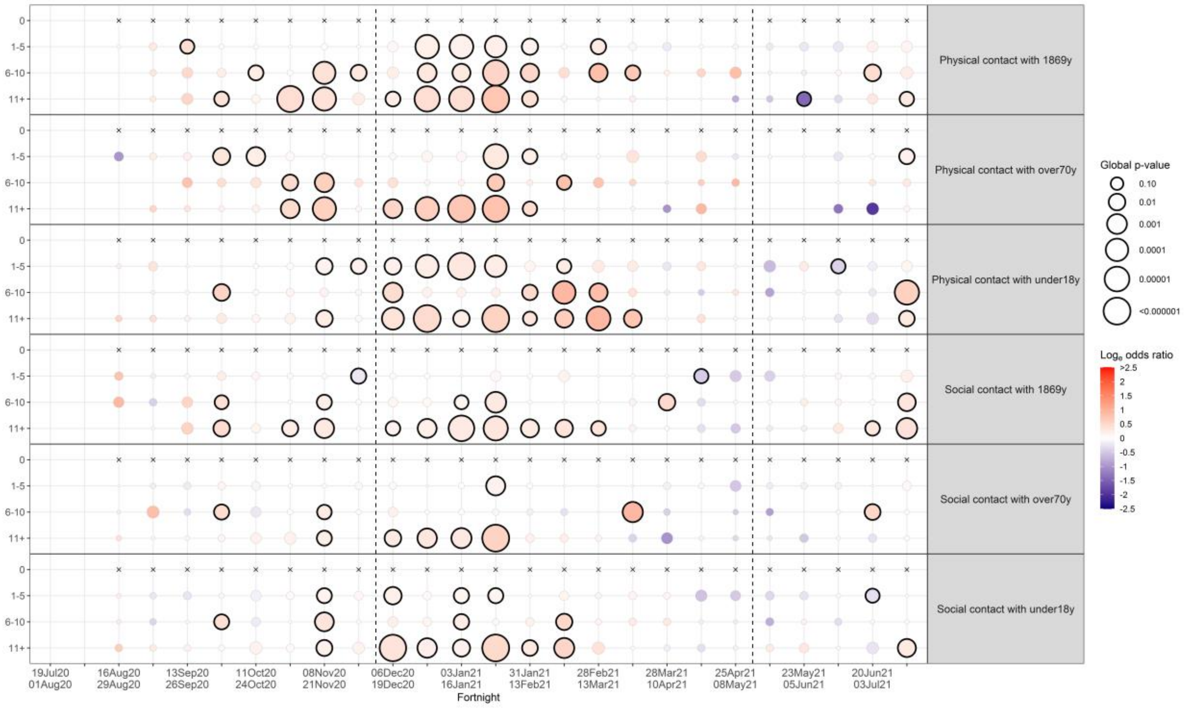

Supplementary Figure 11A: Summary of odds ratios and p-values for the 8 core variables over 28 day periods

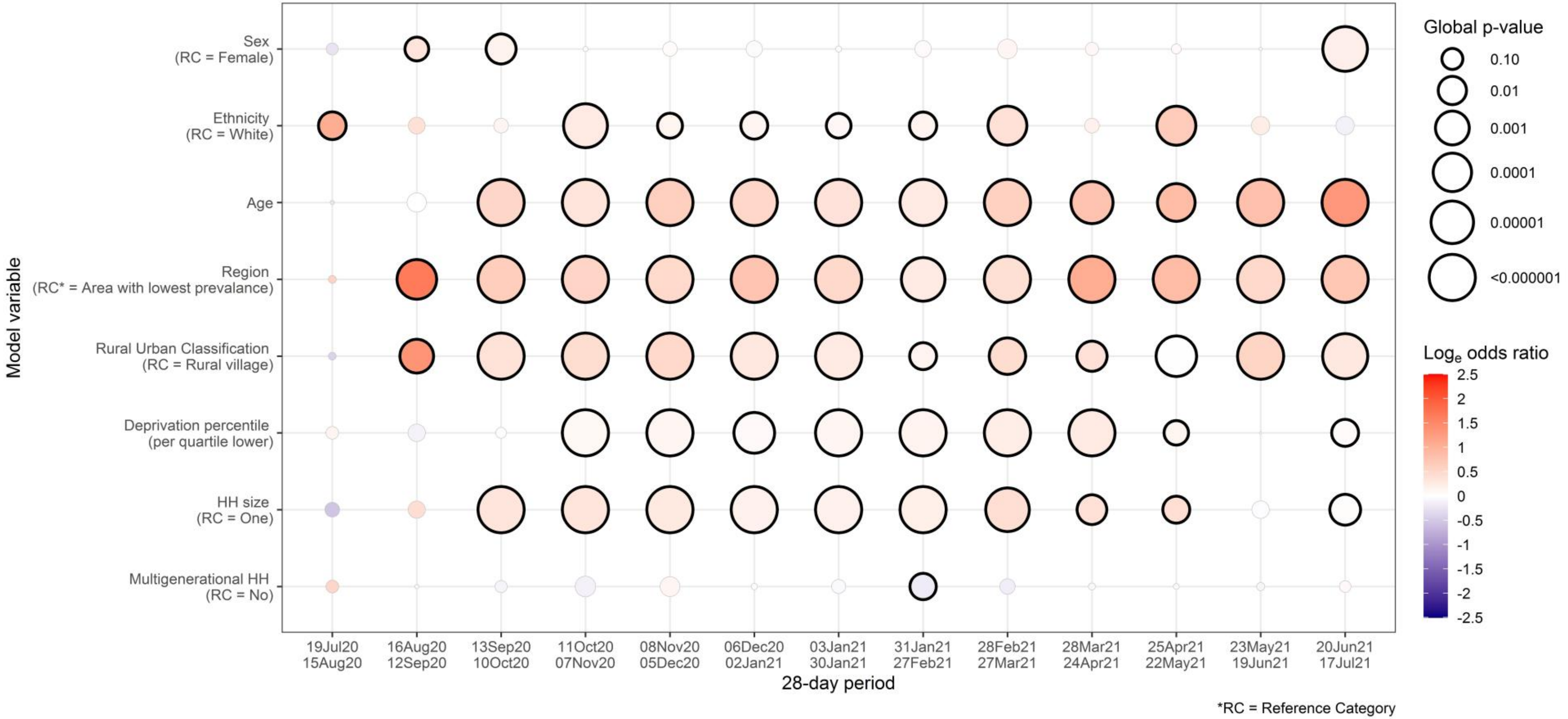

Note: RC=reference category. HH=household size The size of the circles are proportional to -log$_{10}$ of the global heterogeneity p-value for each variable in each 28-day period. Circles with black outlines represent p<0·05. The colour of the circles represent the size of the odds ratio (vs the reference category shown). For categorical variables with >2 levels (region, rural/urban classification, and household size), the reference category was set as the level with the lowest prevalence in each fortnight, and the overall "odds ratio" calculated as: $\exp\left(\frac{\sum\frac{1}{se(\beta_i)}\beta_i}{\sum\frac{1}{se(\beta_i)}}\right)$. As age was included in the model as a restricted natural cubic spline, odds ratios were predicted at ages 10, 25, 40, and 55 vs 70 (reference) years and then combined in the same way.

Supplementary Figure 11B: Summary of odds ratios and p-values for the individual levels of the 8 core variables over 28 day periods

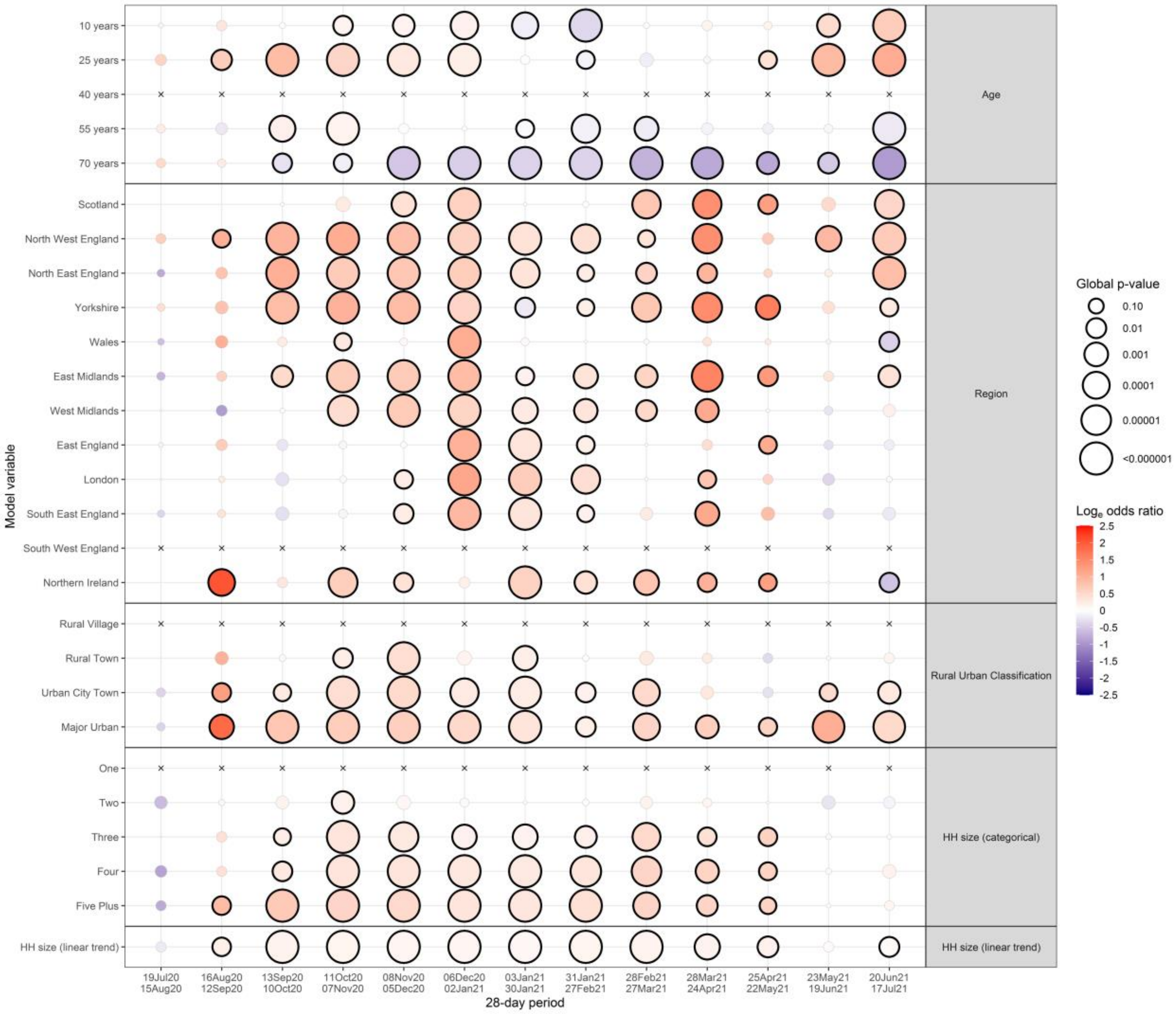

Supplementary Figure 12: Adjusted effect of age (years) on positivity using 28-day periods.

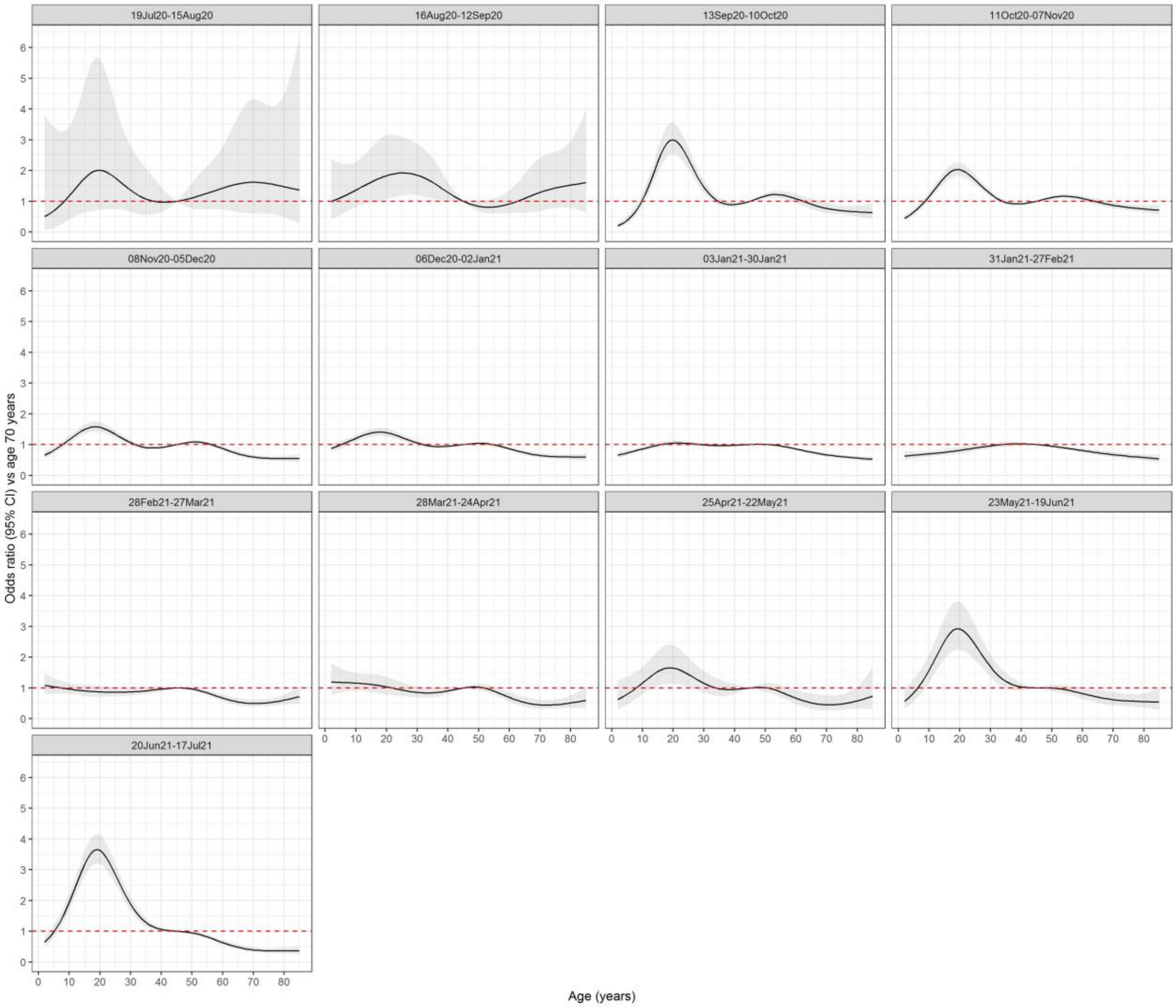

Odds ratios are predicted for each age vs a reference age of 45 years.

Supplementary Figure 13A: Summary of odds ratio and p-values for interactions between all of the core variables for 28 day periods.

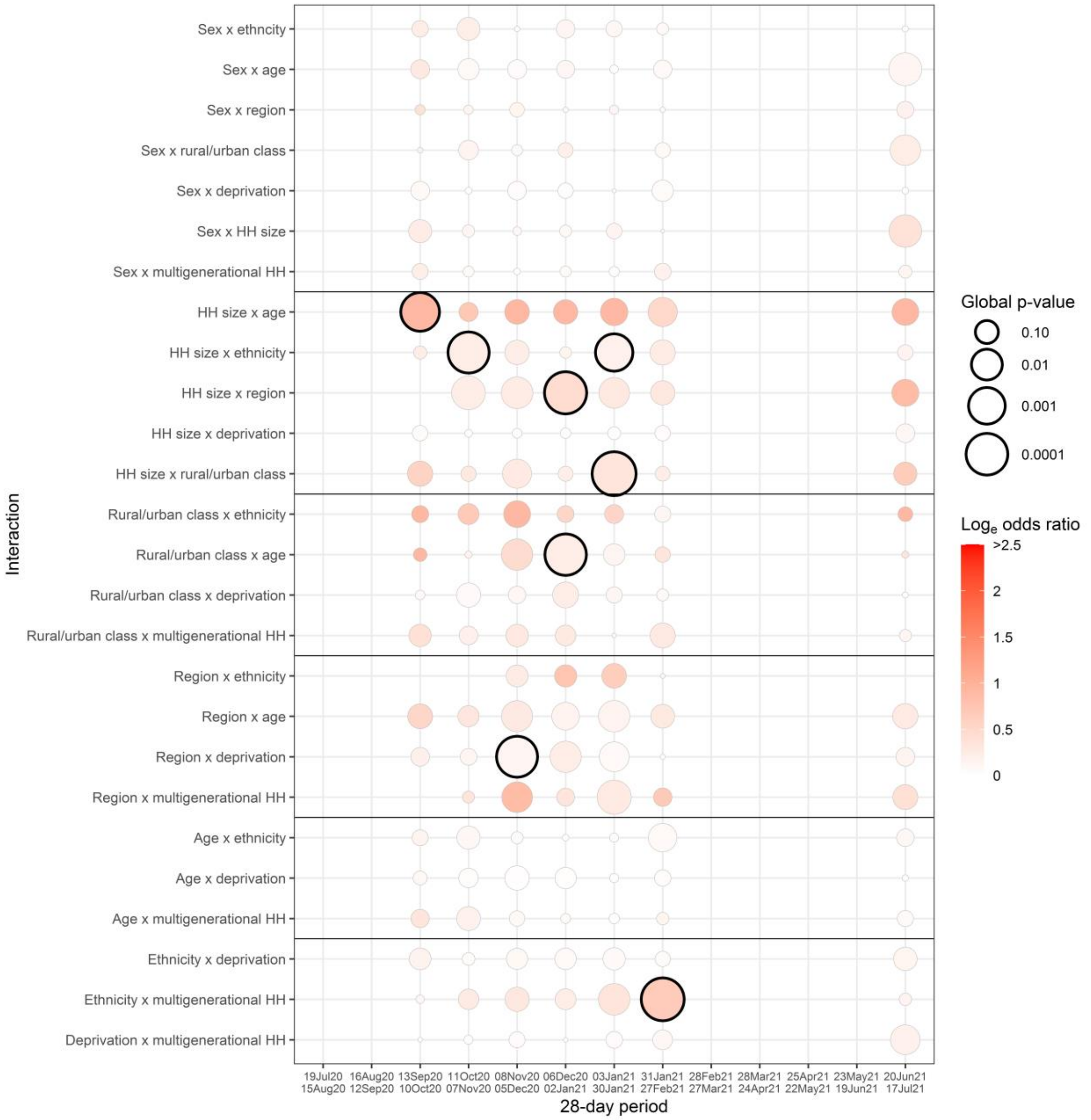

Note: The size of the circles are proportional to -log$_{10}$ of the global heterogeneity p-value for each interaction in each fortnight. The colour of the circles represent the size of the odds ratio

Figure 13B: Effect of interaction of age by household size in the 28-day period 13 September to 10th October

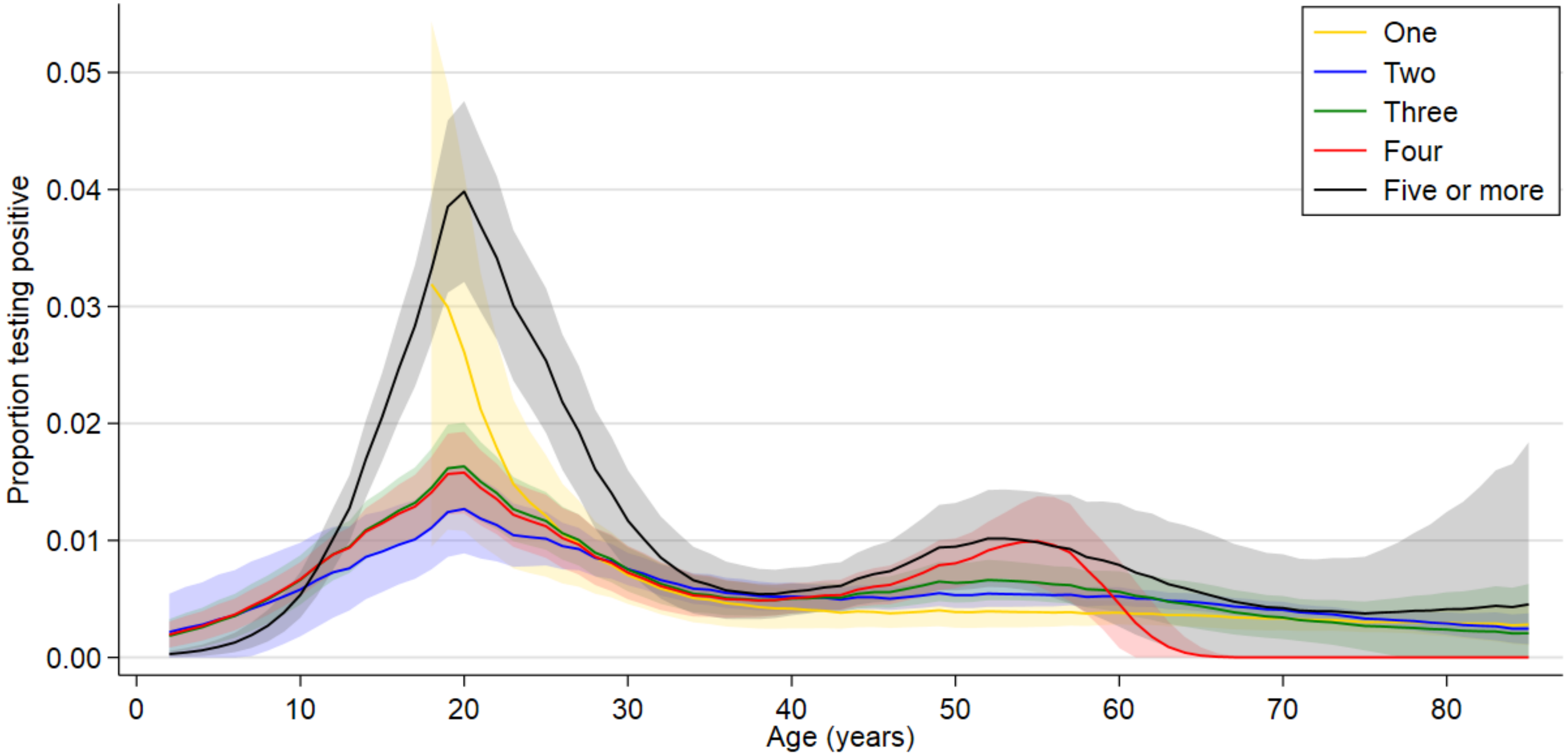

Note: effects marginalised over other variables.

Figure 13C: Effect of interaction of ethnicity by household size in the 28-day period 11th October 2020 to 7th November 2020

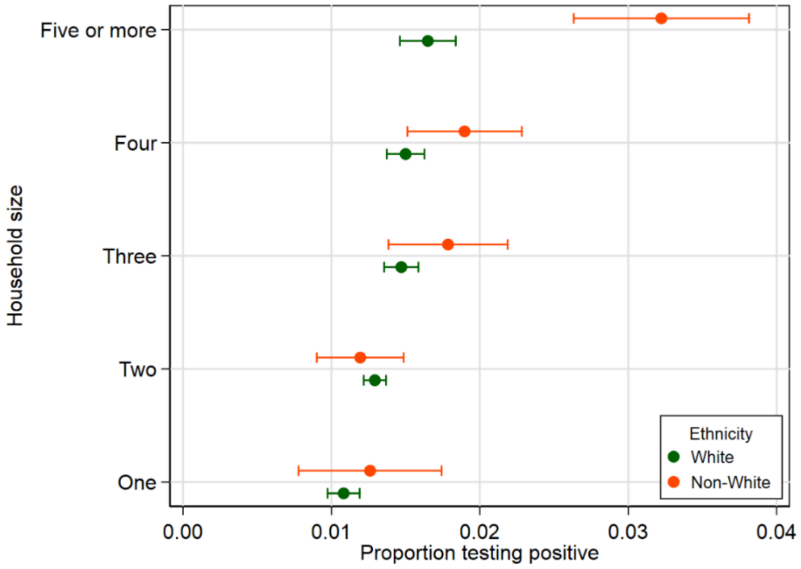

Figure 13D: Effect of interaction of region by deprivation score in the 28-day period 8th November 2020 to 5th December 2020

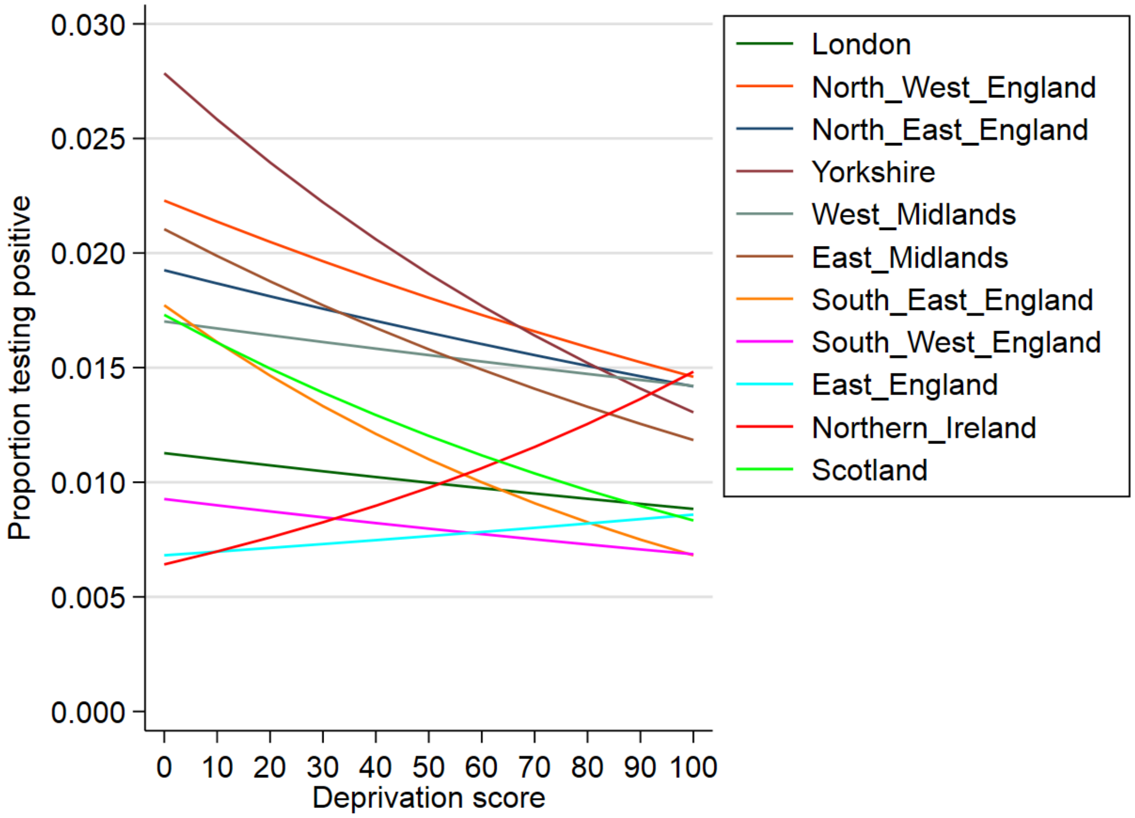

Figure 13E: Effect of interaction of rural urban classification by age in the 28-day period 6th December 2020 to 2nd January 2021

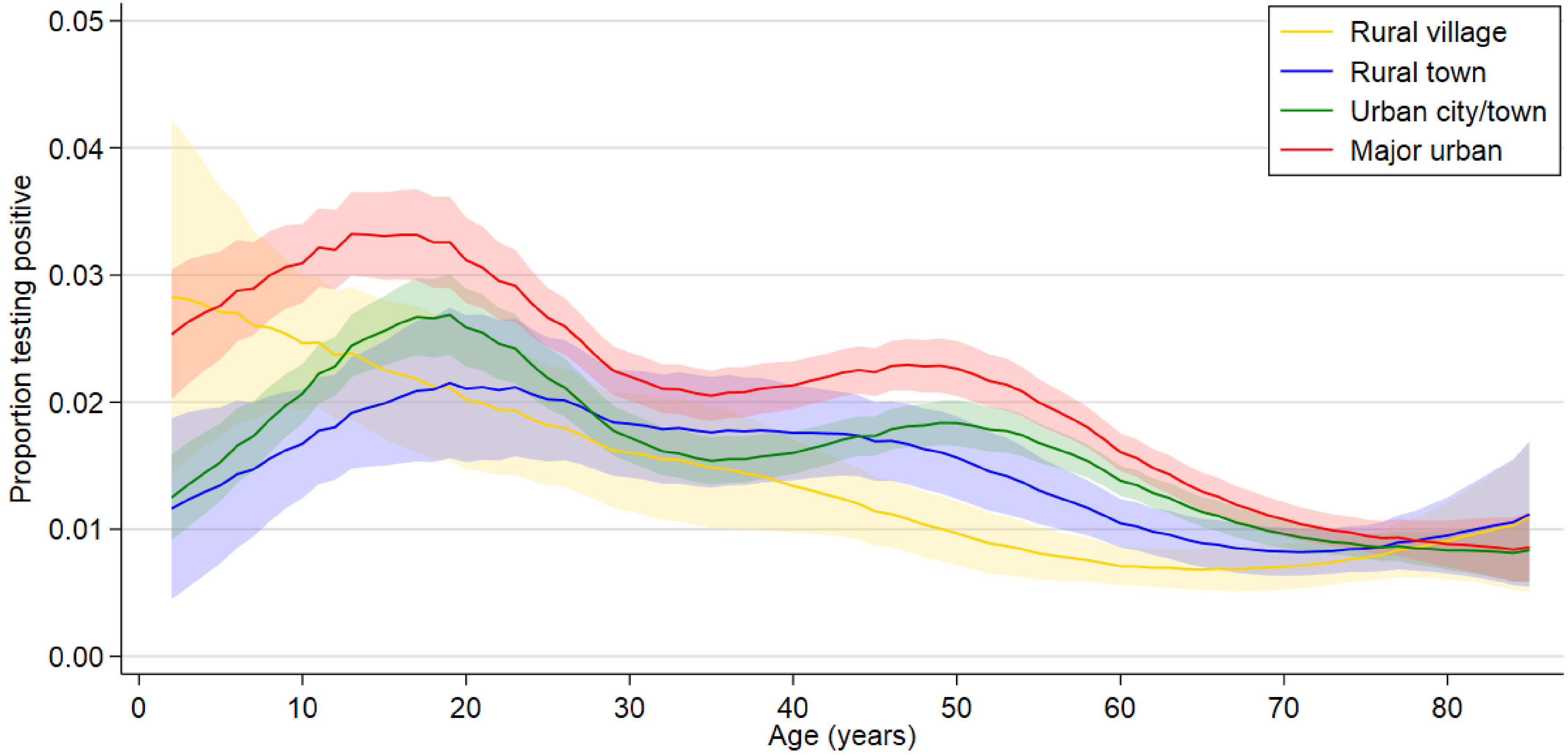

Note: effects marginalised over other variables.

Figure 13F: Effect of interaction of region by household size in the 28-day period 6th December 2020 to 2nd January 2021

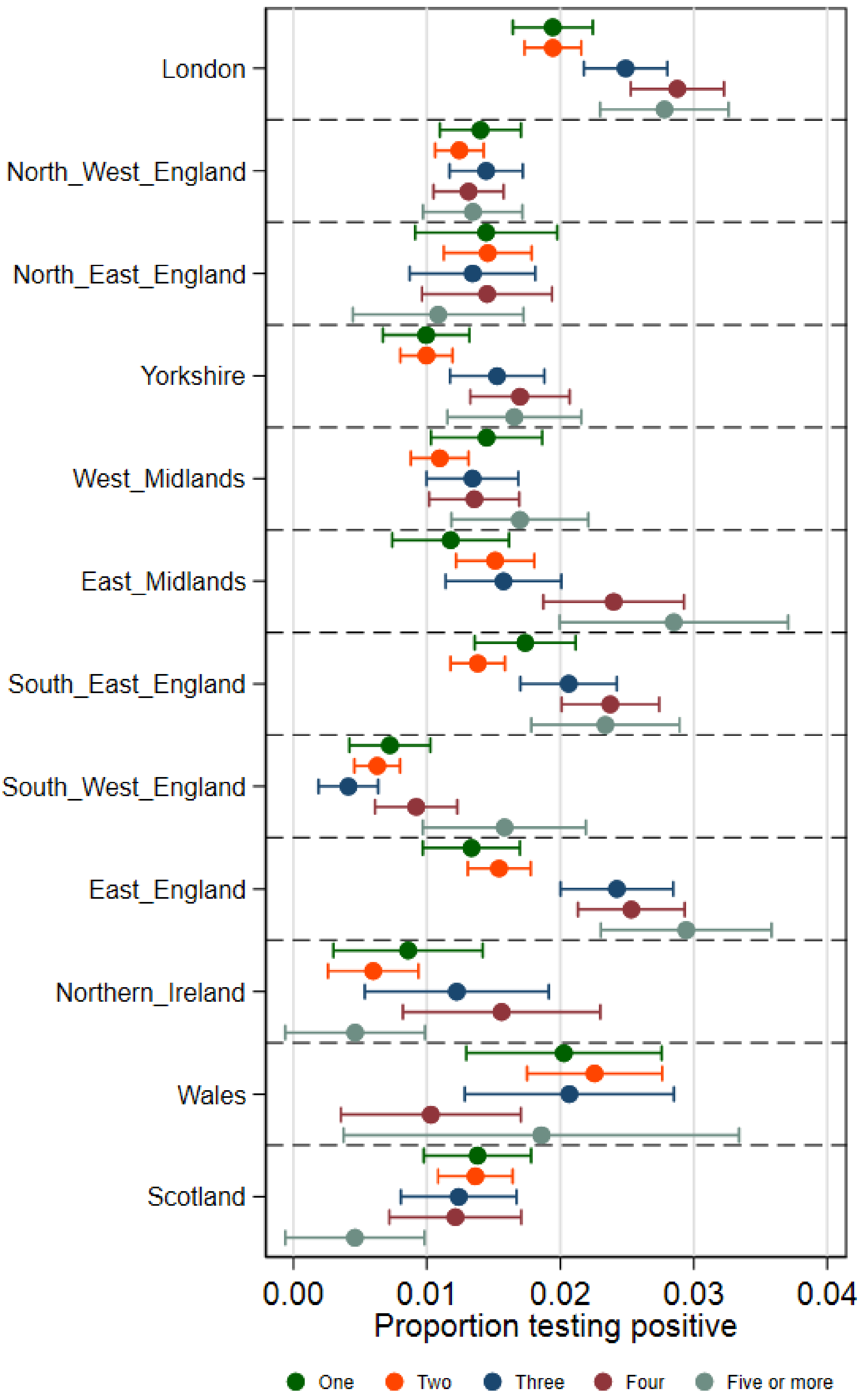

Figure 13G: Effect of interaction of ethnicity by multigenerational households in the 28-day period 31st January 2021 to 27th February 2021

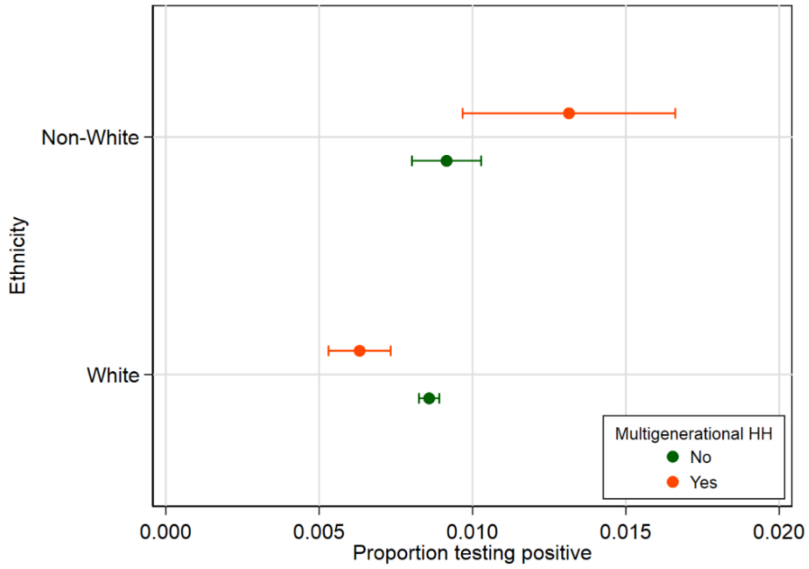

Supplementary Figure 14A: Global heterogeneity p-values per factor from the screening process for 28-day periods for characetrics based on work, health status and contacts

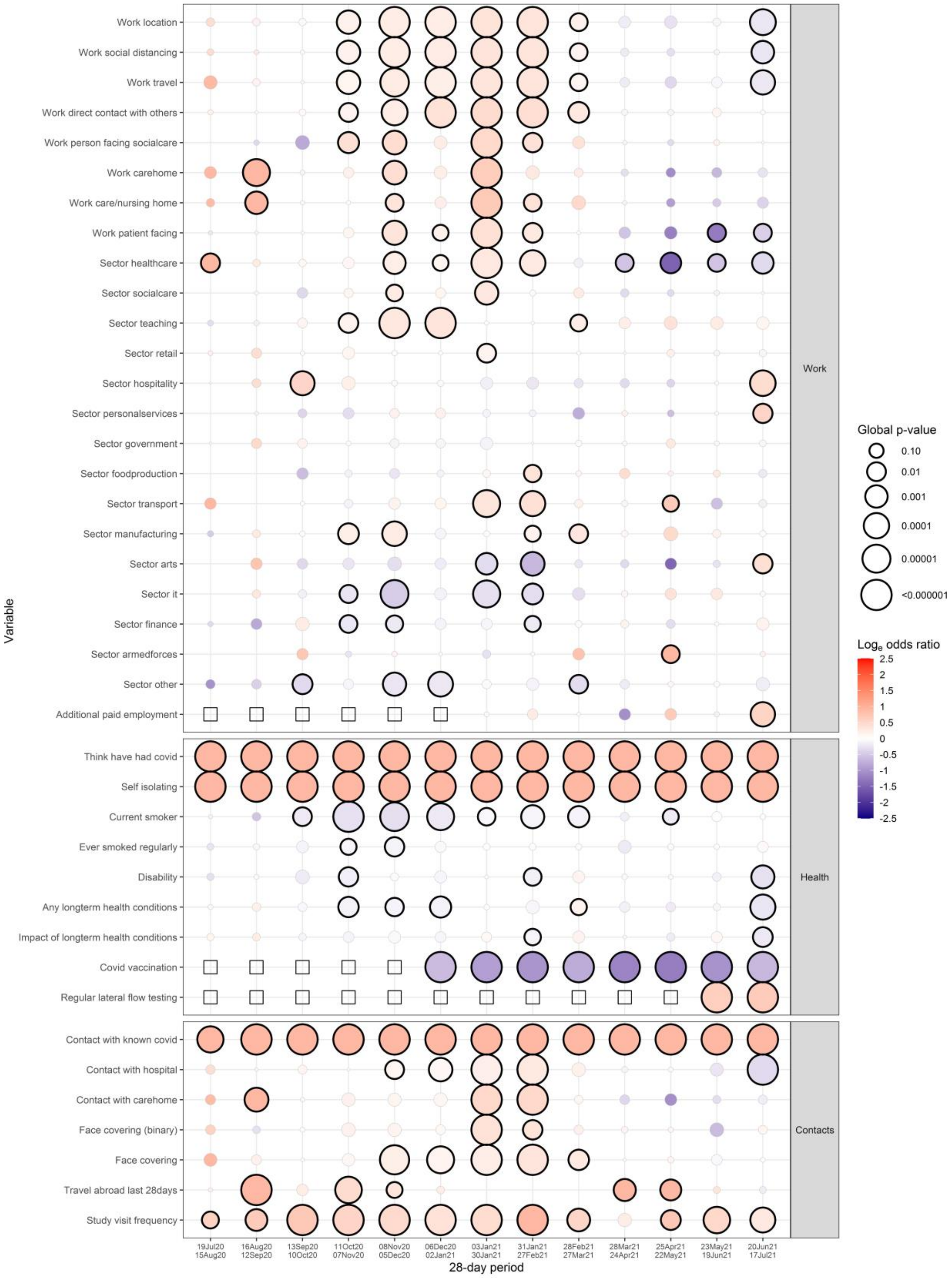

Supplementary Figure 14B: Global heterogeneity p-values per factor from the screening process for 28-day periods for characteristics based on household and living environment

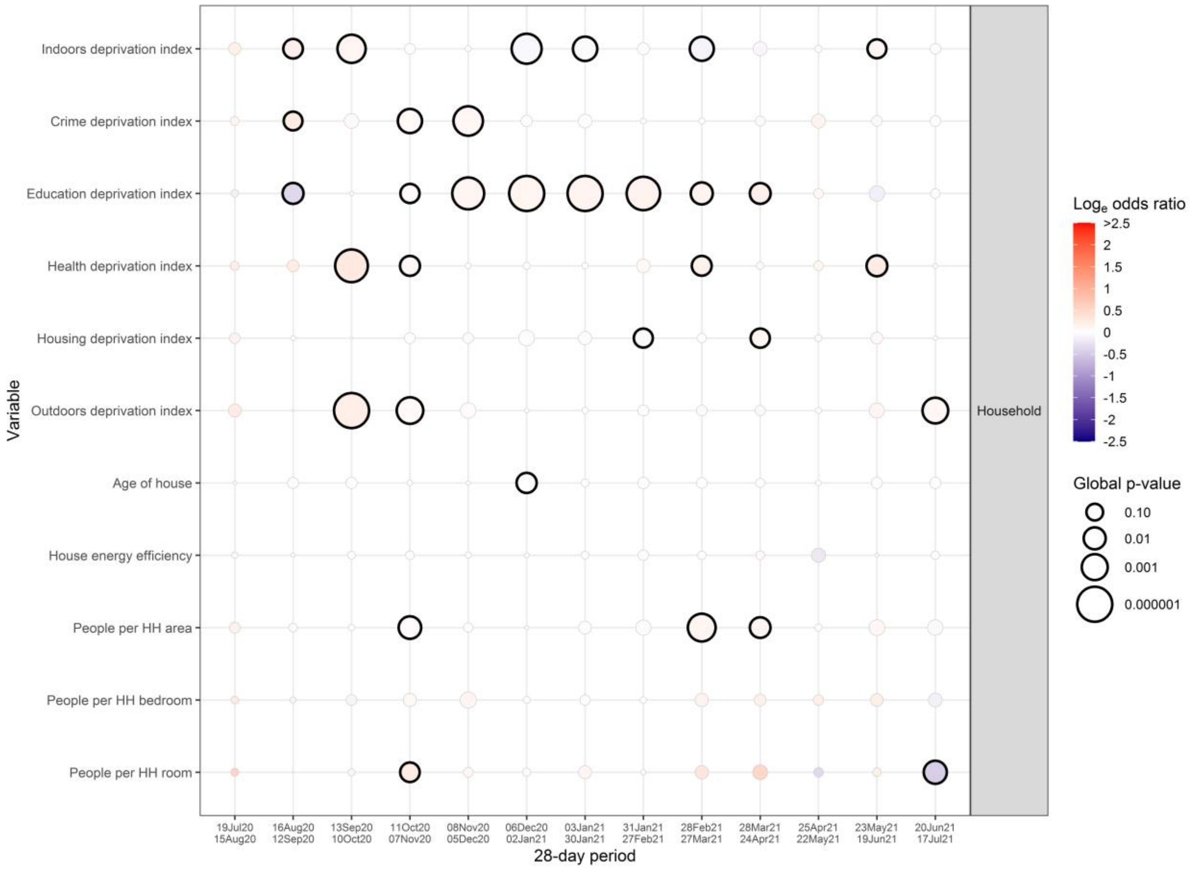

Note: each factor included in addition to the core variables in each period. See **Supplementary Table 1** for variable names and distributions.

Supplementary Figure 15: Comparison of odds ratios and p-values for work location and work social distancing using reported answer from current visit (original) and using imputed values (see note below)

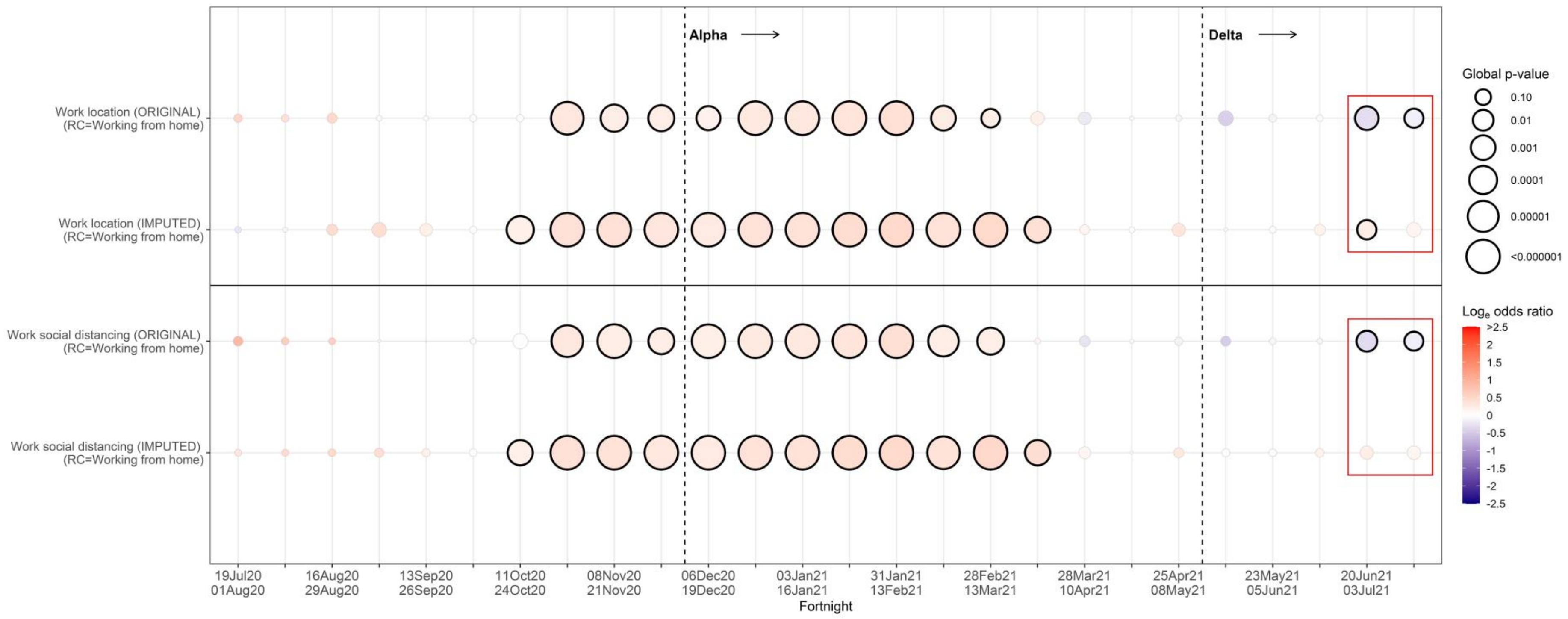

Note: Work location was imputed as "working outside of home" if reported at any time in the 35 days prior to current visit. Work social distancing was imputed as the hardest level of social distancing in the workplace reported in the 35 days prior to current visit. Values in the current visit were used if participants had no visits in the prior 35 days, or all participant visits in the prior 35 days reported "working from home". Red boxes indicate fortnights where reverse causality persisted using the original values, and where imputation resolved reverse causality issues.

Supplementary Figure 16: Results from ridge regression and logistic regression

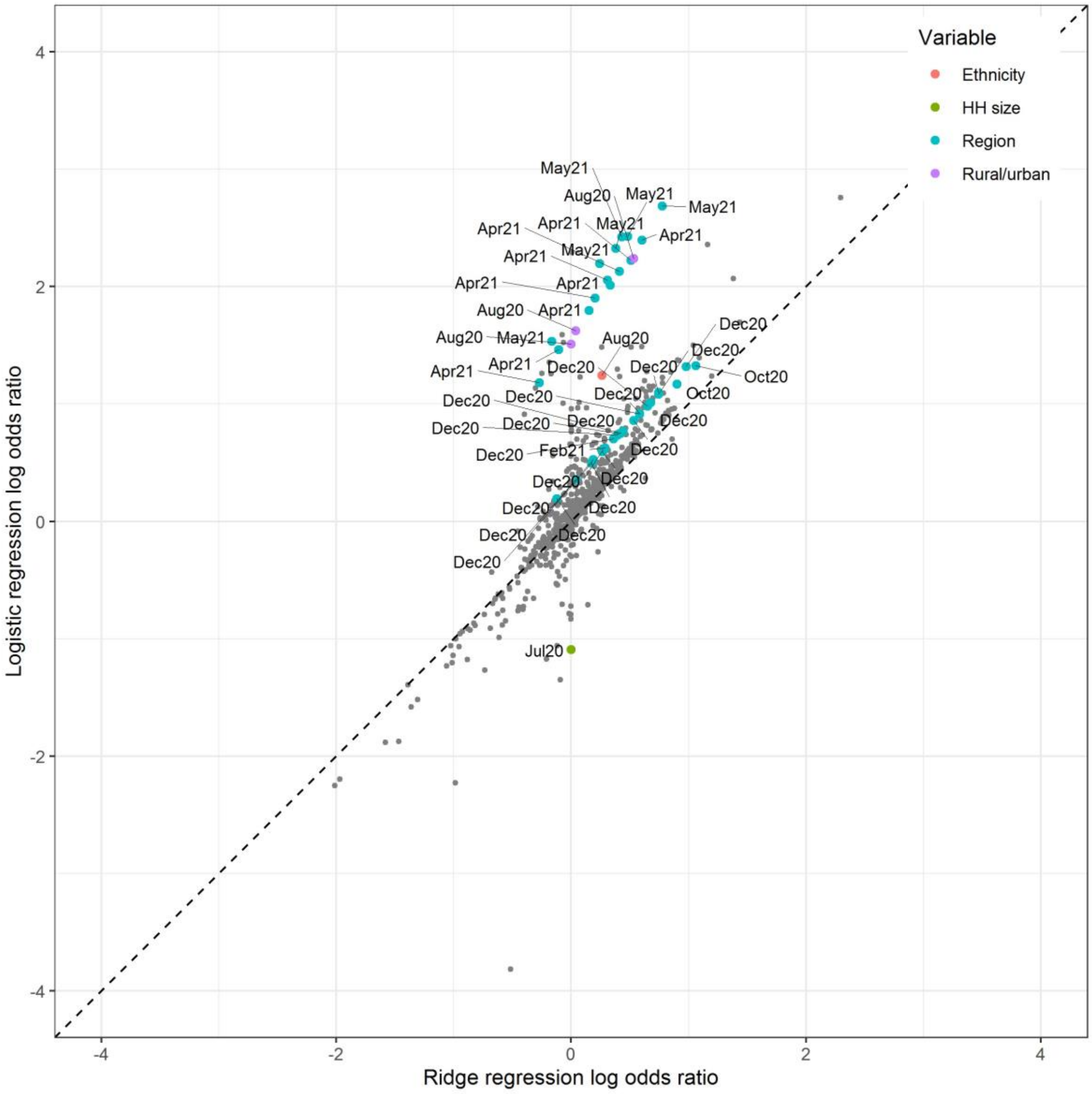

	Ridge coefficients outside of logistic regression 95% confidence interval, n (%)
**Total**	43 (6% of all 692 coefficients)
**By Variable**	
Region	38 (88)
Rural/Urban Classification	3 (7)
Household size	1 (2)
Ethnicity	1 (2)
**By fortnight**	
19Jul20-01Aug20	1 (2)
16Aug20-29Aug20	1 (2)
30Aug20-12Sep20	3 (7)
11Oct20-24Oct20	2 (5)
06Dec20-19Dec20	10 (23)
20Dec20-02Jan21	10 (23)
14Feb21-27Feb21	1 (2)
11Apr21-24Apr21	9 (21)
09May21-22May21	6 (14)

Supplementary Figure 17: Global hetergeneity p-values per factor from the screening process over all 26 fortnights

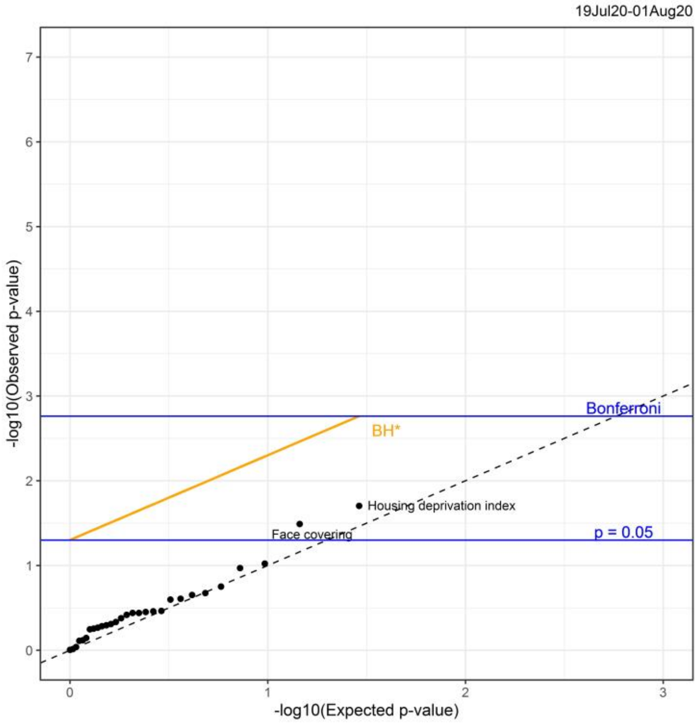

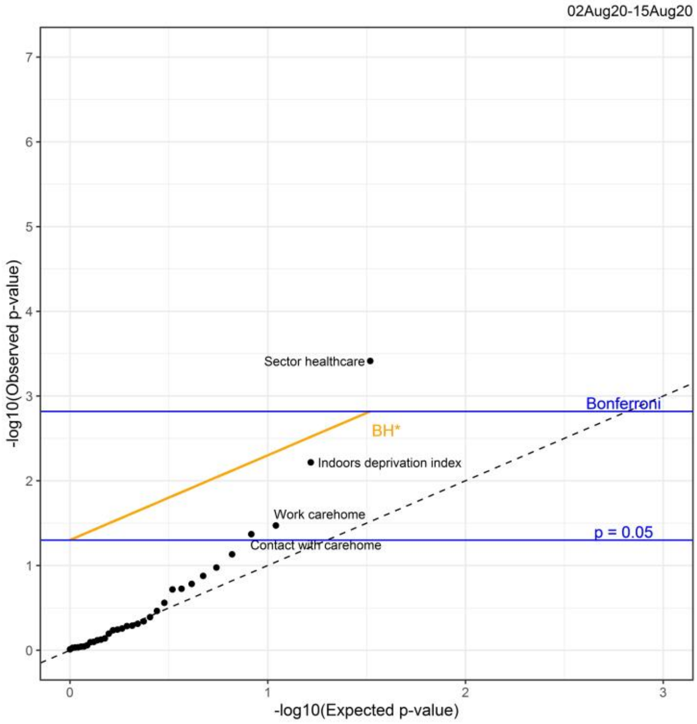

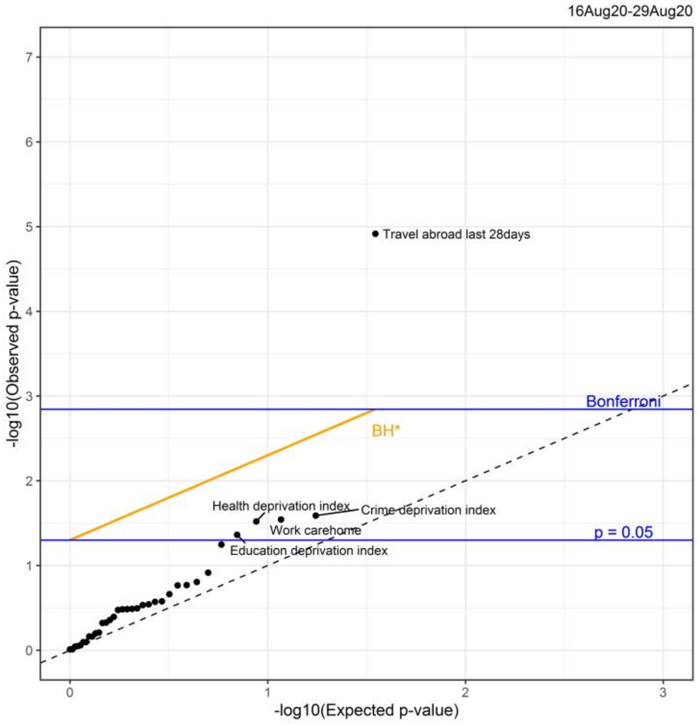
16Aug20-29Aug20
-log10(Observed p-value)
-log10(Expected p-value)
Travel abroad last 28days
Bonferroni
BH*
Health deprivation index
Crime deprivation index
Work carehome
p = 0.05
Education deprivation index

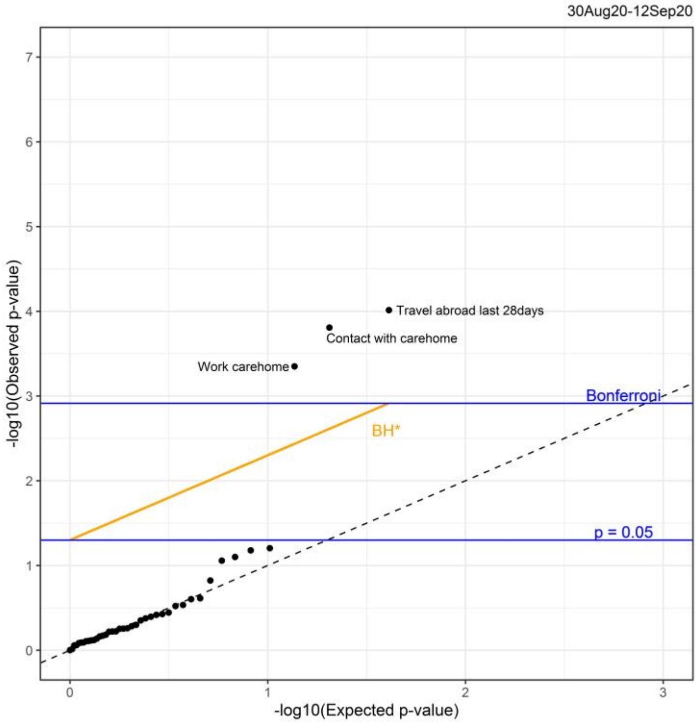
30Aug20-12Sep20
-log10(Observed p-value)
-log10(Expected p-value)
Travel abroad last 28days
Contact with carehome
Work carehome
Bonferroni
BH*
p = 0.05

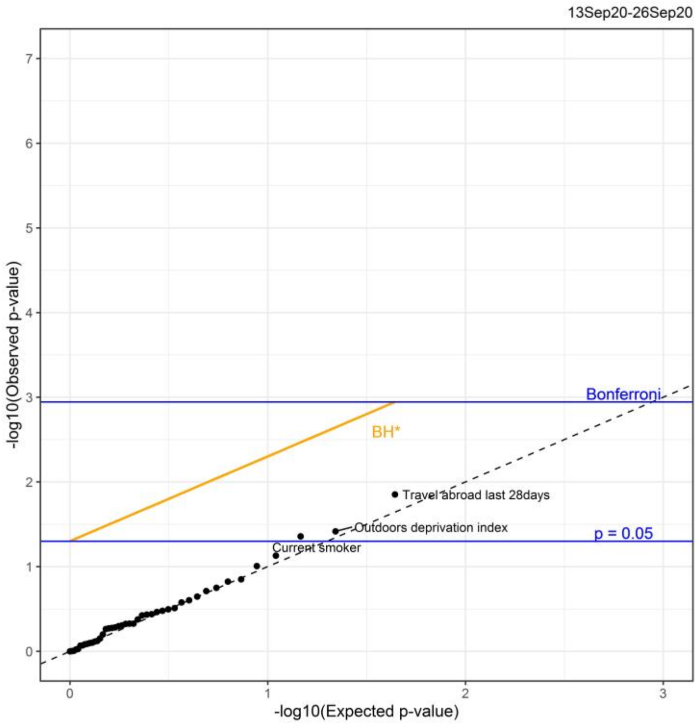
13Sep20-26Sep20
-log10(Observed p-value)
-log10(Expected p-value)
Bonferroni
BH*
p = 0.05
Travel abroad last 28days
Outdoors deprivation index
Current smoker

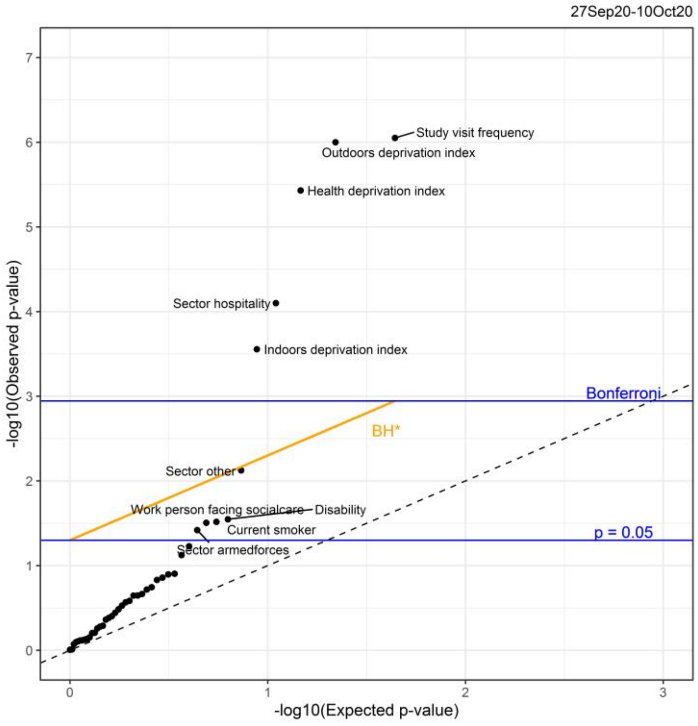
27Sep20-10Oct20
-log10(Observed p-value)
-log10(Expected p-value)
Study visit frequency
Outdoors deprivation index
Health deprivation index
Sector hospitality
Indoors deprivation index
Bonferroni
BH*
Sector other
Work person facing socialcare
Disability
Current smoker
p = 0.05
Sector armedforces

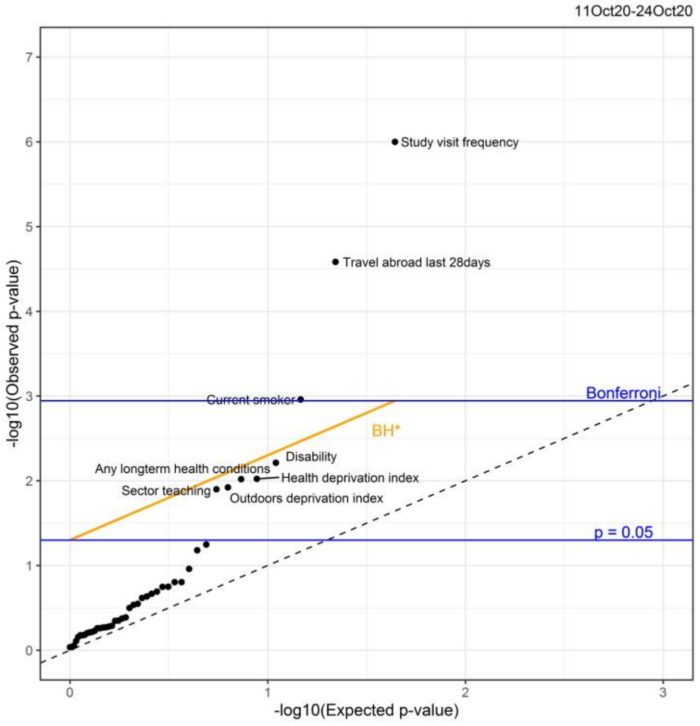
11Oct20-24Oct20
Study visit frequency
Travel abroad last 28days
Current smoker
Bonferroni
BH*
Disability
Any longterm health conditions
Health deprivation index
Sector teaching
Outdoors deprivation index
p = 0.05
-log10(Observed p-value)
-log10(Expected p-value)

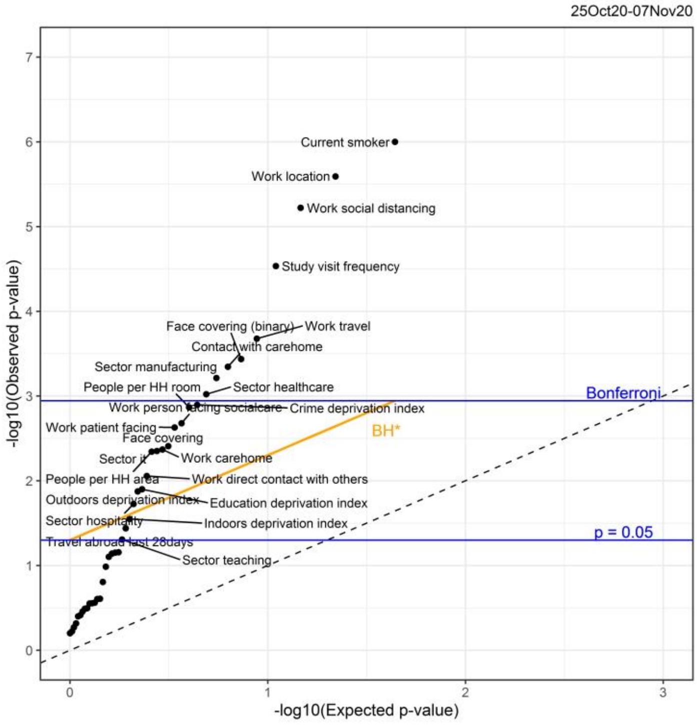
25Oct20-07Nov20
Current smoker
Work location
Work social distancing
Study visit frequency
Face covering (binary)
Work travel
Contact with carehome
Sector manufacturing
People per HH room
Sector healthcare
Bonferroni
Work person facing socialcare
Crime deprivation index
Work patient facing
BH*
Face covering
Sector it
Work carehome
People per HH area
Work direct contact with others
Outdoors deprivation index
Education deprivation index
Sector hospitality
Indoors deprivation index
Travel abroad last 28days
p = 0.05
Sector teaching
-log10(Observed p-value)
-log10(Expected p-value)

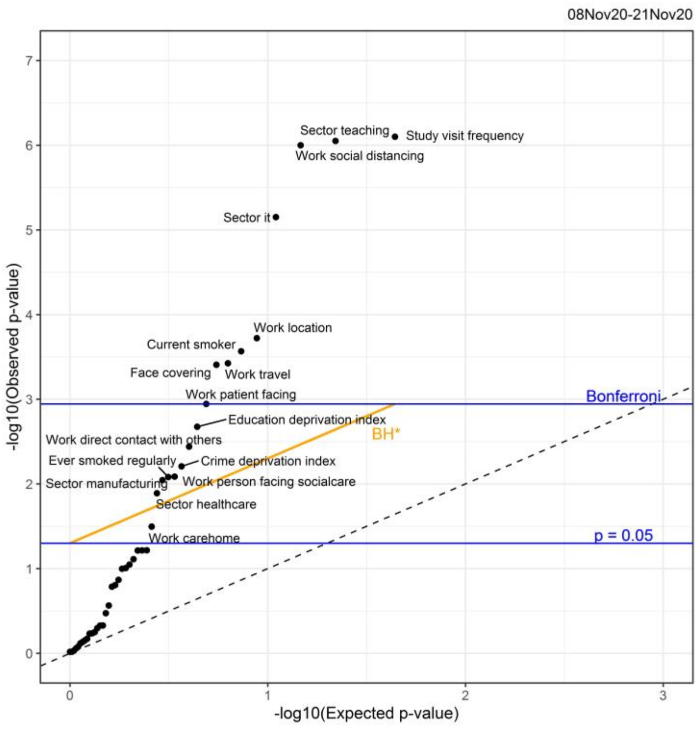

08Nov20-21Nov20
Sector teaching
Study visit frequency
Work social distancing
Sector it
Work location
Current smoker
Face covering
Work travel
Work patient facing
Bonferroni
Education deprivation index
BH*
Work direct contact with others
Ever smoked regularly
Crime deprivation index
Sector manufacturing
Work person facing socialcare
Sector healthcare
Work carehome
p = 0.05
-log10(Observed p-value)
-log10(Expected p-value)

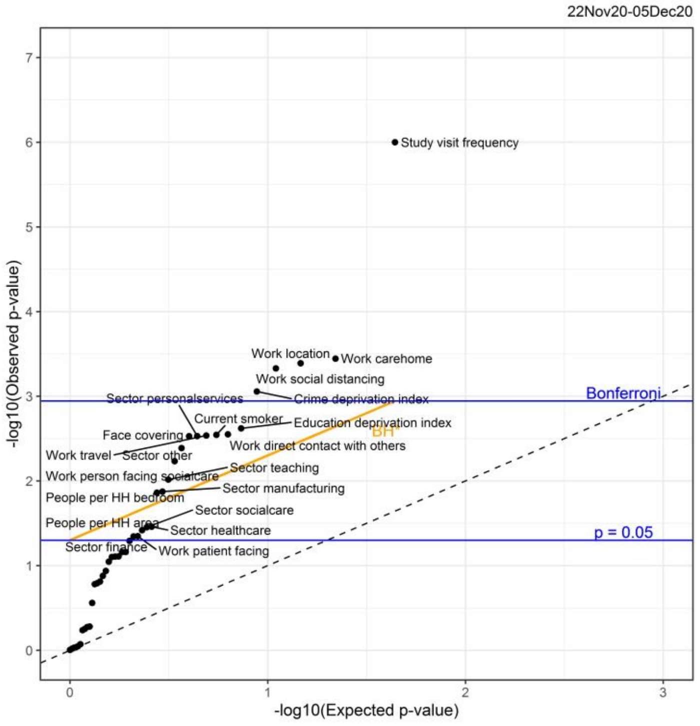

22Nov20-05Dec20
Study visit frequency
Work location
Work carehome
Work social distancing
Sector personalservices
Crime deprivation index
Bonferroni
Current smoker
Education deprivation index
Face covering
BH*
Work direct contact with others
Work travel
Sector other
Sector teaching
Work person facing socialcare
Sector manufacturing
People per HH bedroom
Sector socialcare
People per HH area
Sector healthcare
p = 0.05
Sector finance
Work patient facing
-log10(Observed p-value)
-log10(Expected p-value)

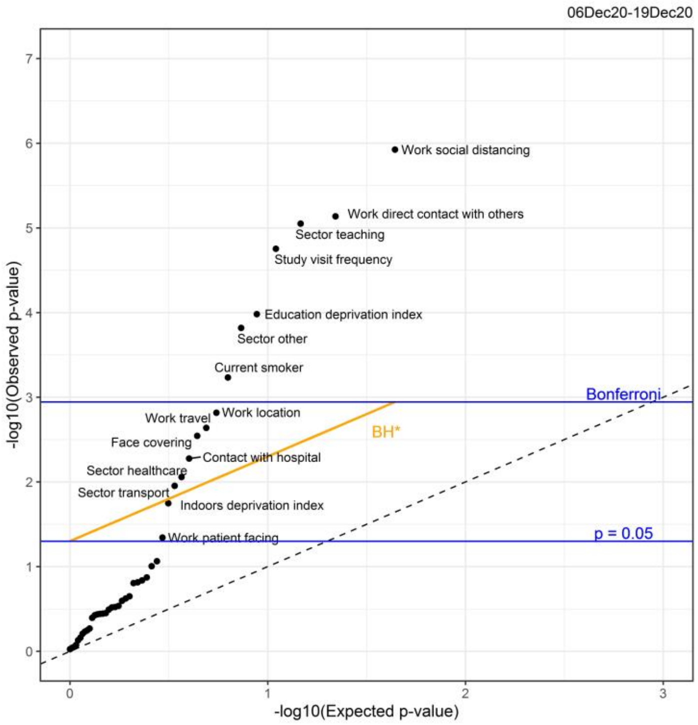
06Dec20-19Dec20
Work social distancing
Work direct contact with others
Sector teaching
Study visit frequency
Education deprivation index
Sector other
Current smoker
Bonferroni
Work travel
Work location
BH*
Face covering
Contact with hospital
Sector healthcare
Sector transport
Indoors deprivation index
Work patient facing
p = 0.05
-log10(Observed p-value)
-log10(Expected p-value)

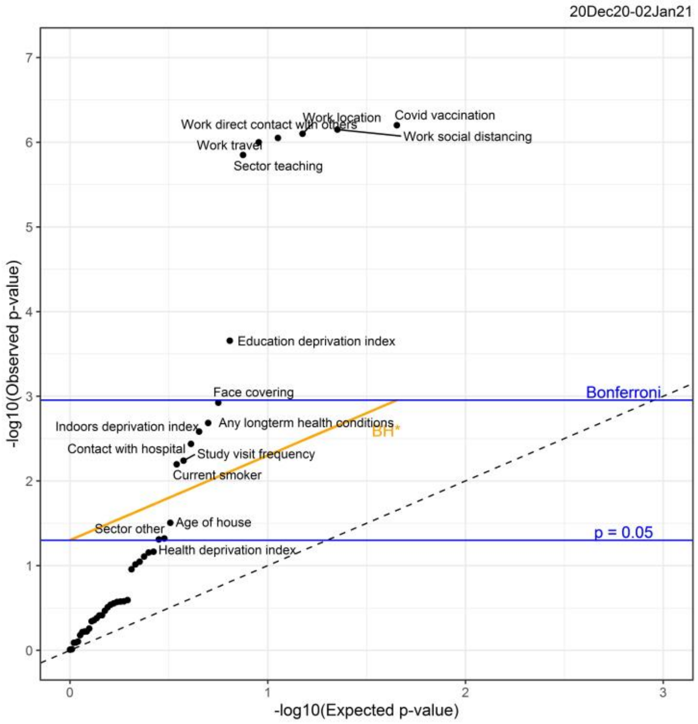
20Dec20-02Jan21
Covid vaccination
Work location
Work direct contact with others
Work social distancing
Work travel
Sector teaching
Education deprivation index
Face covering
Bonferroni
Indoors deprivation index
Any longterm health conditions
BH*
Contact with hospital
Study visit frequency
Current smoker
Age of house
Sector other
p = 0.05
Health deprivation index
-log10(Observed p-value)
-log10(Expected p-value)

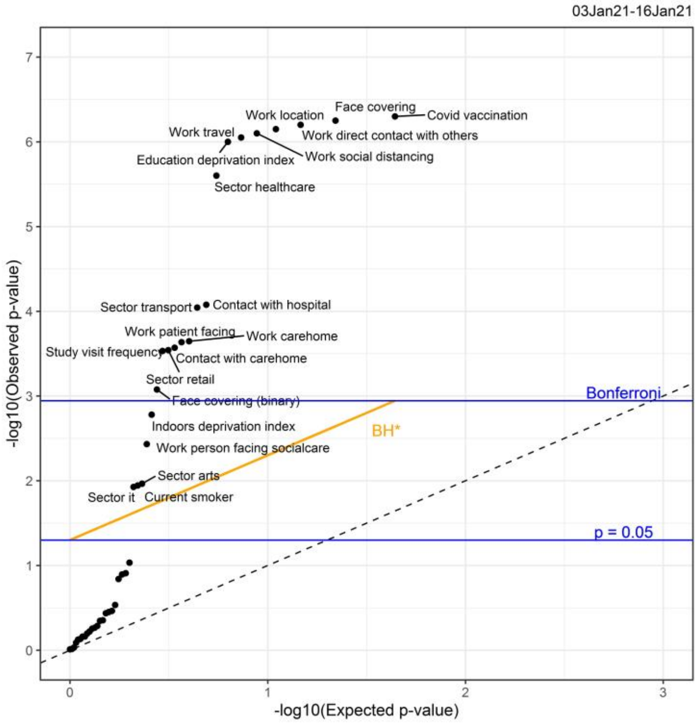

03Jan21-16Jan21
Face covering
Covid vaccination
Work location
Work direct contact with others
Work travel
Work social distancing
Education deprivation index
Sector healthcare
Sector transport
Contact with hospital
Work patient facing
Work carehome
Study visit frequency
Contact with carehome
Sector retail
Face covering (binary)
Bonferroni
Indoors deprivation index
BH*
Work person facing socialcare
Sector arts
Sector it
Current smoker
p = 0.05
-log10(Observed p-value)
-log10(Expected p-value)

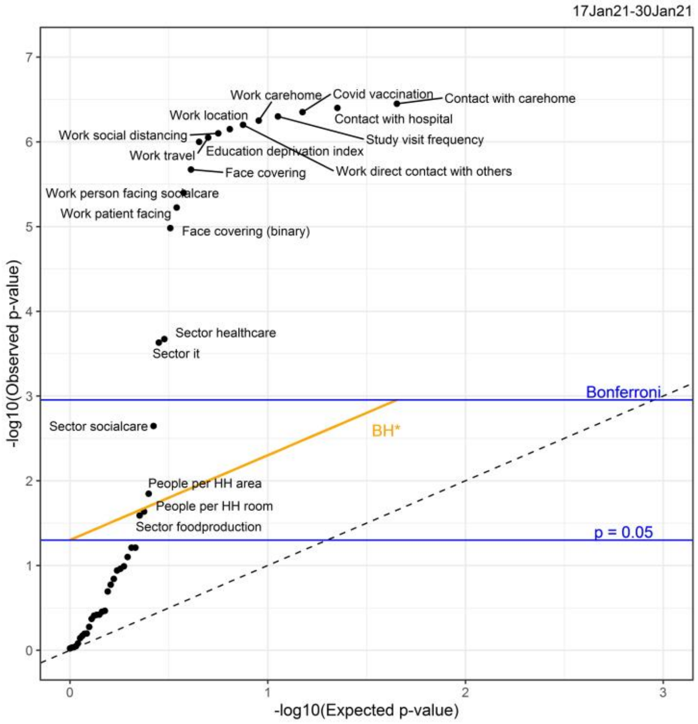

17Jan21-30Jan21
Work carehome
Covid vaccination
Contact with carehome
Work location
Contact with hospital
Work social distancing
Study visit frequency
Work travel
Education deprivation index
Face covering
Work direct contact with others
Work person facing socialcare
Work patient facing
Face covering (binary)
Sector healthcare
Sector it
Bonferroni
Sector socialcare
BH*
People per HH area
People per HH room
Sector foodproduction
p = 0.05
-log10(Observed p-value)
-log10(Expected p-value)

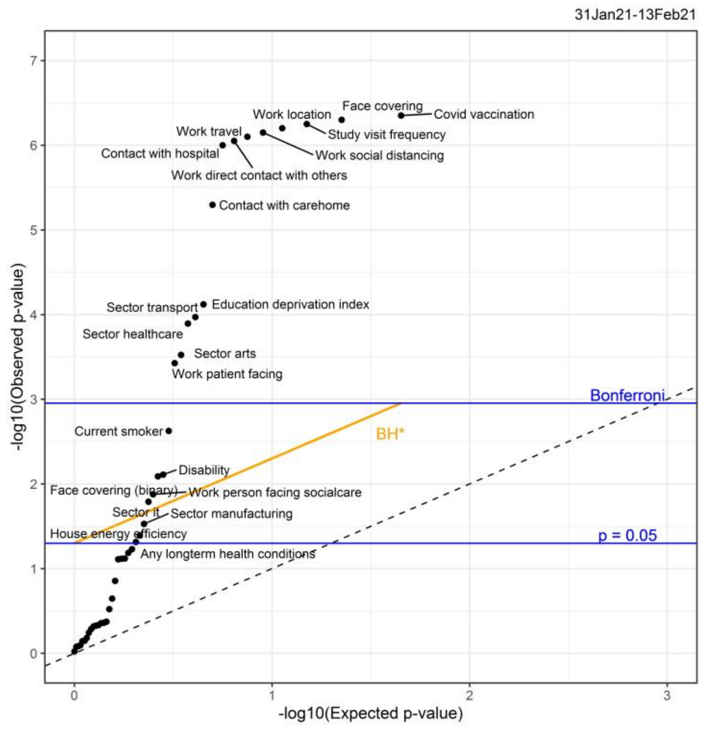
31Jan21-13Feb21
-log10(Observed p-value)
-log10(Expected p-value)
Face covering
Covid vaccination
Work location
Study visit frequency
Work travel
Contact with hospital
Work social distancing
Work direct contact with others
Contact with carehome
Sector transport
Education deprivation index
Sector healthcare
Sector arts
Work patient facing
Bonferroni
Current smoker
BH*
Disability
Face covering (binary)
Work person facing socialcare
Sector it
Sector manufacturing
House energy efficiency
p = 0.05
Any longterm health conditions

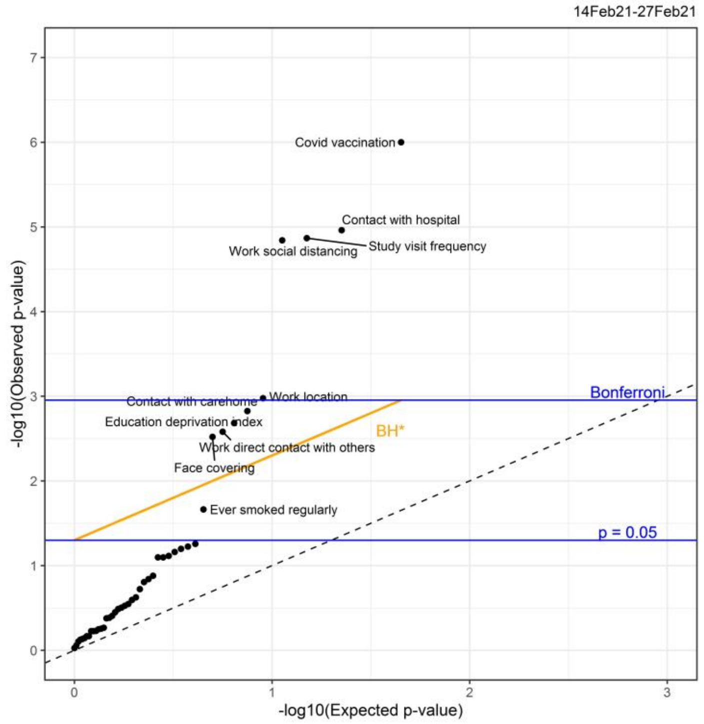
14Feb21-27Feb21
-log10(Observed p-value)
-log10(Expected p-value)
Covid vaccination
Contact with hospital
Work social distancing
Study visit frequency
Contact with carehome
Work location
Bonferroni
Education deprivation index
BH*
Work direct contact with others
Face covering
Ever smoked regularly
p = 0.05

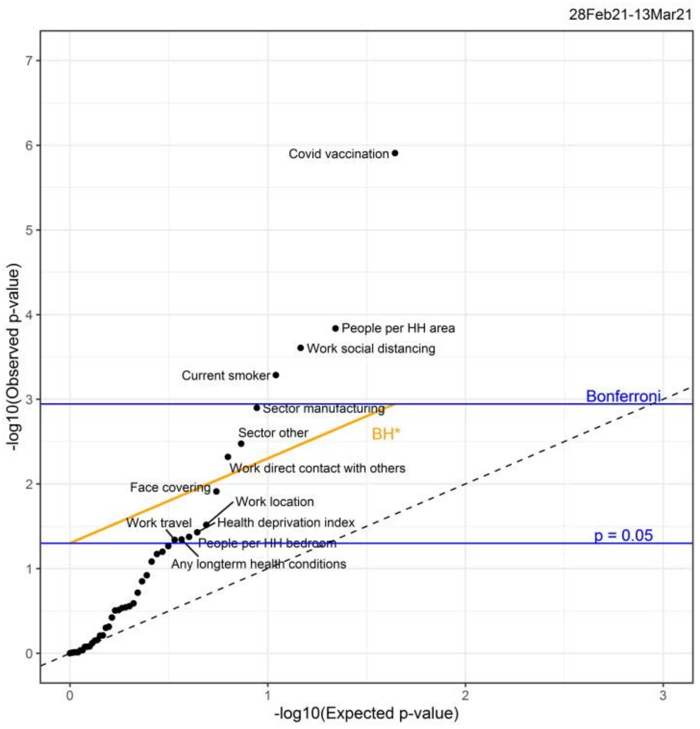
28Feb21-13Mar21
-log10(Observed p-value)
-log10(Expected p-value)
Covid vaccination
People per HH area
Work social distancing
Current smoker
Bonferroni
Sector manufacturing
Sector other
BH*
Work direct contact with others
Face covering
Work location
Work travel
Health deprivation index
People per HH bedroom
p = 0.05
Any longterm health conditions

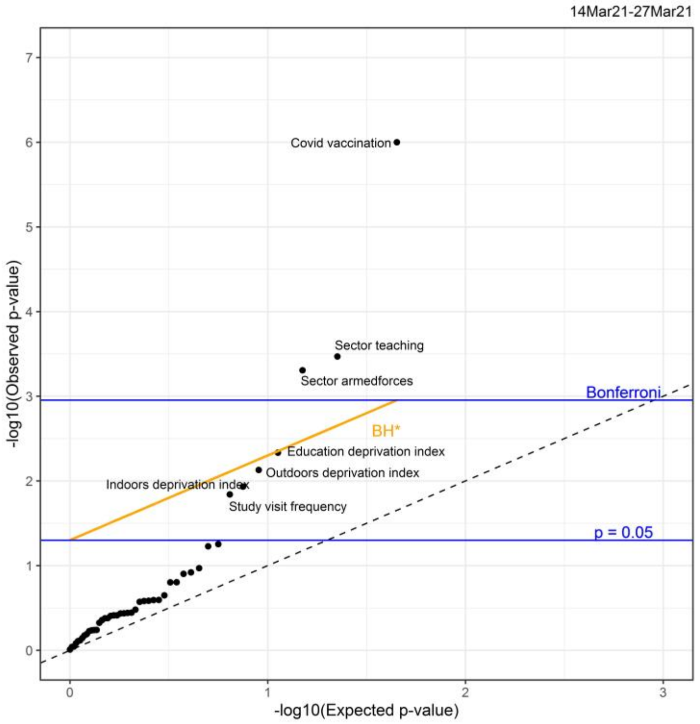
14Mar21-27Mar21
-log10(Observed p-value)
-log10(Expected p-value)
Covid vaccination
Sector teaching
Sector armedforces
Bonferroni
BH*
Education deprivation index
Outdoors deprivation index
Indoors deprivation index
Study visit frequency
p = 0.05

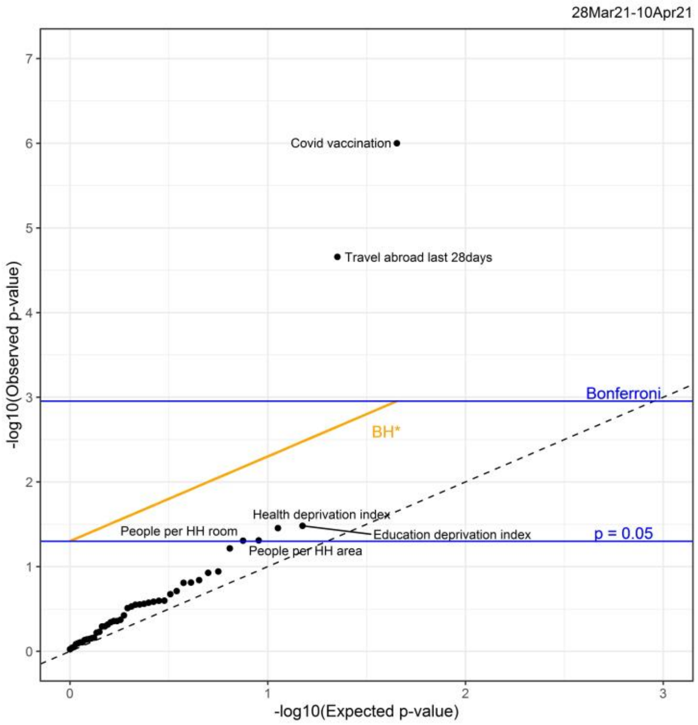
28Mar21-10Apr21
Covid vaccination
Travel abroad last 28days
Bonferroni
BH*
Health deprivation index
People per HH room
Education deprivation index
p = 0.05
People per HH area
-log10(Observed p-value)
-log10(Expected p-value)

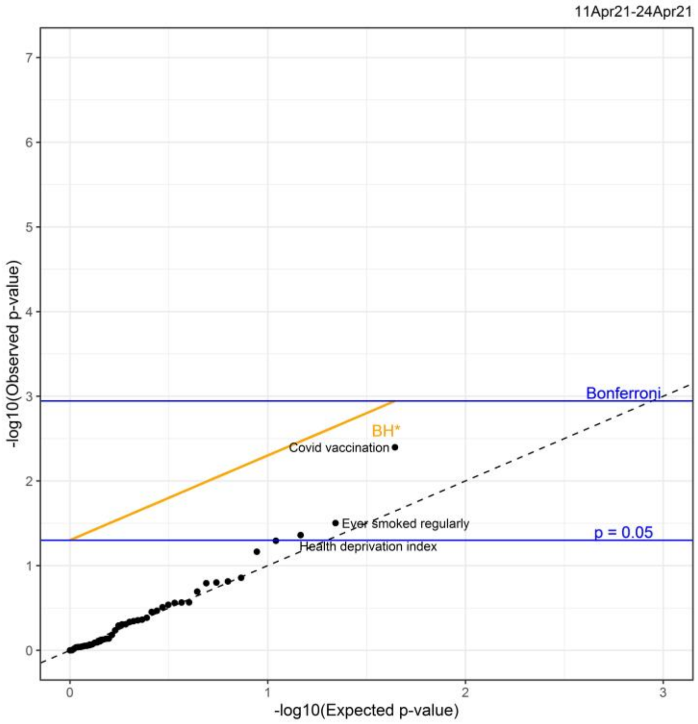
11Apr21-24Apr21
Bonferroni
BH*
Covid vaccination
Ever smoked regularly
p = 0.05
Health deprivation index
-log10(Observed p-value)
-log10(Expected p-value)

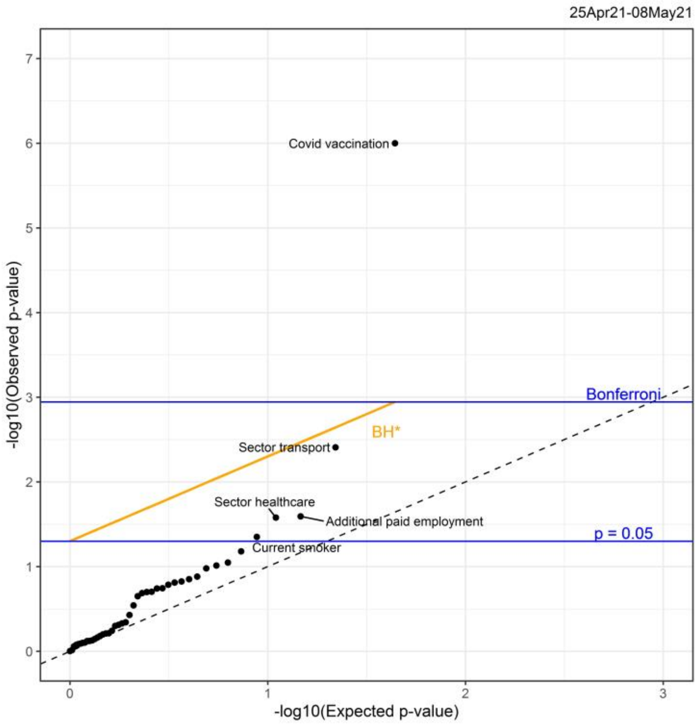
25Apr21-08May21
-log10(Observed p-value)
-log10(Expected p-value)
Covid vaccination
Bonferroni
BH*
Sector transport
Sector healthcare
Additional paid employment
p = 0.05
Current smoker

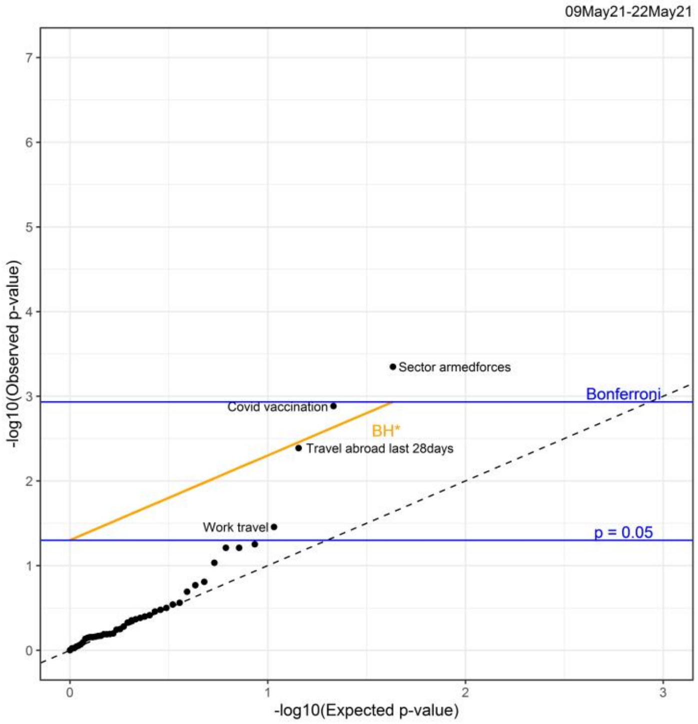
09May21-22May21
-log10(Observed p-value)
-log10(Expected p-value)
Sector armedforces
Bonferroni
Covid vaccination
BH*
Travel abroad last 28days
Work travel
p = 0.05

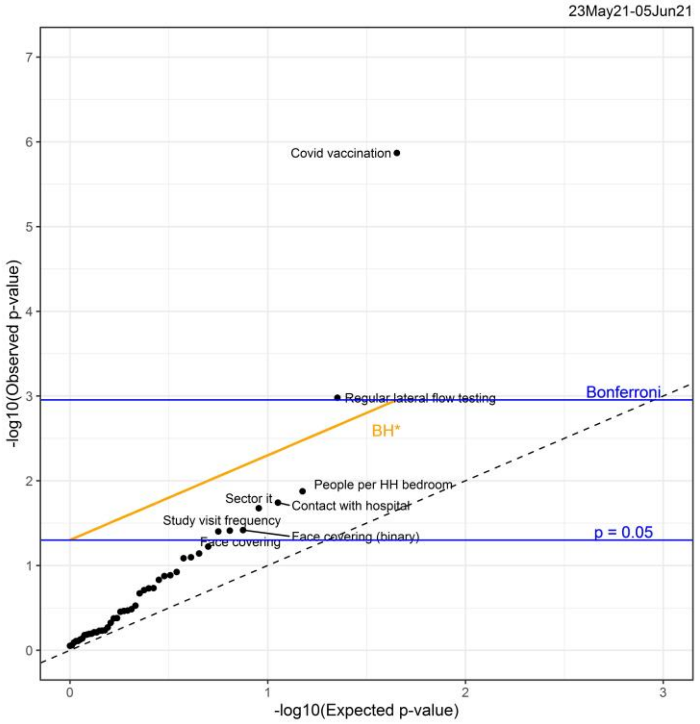
23May21-05Jun21
-log10(Observed p-value)
-log10(Expected p-value)
Covid vaccination
Regular lateral flow testing
Bonferroni
BH*
People per HH bedroom
Sector it
Contact with hospital
Study visit frequency
Face covering (binary)
Face covering
p = 0.05

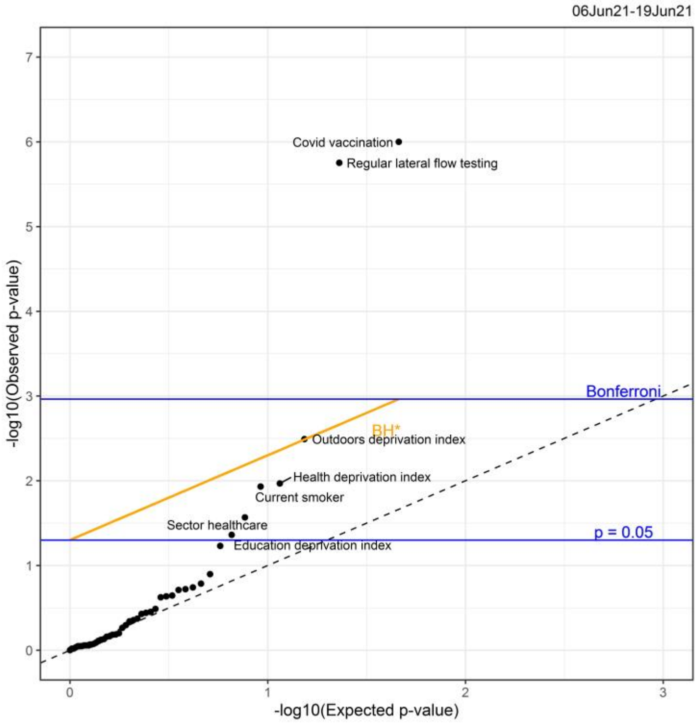
06Jun21-19Jun21
-log10(Observed p-value)
-log10(Expected p-value)
Covid vaccination
Regular lateral flow testing
Bonferroni
BH*
Outdoors deprivation index
Health deprivation index
Current smoker
Sector healthcare
Education deprivation index
p = 0.05

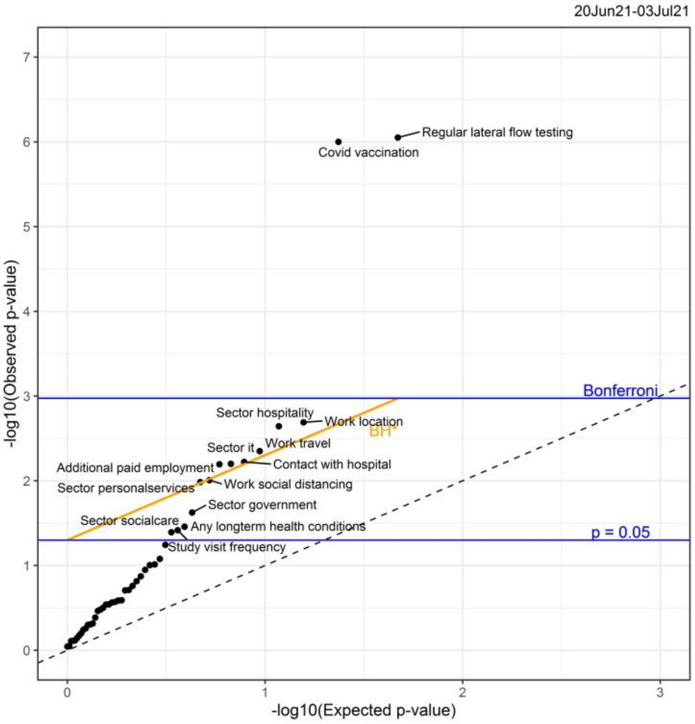

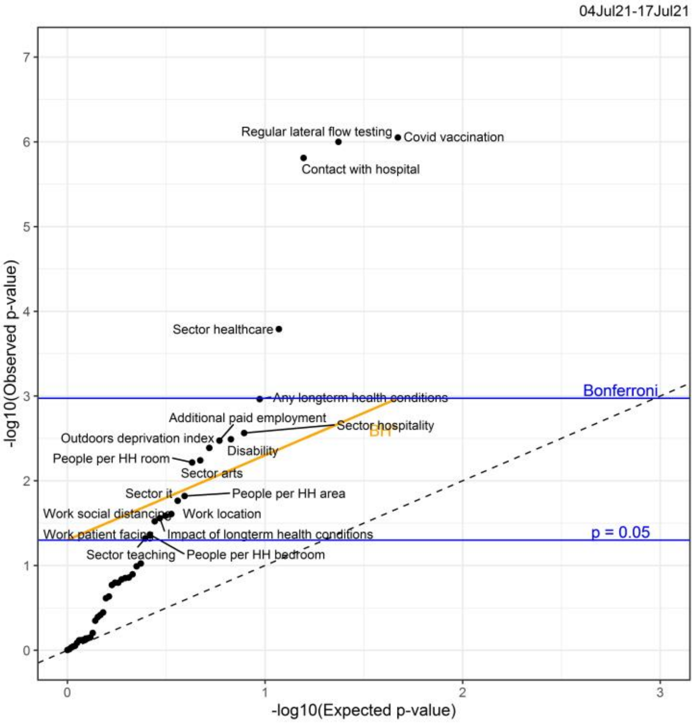

Note: Black dashed line shows y = x. see **Supplementary Table 1** for variable names and distributions
